# Integrated single-cell and bulk RNA sequencing analyses identify a myeloid state-related gene signature for molecular subtyping in stomach adenocarcinoma

**DOI:** 10.3389/fimmu.2026.1887429

**Published:** 2026-07-16

**Authors:** Ruinan Li, Bohong Wei, Bin Sun, Mingji Li, Yingman Wang, Xiangyu Zhao, Yuntao Yao, Duowu Zou, Zirui He

**Affiliations:** 1 Department of Gastroenterology, Ruijin Hospital, School of Medicine, Shanghai Jiao Tong University, Shanghai, China; 2 Shanghai Jiao Tong University School of Medicine, Shanghai, China; 3 Department of General Surgery, Ruijin Hospital, School of Medicine, Shanghai Jiao Tong University, Shanghai, China; 4 Department of Urology, Shanghai Jiaotong University School of Medicine Xinhua Hospital, Shanghai, China

**Keywords:** immunohistochemistry, molecular classification system, myeloid cells, single-cell sequencing, stomach adenocarcinoma (STAD)

## Abstract

**Purpose:**

Stomach adenocarcinoma (STAD) is characterized by significant heterogeneity, within which myeloid cells play crucial yet incompletely understood roles. The relationship between the functional states of myeloid cells, patient prognosis, and therapeutic response requires further elucidation.

**Methods:**

We integrated single-cell RNA-seq profiles and 443 bulk RNA-seq profiles from the TCGA-STAD cohort. By integrating myeloid cell differentiation trajectories inferred from Monocle2 pseudotime analysis with survival analysis, we identified myeloid state-related prognostic genes (MSRPGs) and constructed a molecular classification (STAD-MSC). We also explored its prognostic significance and multi-omics features. Additionally, we utilized correlation analysis to establish regulatory networks and predict candidate inhibitors. The 5-gene risk model was evaluated in a public 355-patient validation cohort, and the STAD-MSC framework was further assessed at the protein level in a 70-patient retrospective cohort using immunohistochemistry for NNMT, AXL, and COL1A1.

**Results:**

We identified 32 MSRPGs across five distinct myeloid states. Consensus clustering stratified the patients into three subtypes, including low immune infiltration STAD (LI-STAD), moderate immune infiltration STAD (MI-STAD), and high immune infiltration STAD (HI-STAD). The HI-STAD subtype, characterized by high immune infiltration accompanied by an immunosuppressive and dysfunctional microenvironment, exhibited the poorest overall survival (global log-rank p = 0.018). The multi-omics analysis revealed subtype-specific genomic and immune landscapes. A 5-gene prognostic signature was constructed and evaluated as a risk-associated prognostic model. In silico analysis identified subtype-associated differences in predicted drug response. Exploratory pharmacogenomic analysis revealed nominal associations for dabrafenib (p = 0.0051) and ruxolitinib (p = 0.041), suggesting potential subtype−specific therapeutic vulnerabilities. Importantly, the three-protein classifier (NNMT/AXL/COL1A1) stratified a retrospective 70-patient cohort into three subgroups with significantly different OS and PFS.

**Conclusion:**

Using public-cohort and protein-level clinical validation, we established STAD-MSC, a myeloid state-centric molecular taxonomy that stratifies STAD patients into subgroups with distinct prognoses and immunosuppressive microenvironmental features, providing a framework for immune-informed patient stratification.

## 1 Introduction

Stomach adenocarcinoma (STAD) is a prevalent malignancy, ranking as the fifth most commonly diagnosed cancer and the fifth leading cause of cancer-related deaths worldwide. In 2022 alone, it was responsible for over 968,000 new cases and approximately 660,000 fatalities (1). Although recent advancements in endoscopy, imaging modalities, surgical techniques, and anti-cancer therapeutics have improved the overall prognosis for STAD patients, the outcome for those with advanced-stage disease remains poor, with a median survival of less than one year (2, 3). This is primarily due to the limited therapeutic arsenal available and is further compounded by the notable intra- and intertumoral heterogeneity of STAD, which significantly contributes to its unfavorable prognosis (4). However, reliance on histopathological classification alone is insufficient for effective patient stratification for individualized treatment strategies that improve clinical outcomes (5). To advance beyond traditional histology, the Cancer Genome Atlas (TCGA) research network conducted a comprehensive molecular characterization of 295 untreated stomach adenocarcinomas and categorized gastric adenocarcinoma into four molecular subtypes: EBV-positive, microsatellite instability (MSI), chromosomal instability (CIN), and genomically stable tumors (6). While this framework provides crucial molecular insights, there remains a pressing demand for novel classification and risk assessment methodologies that more directly reflect the dynamic tumor microenvironment (TME) and its role in shaping therapeutic sensitivity and clinical outcomes.

Tumors are complex ecosystems where heterogeneous malignant cells interact with both immune and non-immune cells to shape the complex cellular network of the TME (7). Among the immune constituents, myeloid cells are a pervasive and highly plastic component of the TME. In cancer, these cells infiltrate tumors and play important roles in modulating tumor inflammation and angiogenesis, often adopting immunosuppressive or pro-tumoral functional states (8, 9). Tumor-infiltrating myeloid cells consist of several distinct major lineages, including mast cells, plasmacytoid dendritic cells (pDCs), conventional dendritic cells (cDCs), monocytes, and macrophages (9). Tumor-associated macrophages (TAMs) are a heterogeneous cell type that can contribute to malignancy through the production of tumor and angiogenic growth factors, extracellular matrix (ECM) remodeling, and immunosuppression (10). Dendritic cells (DCs) are key players in antigen-specific immune response. Two distinct cDC subsets, XCR1+CADM1+ cDC1s and CD1A+CD172A+ cDC2s, have been identified and shown to interact with CD8+ and CD4+ T cells, respectively (11–13). Tumor-associated monocytes (TAMos) share phenotypic and functional similarities with monocytic myeloid‐derived suppressor cells, and are capable of hampering antitumor immune responses and promoting tumor progression through their ability to transfer NO and methylglyoxal or to secrete TGF-β, IL‐1β and IL‐10 cytokines (14). In summary, myeloid cell heterogeneity and functional states are closely linked to the progression and immune regulation of STAD. However, how myeloid cell differentiation trajectories within the tumor microenvironment shape immunosuppressive states, patient prognosis, and anti-tumor immunity remains poorly understood.

To address this gap, we integrated single-cell RNA sequencing, bulk transcriptomic data, and multi-omics analyses to construct a myeloid state-based molecular classification for STAD. We first compared myeloid cell populations between tumor and adjacent normal tissues, identifying a set of dysregulated myeloid state-related prognostic genes (MSRPGs) through pseudotime trajectory and survival analysis. Consensus clustering based on MSRPG expression stratified STAD patients into three subtypes with distinct prognostic, immunologic, and genomic characteristics. Furthermore, pharmacogenomic prediction revealed subtype-specific drug sensitivities, highlighting the potential utility of this approach in immune-informed patient stratification and personalized therapeutic prioritization.

## 2 Materials and methods

### 2.1 Data sources

**Figure 1:**
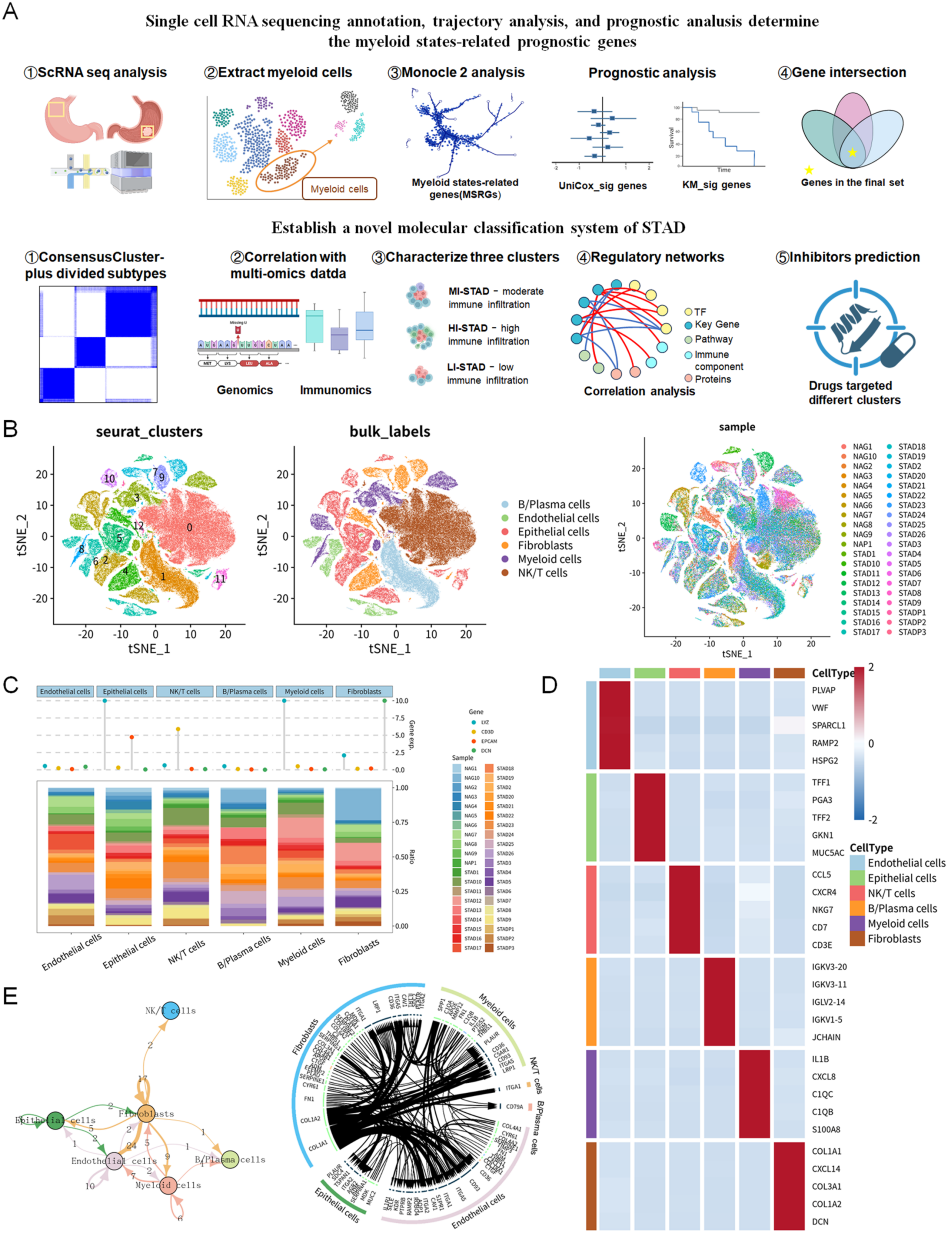
The gene expression landscape of cells in STAD microenvironment. **(A)** The study workflow consisted of single-cell annotation and myeloid cell extraction, pseudotime and prognostic analyses to identify MSRPGs, construction of STAD-MSC subtypes via consensus clustering, and downstream multi-omics characterization and clinical validation, including model development and internal evaluation in the TCGA cohort, external validation in independent public cohorts (GSE62254 and GSE84437), and protein-level validation in a retrospective Ruijin Hospital cohort. **(B)** The UMAP plot shows six annotated cell clusters across 40 samples. **(C)** The Cleveland dot plot depicted the expression levels of the four key marker genes (LYZ, CD3D, EPCAM, DCN) across all 6 cell clusters. **(D)** The heatmap showed the differential expression of the top five DEGs of each cell type, demonstrating the significant heterogeneity of the cells. **(E)** The cellular communication analysis showed that myeloid cells had many interactions with other cells, and the ligand-receptor plot exhibited the corresponding ligand-receptor pairs with the highest significance among the clusters. scRNA-seq, single-cell RNA sequencing; MSRPGs, myeloid state-related prognostic genes; STAD, stomach adenocarcinoma; STAD-MSC, stomach adenocarcinoma myeloid-state classification; UMAP, uniform manifold approximation and projection; MSRG, myeloid state-related genes; OS, overall survival; DEGs, differentially expressed genes.

The scRNA-seq data presented in **Figure 1** were obtained from the Gene Expression Omnibus (GEO) repository under accession number GSE183904 (https://www.ncbi.nlm.nih.gov/geo) (15).

Bulk RNA-seq data and associated clinical information for 443 patients with stomach adenocarcinoma were obtained from TCGA database (https://cancergenome.nih.gov/) (16). The clinical dataset included overall survival (OS), demographic details including age, gender and race, and disease-specific parameters including TNM stage, pathological stage and grade. Transcription factor (TF) profiles were derived from the Cistrome Cancer database (http://cistrome.org/) (17).

Genomic and transcriptomic profiles of cancer cell lines, together with their cytotoxic drug sensitivity data, were obtained from the Cancer Cell Line Encyclopedia (CCLE, https://sites.broadinstitute.org/ccle) (18) and the Genomics of Drug Sensitivity in Cancer (GDSC, https://www.cancerrxgene.org/) databases (19). For over-representation analysis (ORA), a predefined set of fifty molecular signaling pathways, organized into nine broader categories, was retrieved from the Molecular Signatures Database (MSigDB, version 7.4; https://www.gsea-msigdb.org/gsea/msigdb/index.jsp) (20).

### 2.2 Single-cell RNA-seq processing and quality control

The raw single-cell RNA sequencing data in BCL format were first demultiplexed using Cell Ranger (v3.0, 10x Genomics) and aligned to the hg38 reference genome to generate gene−cell matrices, following the original processing workflow of GSE183904. Subsequent processing was performed using a Seurat-based workflow, including quality control, normalization, dimensionality reduction, unsupervised clustering, and differential expression analysis (21, 22). Cell-level quality was assessed using detected gene counts, UMI counts, and mitochondrial RNA content according to the original GSE183904 processing workflow; potential doublets were removed using DoubletFinder. After quality control, each sample was normalized with SCTransform, with mitochondrial content regressed out, followed by anchor-based integration across samples. The top 2000 highly variable genes were then identified using the “vst” selection method. These genes were scaled and standardized prior to dimensionality reduction and clustering. Finally, cellular features were extracted through principal component analysis (PCA) and visualized in two-dimensional space using uniform manifold approximation and projection (UMAP). Cell types were annotated using canonical markers together with reference-assisted annotation, and myeloid cells were extracted for downstream trajectory and intercellular communication analyses.

### 2.3 Identification of myeloid-cell marker genes

Marker genes were identified for distinct cell populations using Seurat with thresholds of min.pct = 0.25, |log2FC| > 0.25, and adjusted p < 0.05. These cells were annotated by integrating reference-based prediction using the SingleR package with marker gene validation against the CellMarker database and published literature (23, 24). Quantitative analysis of cellular composition, including cluster size and sample distribution, was visualized using bar plots. For deeper functional stratification, the myeloid cell cluster was isolated for subsequent analysis. Highly variable genes within each myeloid cell subset were identified using more stringent criteria (∣log_2_FC∣ > 0.5, p < 0.05). Following PCA and UMAP dimensionality reduction, marker gene expression for these myeloid cell clusters was visualized via heatmap and Cleveland dot plots. Finally, gene set variation analysis (GSVA) was performed to evaluate the enrichment of cancer hallmark pathways across the myeloid cell subsets, with results displayed in a comparative heatmap (25).

### 2.4 Analysis of intercellular communication and cell cycle states

To investigate potential interactions among cell subsets, intercellular communication was analyzed using the “iTALK” R package (v0.1.0) (26), which infers ligand-receptor interactions. This analysis was conducted both across all cells and with a specific focus on myeloid cell clusters. Communication events were classified into categories including cytokines, growth factors, immune checkpoints, and others based on the top 500 most variably expressed genes within the analyzed cell populations. The “FindLR” function was employed to identify the most robust ligand-receptor pairs, the results of which were visualized using interaction networks and dedicated LR plots. Additionally, to assess the proliferative status of cells within the identified clusters, cell cycle phase was assigned using the “CellCycleScoring” function based on established marker genes.

### 2.5 Trajectory analysis and identification of myeloid state-related genes

To delineate the developmental trajectories and distinct cellular states of myeloid cells, pseudotemporal ordering was performed using the Monocle2 package (v2.18.0) (27, 28). Monocle2 was used because it provides a mature and widely applied framework for pseudotime ordering and branch-associated gene discovery, which was suitable for identifying myeloid state-associated transcriptional programs in this study. This tool was chosen for its ability to model discrete cell fate decisions through its DDRTree algorithm, which robustly identifies branching points. Following the filtration of low-quality cells and low-dispersion genes, the top 1000 genes most informative for ordering were selected via the DifferentialGeneTest function. Dimensionality reduction and the identification of branching points in cell fate were accomplished using the DDRTree method. Once pseudotime was assigned, the expression dynamics of these ordering genes were analyzed to distinguish myeloid cells occupying different states, thereby illustrating how transcriptional divergence guides fate commitment along distinct branches. Finally, genes significantly associated with specific myeloid cell states (p < 0.05) were defined as MSRGs.

### 2.6 Identification of myeloid state-related prognostic genes

To link myeloid state transitions with clinical outcomes, MSRGs were further evaluated for prognostic relevance. We then identified genes whose expression was significantly associated with OS in STAD patients by performing Kaplan-Meier (K-M) survival analysis (p < 0.05) and univariate Cox proportional hazards regression (p < 0.01) under a stringent significance threshold (29). The resulting prognostic genes were subsequently intersected with the previously defined MSRGs. Genes were retained only if they were detectably expressed in both our single-cell and bulk RNA-seq datasets. This procedure yielded a final set of 32 genes, designated as MSRPGs. The prognostic value of these MSRPGs and their interrelationships were further explored and visualized.

### 2.7 Identification of molecular subtypes via consensus clustering

To delineate novel molecular subtypes of stomach adenocarcinoma based on the expression profiles of the defined MSRPGs, we performed unsupervised consensus clustering using the ConsensusClusterPlus R package (30). The optimal number of subtypes was selected by jointly evaluating the consensus matrix, CDF and delta-area plots, PAC values, mean cluster consensus, minimum cluster size, and biological interpretability. For k = 2-9, PAC was calculated as the proportion of off-diagonal consensus values between 0.1 and 0.9, and mean cluster consensus was calculated as the average within-cluster pairwise consensus. The resulting classification system was designated as the stomach adenocarcinoma myeloid-state classification (STAD-MSC). The expression pattern of MSRPGs across these three subtypes, along with key clinical annotations, is displayed in a consensus heatmap. Differential expression of the MSRPGs among the subtypes was further validated and visualized using boxplots.

### 2.8 PCA-based validation of the STAD-MSC subtypes

To further validate the STAD-MSC classification, principal component analysis (PCA) was performed using the expression data of patients belonging to the three identified subtypes. The principal components (PCs) derived from this analysis were visualized to illustrate subtype separation. A composite PCA score was then calculated for each patient as a continuous metric to summarize subtype-related transcriptional variation. Using the “surv_cutpoint” function, an optimal threshold was determined to dichotomize patients into high PCA-score (H-PCA) and low PCA-score (L-PCA) groups. The prognostic utility of this PCA-based stratification was subsequently evaluated through KM survival analysis.

### 2.9 Integration of multi-omics landscapes with the STAD-MSC subtypes

Multi-omics data, including tumor immune dysfunction and exclusion (TIDE) profiles and immunophenoscore (IPS) files for STAD patients, were retrieved from the TCGA and TIDE databases, respectively (31). The relative infiltration abundance of 22 immune cell types was quantified using CIBERSORT. Immune functional enrichment scores were assessed via single-sample gene set enrichment analysis (ssGSEA) (32). To characterize the tumor-immune microenvironment, enrichment scores for TIDE (including dysfunction and exclusion), Merck18, myeloid-derived suppressor cells (MDSCs), M2-polarized tumor-associated macrophages, cancer-associated fibroblasts (CAF), MSI, PD-L1 expression, and IPS incorporating CTLA-4 and PD-1 were calculated and compared across the three STAD-MSC subtypes. The association of these features with the previously defined PCA scores was also explored.

Somatic mutation profiles were analyzed with the Maftools R package to identify mutation types and visualize variant distributions. Tumor mutation burden (TMB) and MSI scores were subsequently computed (33). Waterfall plots illustrating the most frequently mutated genes were generated for the L-PCA and H-PCA subgroups, followed by a comparative analysis of TMB and MSI between these groups. The combined prognostic value of PCA score stratification and TMB was evaluated using KM survival analysis. Finally, read-depth analysis was employed to determine copy number variation (CNV) frequencies for the 32 MSRPGs. The resulting CNV landscape was mapped across chromosomes and visualized using the RCircos package.

### 2.10 Differential expression analysis between primary tumor and adjacent normal tissues

To characterize the transcriptional alterations in STAD, we performed differential expression analysis between primary tumor samples and their matched adjacent normal tissues from the TCGA-STAD cohort. Analysis was conducted using the edgeR R package (34), applying thresholds of |log_2_FC| > 1.0 and an adjusted p-value < 0.05 to identify significant differentially expressed genes (DEGs). The expression patterns of the defined MSRPGs were specifically compared between these two tissue types. Additionally, we investigated the differential activity of transcription factors and enrichment of cancer hallmark pathways between tumors and normal adjacent tissues. The results of these comparative analyses were visualized using hierarchical clustering heatmaps and volcano plots, respectively.

### 2.11 Construction of subtype-specific regulatory networks

To delineate subtype-specific regulatory architectures, we conducted pairwise correlation analyses within each STAD-MSC subtype between key MSRPGs, differentially expressed in primary versus adjacent normal tissue, and upstream transcription factors alongside downstream immune, functional, and proteomic features. Correlation pairs meeting predefined component-specific thresholds and p < 0.05 were visualized in heatmaps. The strongest subtype-specific associations were selected to construct the regulatory networks.

### 2.12 Prediction of therapeutic sensitivity across subtypes

To explore potential subtype specific pharmacological vulnerabilities, we leveraged transcriptomic data from the Cancer Cell Line Encyclopedia (CCLE) and matched pharmacogenomic profiles from the Genomics of Drug Sensitivity in Cancer (GDSC) database (35). Specifically, the expression profiles of STAD patients from each STAD-MSC subtype were projected onto the CCLE cell line expression space using a nearest centroid classifier, assigning each patient to the most transcriptionally similar cell line. The corresponding drug response data, quantified as the area under the dose–response curve (AUC) from GDSC (GDSC AUC_PUBLISHED), were then extracted as a proxy for patient level drug sensitivity, where lower AUC values indicate greater sensitivity. The distribution of predicted AUC values was compared across the three STAD MSC subtypes to prioritize compounds with nominal subtype-dependent sensitivity patterns for further exploratory investigation.

### 2.13 Analysis of chromatin accessibility via ATAC-sequencing

Assay for transposase-accessible chromatin sequencing (ATAC-seq) data for STAD samples were obtained from TCGA cohort profiling chromatin accessibility in primary human cancers (https://gdc.cancer.gov/about-data/publications/ATACseq-AWG) (36). ATAC-seq tracks provided descriptive evidence of accessible chromatin regions near selected regulatory genes in STAD. Analysis was performed using the UCSC Genome Browser (www.genome.ucsc.edu) in conjunction with the org.Hs.eg.db and TxDb.Hsapiens.UCSC.hg38.knownGene R packages for annotation. These tracks were visually inspected to characterize regional chromatin accessibility near the selected genes.

### 2.14 KEGG pathway enrichment analysis

Kyoto Encyclopedia of Genes and Genomes (KEGG) pathway enrichment analysis was performed separately for each STAD-MSC subtype to elucidate their distinct biological functions. The top ten most significantly enriched pathways per subtype were identified. Results were visualized using bubble charts, in which the size of each bubble corresponds to the count of enriched genes within a pathway, and the color gradient represents the statistical significance of the enrichment.

### 2.15 Construction and public-cohort validation of a prognostic risk-related prediction model with 5 MSRPGs

From the TCGA database, 355 STAD patients with complete transcriptomic data and metastatic status information were included in this analysis. The construction of a prognostic model proceeded in three sequential steps. First, univariate Cox proportional hazards regression was performed on the MSRPGs. Second, least absolute shrinkage and selection operator (LASSO) regression analysis was applied to optimize and select a concise gene set for the model (37). Third, patients with complete expression and survival data were randomly allocated into training and test sets at a 60:40 ratio for risk-model development and internal evaluation, resulting in 215 patients in the training set and 140 in the test set. A risk score for each patient was calculated using the formula: Risk Score = (β_1_ × expression of gene_1_) + (β_2_ × expression of gene_2_) +… + (β_n_ × expression of gene_n_), where β denotes the regression coefficient derived from the LASSO analysis (38). Based on the median risk score, patients in the training, test, and entire cohorts were stratified into high-risk and low-risk groups. The prognostic power of this stratification was assessed using KM survival analysis.

The predictive accuracy of the risk score was evaluated by generating time-dependent receiver operating characteristic (ROC) curves and calculating the corresponding area under the curve (AUC). To determine its independence from other clinical variables, univariate and multivariate Cox regression analyses were conducted incorporating the risk score, age, and TNM stage. Associations between the risk score and clinical features were further examined using Chi-square tests.

To externally validate the prognostic stratification capability of the risk score, two independent gastric cancer cohorts, GSE62254 and GSE84437, were obtained from the GEO database. The risk score for each patient in these cohorts was calculated using the same LASSO-derived formula, and patients were classified into high- and low-risk groups based on the median risk score cutoff derived from the TCGA training cohort. Kaplan-Meier survival analyses with log-rank tests were then performed to evaluate the survival differences between the two risk groups in these external datasets.

Subsequently, gene ontology (GO) and KEGG enrichment analyses, along with gene set enrichment analysis (GSEA), were performed to identify biological pathways differentially active between the two risk groups. Finally, the tumor immune microenvironment was compared by evaluating the infiltration levels of various immune cell types and TIDE profiles between high-risk and low-risk patients.

### 2.16 Protein-level clinical validation of the STAD-MSC classification in a 70-patient cohort

A retrospective cohort of 70 patients with histologically confirmed stomach adenocarcinoma who underwent surgical resection at Ruijin Hospital between 2018 and 2020 was enrolled. All patients had available paraffin-embedded tumor tissues and clinicopathological data. Clinical variables included age, sex, histological subtype, TNM stage, OS, and progression-free survival (PFS). PFS event was defined as progression or death. Among the 70 patients, 62 had available OS/PFS follow-up data and were included in the survival analyses; the remaining 8 patients had unknown OS/PFS status and were excluded from survival-related analyses. The study was approved by the institutional ethics committee, and written informed consent was obtained from all participants.

We selected NNMT, AXL, and COL1A1 as the markers for protein-level subtyping. All three genes are MSRPGs identified from our transcriptomic analyses. Their expression was significantly elevated in the HI-STAD subtype compared with the other two subtypes. They also exhibited strong co-expression with key transcription factors in the subtype-specific regulatory networks. In addition, commercial antibodies suitable for immunohistochemical detection in paraffin-embedded tissue were available for all three proteins.

Immunohistochemical (IHC) staining was performed on tissue microarray sections. Briefly, sections were deparaffinized, rehydrated, and subjected to heat-induced antigen retrieval. Endogenous peroxidase activity was blocked, followed by incubation with primary antibodies against NNMT (Abcam, ab119758, 1:100), AXL (Proteintech, 13196-1-AP, 1:1000), and COL1A1 (Servicebio, GB155707-100, 1:500). After washing, HRP-conjugated secondary antibodies were applied, and signals were visualized with DAB chromogen. Sections were counterstained with hematoxylin, dehydrated, and cover-slipped. Stained slides were digitized using a slide scanner.

Protein expression was quantified using the H-score method: H-score = 1 × percentage of weakly stained tumor cells + 2 × percentage of moderately stained tumor cells + 3 × percentage of strongly stained tumor cells, yielding a score from 0 to 300. Cutoff values for NNMT, AXL, and COL1A1 protein expression in tumor tissues were determined according to the tertile distribution of their tumor expression levels, with reference to the transcriptional expression pattern observed in the STAD molecular subtype model. Specifically, the tertile cutoff values were 145.6 and 187.6 for NNMT, 100.5 and 132.75 for AXL, and 36.6 and 64.5 for COL1A1. Each marker was categorized as low, intermediate, or high and assigned scores of 0, 1, and 2, respectively. The three scores were summed to generate a tumor protein score ranging from 0 to 6. Patients were classified as LI-STAD, MI-STAD, or HI-STAD when the total score was 0–2, 3, or 4–6, respectively.

### 2.17 Statistical analysis

Survival curves were estimated using the Kaplan-Meier method and compared with the log-rank test. Cox proportional hazards models were used to estimate hazard ratios and 95% confidence intervals. In the 70-patient protein-level cohort, Cox analyses were considered exploratory because only 62 patients had follow-up data. The proportional hazards assumption was assessed using Schoenfeld residuals. Differences in categorical variables among groups were evaluated with the Chi-square test or Fisher’s exact test. Time-dependent ROC and calibration analyses, when shown, were interpreted as exploratory because of the limited number of clinical events. All statistical analyses were conducted using R software, and a two-sided p-value < 0.05 was considered statistically significant.

## 3 Results

### 3.1 Myeloid cells identified in the tumor microenvironment

The overall study design and detailed analytical workflow are schematically depicted in **Figure 1A** and **Supplementary Figure 1**, respectively. Overall, we performed scRNA-seq-based cell annotation and trajectory inference, followed by prognostic analyses in the TCGA-STAD cohort to identify the MSRPGs. Then, we established a novel molecular classification system with three STAD subtypes (STAD-MSC), which demonstrated significant prognostic and immunological implications for STAD patients.

To identify the key cells that may have regulatory functions in tumor progression, we performed dimensionality reduction and identified 13 Seurat clusters, which were categorized into six cell types (B/plasma cells, endothelial cells, epithelial cells, fibroblasts, myeloid cells, and NK/T cells) in accordance with cell markers (**Figure 1B**). Quality-control plots for nFeature RNA and nCount RNA, together with tSNE projections stratified by sample origin, showed no dominant sample-specific separation, supporting downstream annotation and trajectory analyses. **Supplementary Figure 2A** shows all DEGs across the dataset. The expression and spatial distribution of key marker genes used to define the six cell-type populations are displayed in **Supplementary Figure 2B**. The Cleveland dot plot showed the expression levels of the four key marker genes (LYZ, CD3D, EPCAM, and DCN) across all six cell clusters (**Figure 1C**). Notably, LYZ was highly expressed in myeloid cells and detectable in a subset of epithelial cells, whereas DCN expression was enriched in fibroblasts. The cellular composition across individual samples is presented in bar plots, with **Supplementary Figure 3A** summarizing the cell numbers and proportions derived from the 40 samples. Furthermore, a heatmap showed the top five marker genes for each cell type (**Figure 1D**). Cellular communication analysis showed extensive interactions between myeloid cells and other cell populations in the TME (**Figure 1E**). Cell-cycle scoring indicated broadly comparable proportions of S and G2/M phase cells across major cell types, suggesting clustering was not primarily driven by proliferative status (**Supplementary Figure 3C**).

**Figure 2:**
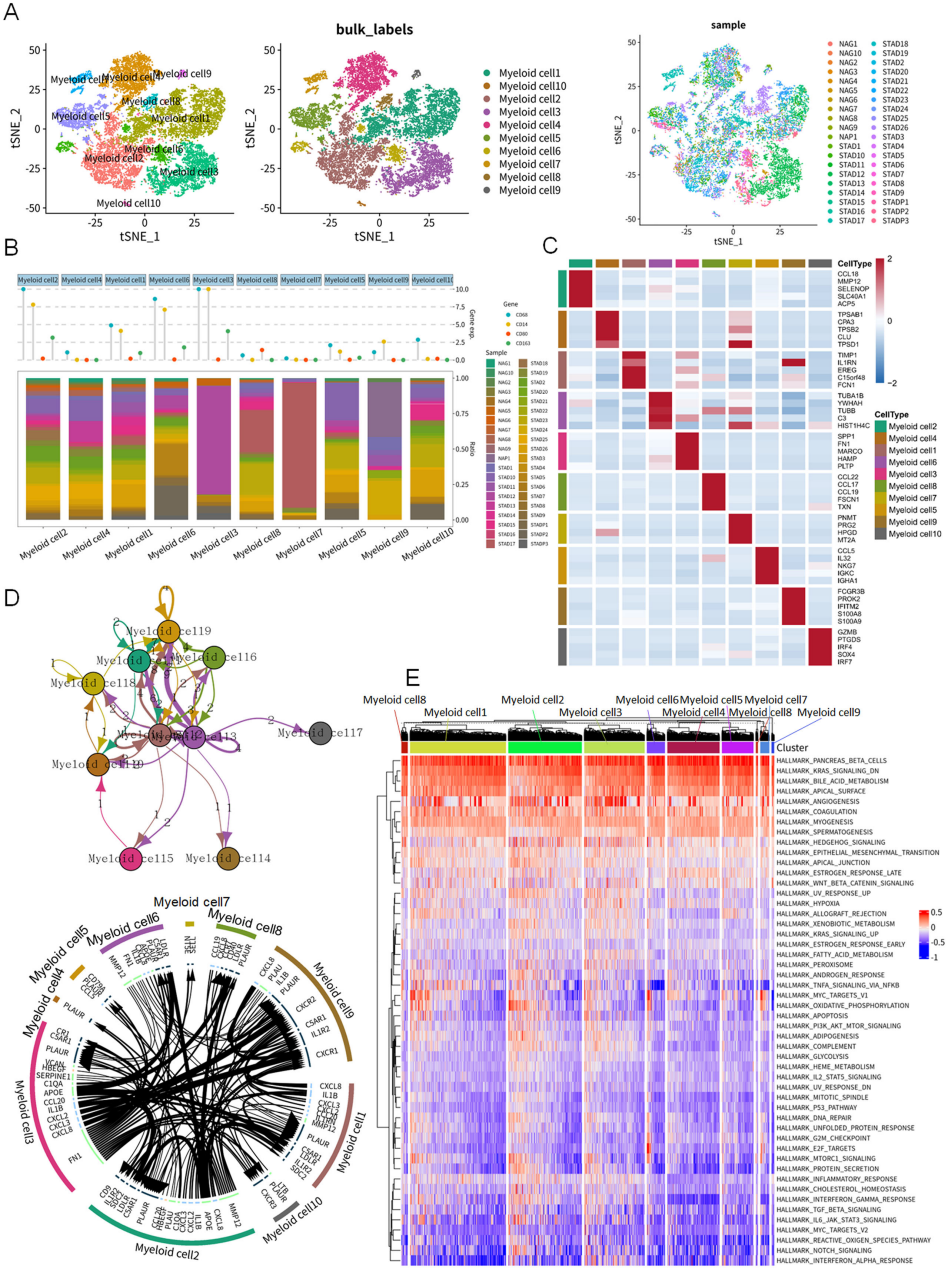
Identification and characterization of myeloid subtypes in STAD microenvironment. **(A)** The tSNE plots demonstrated the 10 myeloid cell clusters, with the marker genes specifically pointed out. **(B)** The Cleveland dot plot illustrated the average expression levels of four marker genes (CD68, CD14, CD80, CD163) in the 10 myeloid cell clusters. **(C)** The heatmap revealed the differential expression of the top five DEGs of the 10 myeloid cell subtypes. **(D)** The cellular communication network and the corresponding ligand-receptor plot revealed that strong and complex interactions existed among the 10 myeloid cell clusters. **(E)** GSVA identified the activated hallmark signaling pathways, with selected hallmark pathways, including KRAS signaling-related, metabolic, and apical-surface programs, showed variable activity across myeloid subsets. STAD, stomach adenocarcinoma; DEGs, differentially expressed genes; GSVA, gene set variation analysis.

Myeloid cells play a crucial role in shaping the TIME. To better elucidate their biological functions in STAD, we extracted the myeloid cell clusters for further analysis and annotated 10 distinct myeloid subsets (**Figure 2A**). **Figure 2B** shows the expression levels of the four key marker genes (CD68, CD14, CD80, and CD163) that characterized these subsets. The Cleveland dot plot revealed a distinct expression pattern. CD68 is highly expressed, and CD14 exhibits a comparable trend across various myeloid cells. In contrast, CD80 expression was enriched in the myeloid 8 subset. As shown in **Figure 2C**, a heatmap of the top five key genes for each myeloid cluster displayed extensive heterogeneity. Cellular communication analysis revealed strong and complex interactions among the ten myeloid cell subsets (**Figure 2D**). Considering the biological functions, GSVA identified the activated cancer hallmarks (**Figure 2E**). Notably, GSVA highlighted the recurrent enrichment of KRAS signaling–related and metabolic gene sets across myeloid subsets. Myeloid cells engaged in extensive interactions with other populations, indicating a central role in the ecosystem and justifying their selection for further investigation.

### 3.2 Trajectory analysis delineated transcriptional dynamics and myeloid cell states

**Figure 3:**
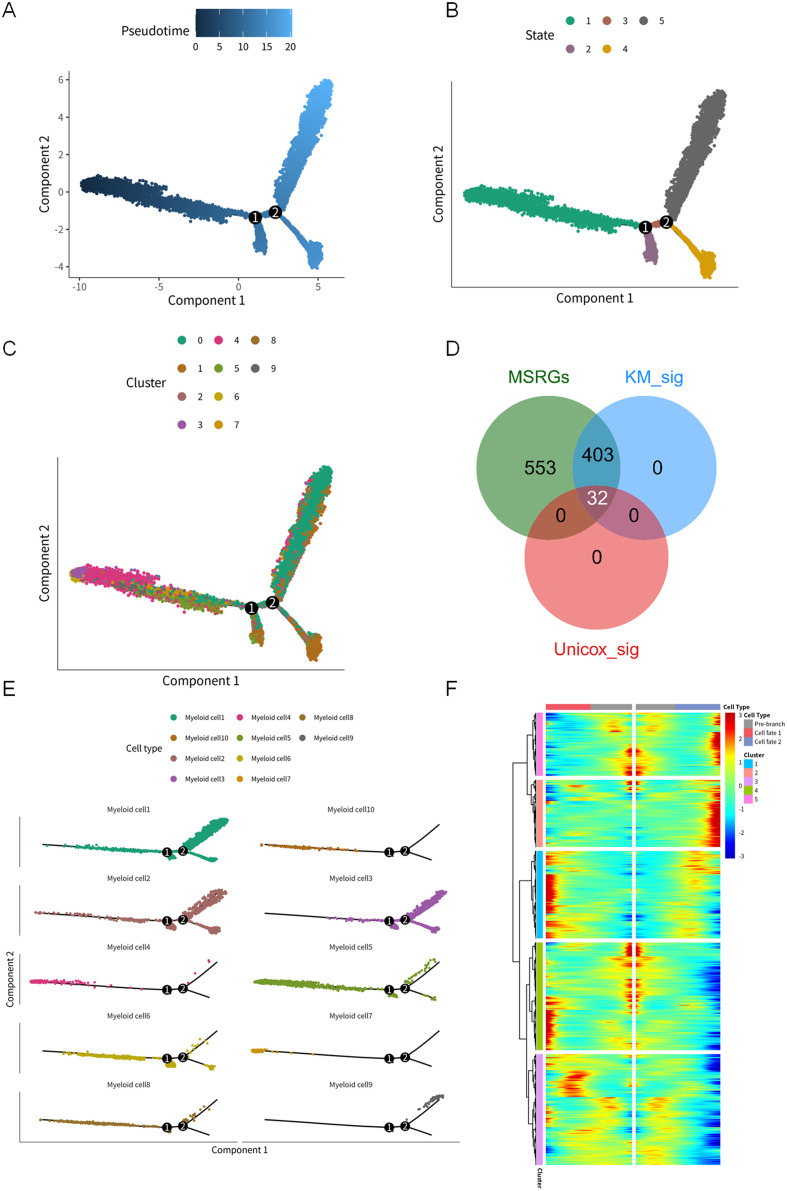
The pseudotime analysis of myeloid cells. **(A)** The differentiation trajectory of myeloid cells, in which the color gradient represents pseudotime. **(B)** The 5 differentiation states (state 1-5) distinguished from the differentiation trajectory. **(C)** The overview map of the 10 myeloid subtypes’distribution in the differentiation trajectory. **(D)** Intersecting MSRGs with genes significantly associated with OS in Kaplan-Meier and univariate Cox analyses identified 32 MSRPGs. **(E)** Monocle2 pseudotime analysis showed the distribution of 10 myeloid cell clusters in the differentiation trajectories with two bifurcation points. **(F)** The heatmap depicted the BEAM analysis, which demonstrated the potential association between the differential expression of myeloid cell state-related genes and five myeloid cell states. MSRG, myeloid state-related genes; MSRPGs, myeloid state-related prognostic genes; BEAM, branched expression analysis modeling.

To decipher the heterogeneity, differentiation dynamics, and functional states of myeloid cells within the TME, Monocle 2 was applied to infer pseudotemporal trajectories. In the pseudotime trajectory depicted in **Figure 3A**, a color gradient represents the progression of cellular differentiation. With two bifurcation points, five differentiation states were captured, of which state 1 was the earliest stage, whereas trajectory states 4 and 5 were situated at the terminal branch (**Figure 3B**). The ten myeloid clusters identified by Monocle were positioned along the inferred developmental trajectory (**Figure 3C**). Our analysis identified differentially expressed genes across the five states. A heatmap visualizing their expression patterns is presented in **Figure 3F**, highlighting their pivotal roles in defining the myeloid cell states. Accordingly, we designated these genes as MSRGs, constituting a candidate biomarker panel for defining the myeloid cellular states.

### 3.3 Determination of the myeloid state-related prognostic genes

To evaluate the clinical significance of myeloid cell-associated genes, we conducted a comprehensive prognostic analysis within the TCGA cohort, whose baseline information is shown in **Supplementary Table 1**. Initial screening using KM analysis revealed that 435 genes were significantly linked to overall survival (KM_sig, p < 0.05). Subsequent univariate Cox proportional hazards regression analysis refined this list, identifying 32 genes with prognostic value (UniCox_sig, p < 0.01). The overlap between these prognostic genes and the core myeloid cell gene set defined a 32-gene signature, designated as MSRPGs (**Figure 3D**). The complete list of the 32 MSRPGs is provided in **Supplementary Table 2**. To clarify the relationship between the 32 MSRPGs and the Monocle-defined myeloid states, we annotated each MSRPG with its corresponding trajectory state in **Supplementary Table 2**. The state-level distribution suggested functional heterogeneity among the MSRPGs. State 1 contained genes related to lipid handling, efferocytosis, receptor signaling, and adaptive survival. State 2 was enriched for inflammatory monocyte-like and matrix/vascular interaction genes. State 3 was dominated by extracellular matrix remodeling, collagen organization, EMT/stromal interaction, and CXCR4-linked trafficking genes. State 4 contained regulatory and metabolic adaptation markers, including NNMT, whereas State 5 was represented by NT5E, suggesting adenosine-related immune regulation. Detailed survival correlations for the MSRPGs are shown in **Supplementary Figure 4A**, and a co-expression network illustrating the interplay among these genes is presented in **Supplementary Figure 4B**. Notably, all MSRPGs derived from the five Monocle-defined states (STAD1-STAD5) were consistently identified as risk factors.

### 3.4 STAD-MSC and its immunological implications

**Figure 4:**
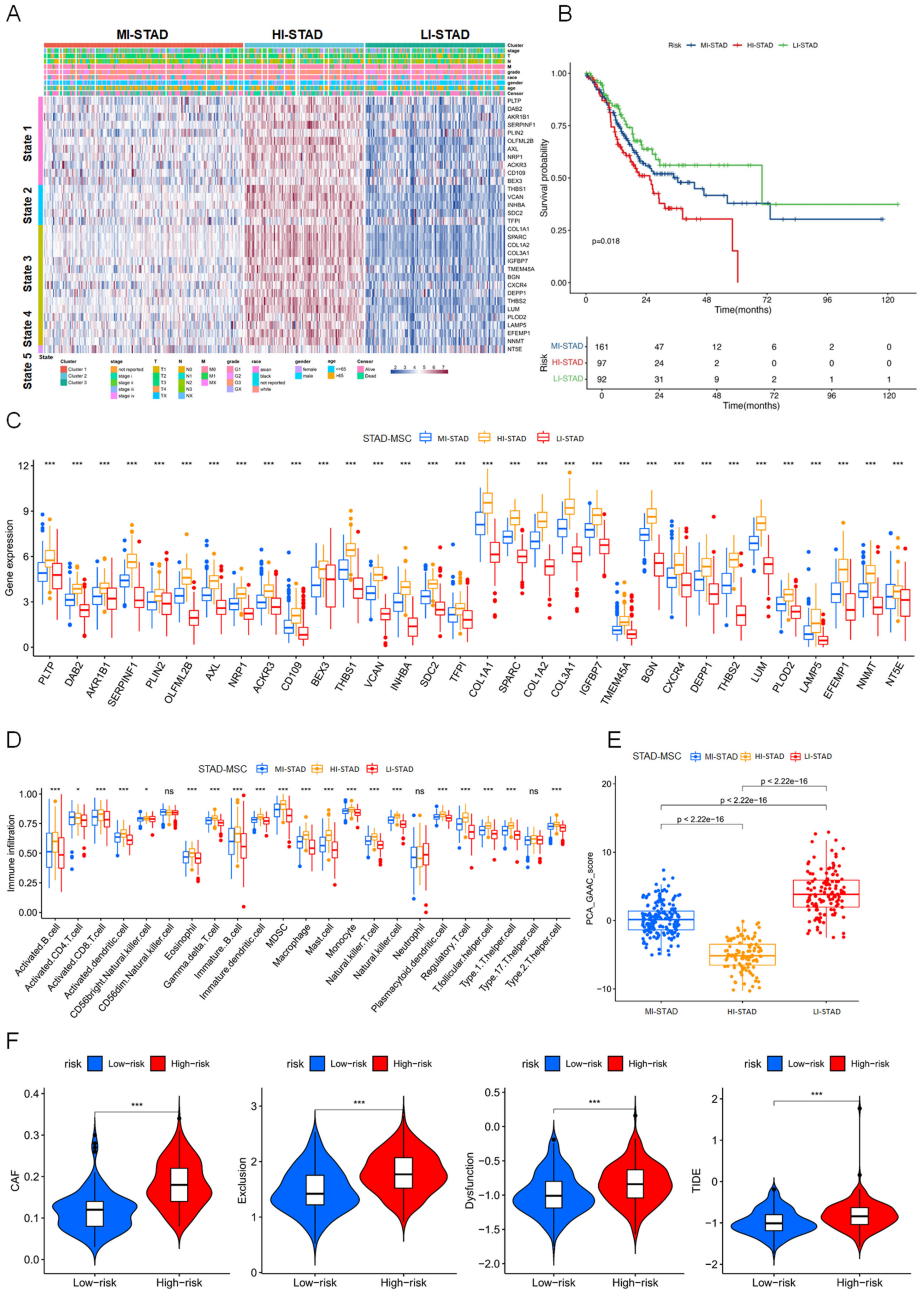
Construction of myeloid state-related classification of STAD patients in TCGA cohort. **(A)** The heatmap integrated the clinical information, disease conditions, expression levels of MSRPGs, trajectory states, and three STAD subtypes. **(B)** In the KM survival plot, LI-STAD and HI-STAD showed the highest and lowest survival probabilities, respectively (global log-rank p = 0.018), indicating the prognostic relevance of STAD-MSC. Cox proportional hazards analysis was performed using LI-STAD as the reference group. Compared with LI-STAD, HI-STAD showed an increased risk of death [HR = 1.88, 95% CI = 1.19-2.96], whereas MI-STAD showed an intermediate risk [HR = 1.33, 95% CI = 0.86-2.05]. **(C)** MSRPG expression levels differed significantly among the three groups. Notably, cluster 2 (HI-STAD) and cluster 3 (LI-STAD) respectively had the highest and the lowest expression of genes in state 1 and 3, which were all risk factors. **(D)** The immune infiltration of most immune cells was highest in cluster 2, especially eosinophil, regulatory T cells, MDSCs, macrophages, and NK cells. **(E)** PCA scores were highest in LI-STAD and lowest in HI-STAD, with MI-STAD showing intermediate scores. **(F)** In the violin plots, CAF, dysfunction, exclusion, and the composite TIDE scores were elevated in the higher-risk group compared to the lower-risk groups. STAD, stomach adenocarcinoma; TCGA, The Cancer Genome Atlas; MSRPGs, myeloid state-related prognostic genes; KM, Kaplan-Meier; LI-STAD, low immune infiltration STAD; HI-STAD, high immune infiltration STAD; MI-STAD, moderate immune infiltration STAD; STAD-MSC, stomach adenocarcinoma myeloid-state classification; MDSCs, myeloid-derived suppressor cells; PCA, principal component analysis; CAF, cancer-associated fibroblast; TIDE, tumor immune dysfunction and exclusion. ‘***’ indicates p < 0.001. For completeness, ‘*’ indicates p < 0.05 and ‘ns’ indicates not significant.

To establish a clinically applicable prognostic model, we performed consensus clustering on STAD samples from the TCGA database using the expression profiles of the MSRPGs. This analysis defined three molecular subtypes, which we refer to as the STAD-MSC. We evaluated alternative cluster numbers (k = 2 to 9) and selected k = 3 as the most balanced solution, considering cluster stability, group separability, and biological interpretability (**Supplementary Figure 5**). Although quantitative stability metrics (**Supplementary Table 3**) showed that k = 2 achieved the highest numerical stability, it yielded only a binary partition that did not align with our biological framework; k = 3 still maintained a low PAC of 0.175, a high mean cluster consensus, and a minimum cluster size of 98, supporting a stable three-group solution while avoiding the overly broad two-cluster solution. The consensus matrix, CDF curve, and delta area plot confirmed the stability of this three-cluster solution (**Supplementary Figures 6A–C**). Subsequently, an integrative heatmap (**Figure 4A**) was generated to visualize the alignment between the three STAD-MSC subtypes and key variables, including clinical parameters, MSRPG expression, and previously identified trajectory states. Survival analysis revealed significant prognostic stratification by STAD-MSC (global log-rank p = 0.018), with patients in subtype 2 exhibiting the most unfavorable overall survival (**Figure 4B**). The differential expression of risk-associated MSRPGs, which was highest in subtype 2 and lowest in subtype 3, provides a potential molecular basis for the observed survival disparity (**Figure 4C**). Analysis of the tumor immune microenvironment revealed pronounced heterogeneity across the subtypes. Subtype 2 was characterized by broad and intense immune infiltration, encompassing eosinophils, regulatory T cells, MDSCs, macrophages, and NK cells (**Figure 4D**). Reflecting this immune landscape, the subtypes were subsequently classified into low immune infiltration STAD (LI-STAD), moderate immune infiltration STAD (MI-STAD), and high immune infiltration STAD (HI-STAD) immune infiltration groups. Importantly, the terms LI-STAD, MI-STAD, and HI-STAD refer to the relative abundance of immune infiltration rather than favorable or unfavorable immune activity. Thus, while HI-STAD indicates the highest level of immune cell infiltration, this subtype paradoxically exhibited the poorest prognosis, as the infiltrating immune microenvironment was accompanied by T-cell dysfunction, T-cell exclusion, CAF enrichment, elevated immune checkpoint expression, and myeloid-rich suppressive features. To further summarize subtype-related transcriptional variation, we performed principal component analysis (PCA) on the three subtypes. PCA clearly distinguished all three subtypes (LI-STAD, MI-STAD, and HI-STAD) in the reduced dimension space (**Supplementary Figure 6D**). The PCA scores followed an order inverse to the immune infiltration levels, with patients in the LI-STAD group displaying the highest scores, those in the HI-STAD group displaying the lowest scores, and those in the MI-STAD group occupying an intermediate position (**Figure 4E**). Consistently, a lower PCA score was significantly associated with shorter OS (p < 0.001; **Supplementary Figure 6E**) and strongly correlated with increased infiltration of multiple immune cell types (**Supplementary Figure 6F**). To define the immunological basis of the poor prognosis high-risk group (characterized by low PCA score/HI-STAD subtype), we profiled the tumor immune microenvironment using the TIDE algorithm. This group displayed significantly elevated scores for CAF, T-cell Dysfunction, T-cell Exclusion, and the composite TIDE score relative to the lower-risk groups. Collectively, these data indicate that the high immune infiltration in HI-STAD patients is functionally polarized toward an immunosuppressive and exclusionary microenvironment, which is consistent with their unfavorable clinical outcomes (**Figure 4F**). In summary, leveraging MSRPGs, we developed STAD-MSC, a novel molecular classification system that effectively categorizes STAD patients into subgroups with distinct survival outcomes and immune microenvironment characteristics.

### 3.5 Multi-omics analysis of genomics and epigenomics

To explore genomic and epigenomic landscapes across the prognostic subtypes and to identify potential driver alterations and multi-omics biomarkers, we comprehensively characterized these landscapes in STAD. The top 20 mutated MSRPGs in the H-PCA and L-PCA groups are displayed in **Supplementary Figures 7A** and **S6B**, respectively. The mutation rate was approximately 50% in the H-PCA group and 25% in the L-PCA group, indicating differential genomic instability between the subtypes. VCAN mutations were prevalent in both groups, followed by those in COL1A2. Mutations were also observed in COL1A1, AXL, and LUM genes. Both the TMB and MSI scores demonstrated a linear correlation with the PCA score (**Supplementary Figure 7C**). Notably, KM analysis revealed that patients with high TMB (H-TMB) had significantly better OS (**Supplementary Figure 7D**). Consistently, among all STAD patients, the combination of H-TMB and H-PCA was associated with the most favorable OS (p < 0.001, **Supplementary Figure 7E**). CNV analysis revealed frequent alterations in all MSRPGs. These genes were distributed across multiple chromosomes, with notable clusters on chromosomes 2, 3, 5, 6, and 7 (**Supplementary Figure 7F**). We further interrogated whether somatic copy-number alterations could directly account for the observed mRNA overexpression of MSRPGs by correlating gene-level CNV status with matched transcriptomic profiles in the TCGA-STAD cohort. The overall CNV-expression associations for the MSRPG panel were generally weak to modest. For instance, COL1A1 copy-number status showed no clear association with mRNA abundance (rho = 0.04, p = 0.442), indicating that CNV alone is unlikely to explain COL1A1 upregulation (**Supplementary Figure 7G**). Collectively, these findings suggest that CNVs alone are unlikely to be the primary drivers of MSRPG upregulation. Instead, the subtype-specific expression patterns likely emerge from a multifaceted regulatory interplay involving transcriptional, epigenomic, and microenvironmental influences. Significant differences were observed between the subtypes in terms of mutation rates, TMB, MSI, and CNV. Patients with a high TMB combined with a high PCA score exhibited the most favorable OS, providing a multi-omics basis for precise stratification.

### 3.6 Differential analysis for genes, TFs, and cancer hallmarks between STAD tumor and adjacent normal tissues

To comprehensively identify tumor-specific molecular alterations in STAD and validate the role of MSRPGs in tumorigenesis, we performed a comprehensive differential analysis between tumor tissues and matched adjacent normal samples. The DEGs between tumor and normal tissues were displayed in **Supplementary Figures 8A, B**. All MSRPGs were validated as significant DEGs and were explicitly annotated in the volcano plot. Subsequently, the differentially expressed transcription factors (DETFs) were identified and their expression patterns are visualized in **Supplementary Figures 8C, D**. To assess the functional implications of these transcriptional changes, GSVA was employed to score the activity of the cancer hallmark pathways (**Supplementary Figure 8E**). The quantified GSVA scores for each hallmark are ranked in **Supplementary Figure 8F**, with the most significantly activated and suppressed pathways displayed at the top and bottom, respectively. Among the most significantly activated hallmarks were MYC_TARGETS_V2, E2F_TARGETS, G2M_CHECKPOINT, and MTORC1_SIGNALING. Conversely, KRAS_SIGNALING_DN, BILE_ACID_METABOLISM, MYOGENESIS, and PANCREAS_BETA_CELLS were markedly suppressed in tumor tissues. All MSRPGs were confirmed to be significantly differentially expressed between tumor and normal tissues. Tumor tissues exhibited significant activation of proliferation-related pathways and suppression of metabolic pathways.

### 3.7 Construction of the regulatory networks for three STAD subtypes

**Figure 5:**
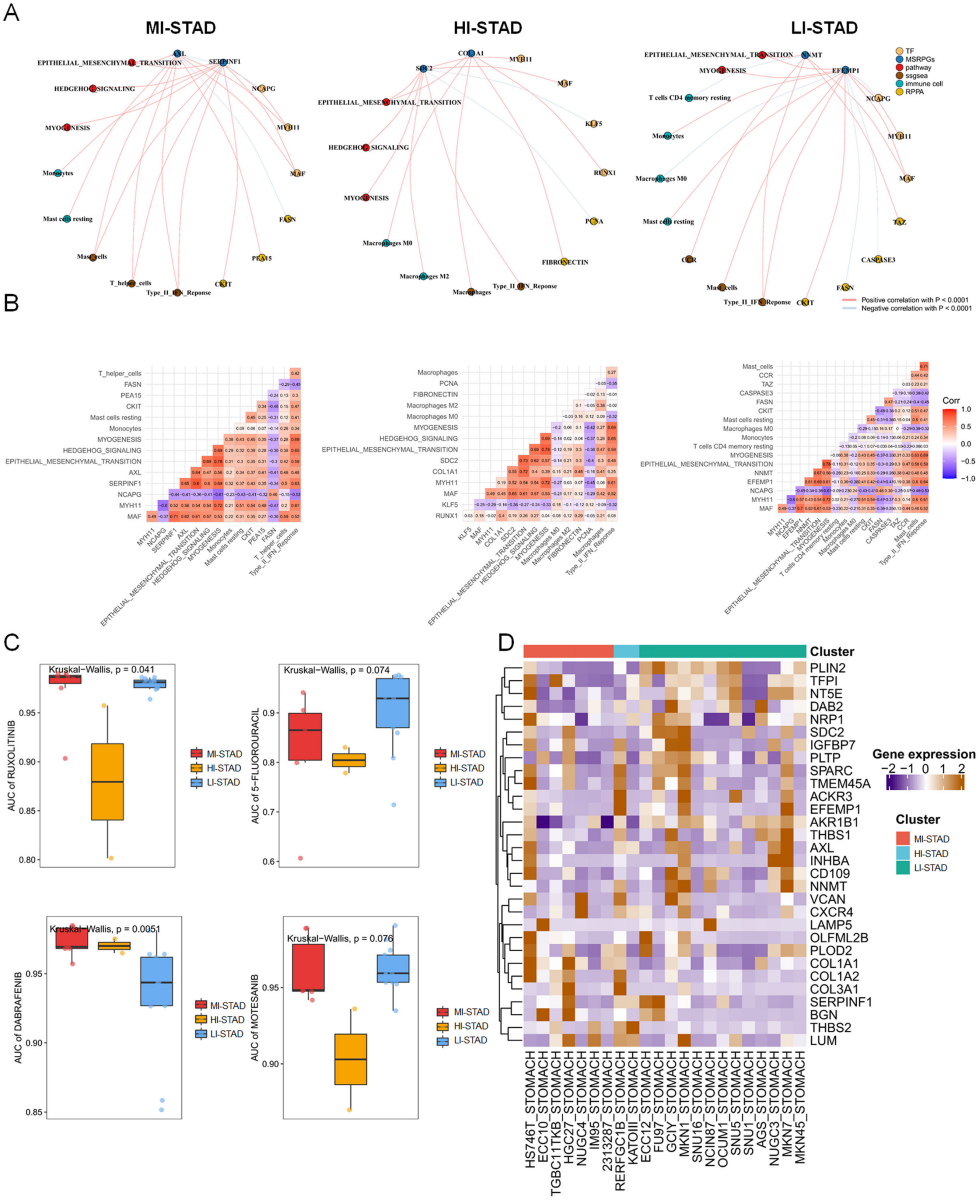
The regulatory networks and predicted drug-response patterns across STAD subtypes. **(A)** The regulatory networks were built by correlation analysis, which contained the midstream MSRPGs and critical biological components (upstream TFs and downstream factors including pathway, ssGSEA, immune cell and RPPA). **(B)** The correlation coefficients between all the key biological components were shown. In MI-STAD, the dominant regulatory MSRPGs were AXL and SERPINF1, which both exhibited significant co-expression patterns with TFs (MYH11, MAF), hallmark pathway (epithelial mesenchymal transition), and ssGSEA signature (Type II IFN Response). In HI-STAD, the most aggressive subtype, regulatory MSRPGs were SDC2 and COL1A1, both of which demonstrated significant co-expression with the epithelial mesenchymal transition pathway. In LI-STAD, NNMT and EFEMP1 dominated. EFEMP1 also exhibited high correlation coefficients with the TF MAF (R = 0.70) and the Type II IFN Response signature (R = 0.64). **(C)** Predicted drug-response metrics were compared across the three subtypes, and exploratory subtype-associated differences are shown. **(D)** After matching subtype-specific expression profiles to CCLE cell-line expression profiles, the MSRPG-based results are shown. STAD, stomach adenocarcinoma; MSRPGs, myeloid state-related prognostic genes; TF, transcription factor; LI-STAD, low immune infiltration STAD; HI-STAD, high immune infiltration STAD; MI-STAD, moderate immune infiltration STAD; ssGSEA, single-sample gene set enrichment analysis.

To elucidate the distinct molecular mechanisms associated with each STAD immune subtype, we constructed regulatory networks based on correlation analysis. These networks integrated the core MSRPGs with key upstream regulators, such as TFs and downstream effectors, encompassing pathway activities, ssGSEA signatures, immune cell infiltration, and proteomic (RPPA) profiles (**Figure 5A**). The components of these subtype-specific networks may be associated with the characteristic phenotypes of the three STAD subtypes. A comprehensive matrix of the correlation coefficients among all key biological components is shown in **Figure 5B**. In the MI-STAD subtype, the dominant regulatory MSRPGs were AXL and SERPINF1. Both exhibited significant co-expression patterns with upstream TFs (MYH11, MAF), the activated hallmark pathway (Epithelial Mesenchymal Transition), and the ssGSEA signature (Type II IFN Response). In the more aggressive HI-STAD subtype, the regulatory MSRPGs were SDC2 and COL1A1, both of which demonstrated significant co-expression with the Epithelial Mesenchymal Transition pathway. In the LI-STAD subtype, NNMT and EFEMP1 were the key regulatory MSRPGs. Both showed significant co-expression with the Epithelial Mesenchymal Transition pathway. Notably, EFEMP1 exhibited high correlation coefficients with the TF MAF (R = 0.70) and Type II IFN Response signature (R = 0.64). The expression patterns of the key transcription factors and MSRPGs across these three subtype-specific regulatory networks are illustrated in the heatmap (**Supplementary Figure 9A**). Furthermore, pathway enrichment analysis highlighted the ten most significantly associated pathways for each STAD subtype (**Supplementary Figure 9B**). To complement these network-based observations with epigenomic evidence, we performed ATAC-seq analysis to assess chromatin accessibility at regulatory loci of key MSRPGs. Accessible chromatin signals were detected around pivotal subtype-associated genes, including AXL, COL1A1, NNMT, CXCR4, SDC2, and SERPINF1 indicating a regulatory-permissive configuration at these sites that is broadly consistent with their transcriptional activity within the subtype-specific networks (**Supplementary Figure 9C**). These data, however, were interpreted as supportive rather than causal evidence for transcriptional regulation, as open chromatin alone does not necessarily dictate active transcription. Subtype-specific regulatory networks were constructed, centered on key genes. These networks revealed co-expression patterns with upstream transcription factors and downstream pathways, which were supported by epigenomic data on chromatin accessibility.

### 3.8 Exploration of subtype-associated drug sensitivity

To identify potential subtype specific pharmacological vulnerabilities, we performed in silico drug sensitivity profiling by matching the transcriptomic profiles of the three STAD−MSC subtypes to the CCLE cell line panel, followed by extraction of the corresponding GDSC AUC_PUBLISHED values. Among the evaluated agents, dabrafenib exhibited the most pronounced between−subtype difference, with the HI−STAD subtype showing markedly lower AUC values, which indicating greater predicted sensitivity, compared with the other two subtypes. This association was significant at the nominal level (p = 0.0051). Ruxolitinib also showed a nominal association (p = 0.041). In contrast, the remaining two agents, 5−fluorouracil and motesanib, did not show evidence of subtype−dependent effects at the nominal level (p = 0.074 and 0.076, respectively). The subtype−specific AUC distributions for these compounds are presented in **Figures 5C**, **D**. These exploratory observations suggest potential trends that may warrant further preclinical investigation.

### 3.9 Construction and validation of a prognostic prediction model with MSRPGs

**Figure 6:**
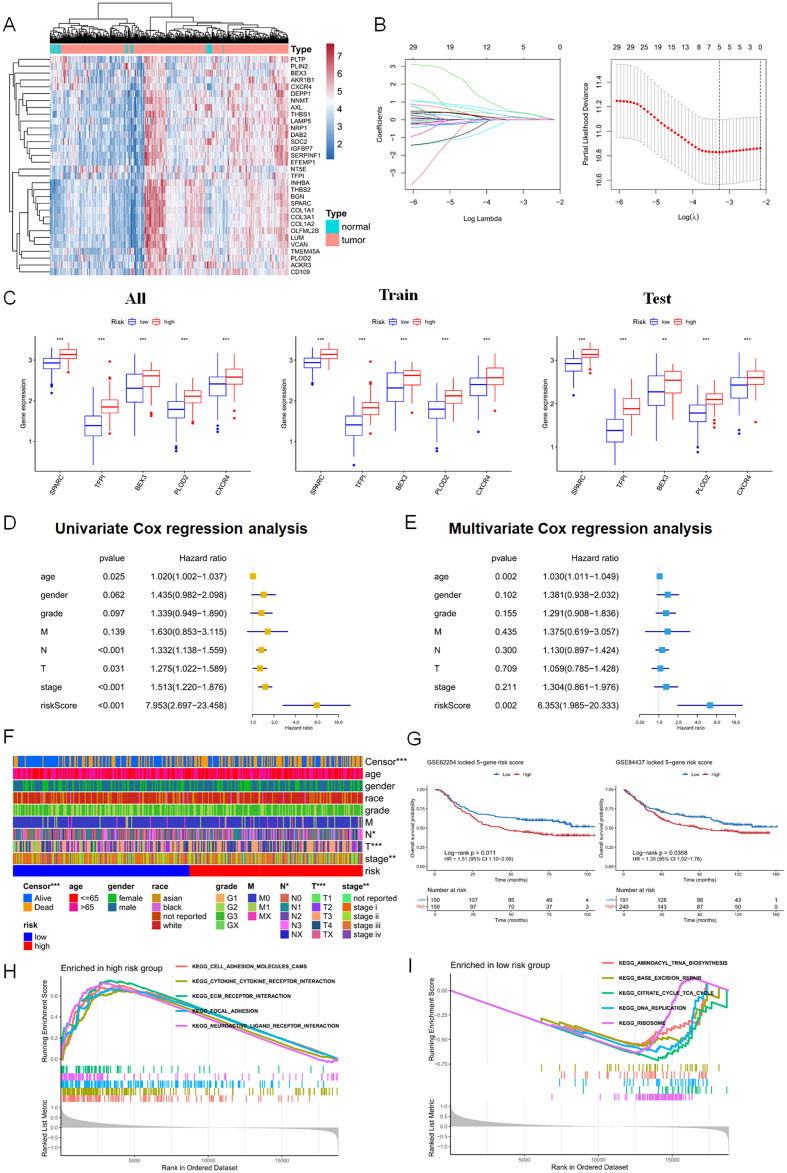
Construction and validation of a prognostic prediction model. **(A)** The heatmap demonstrated the expression level of MSRPGs between STAD tumor tissues and matched adjacent normal samples. **(B)** LASSO regression was used to reduce the 32 MSRPGs to a 5-gene risk signature. **(C)** The expression level of the selected genes was compared between the low-risk and high-risk patients in the entire, training, and test sets, respectively; all showed statistically significant differences. **(D)** Through univariate Cox regression analysis, the risk score was identified as a significant factor (HR = 7.953, 95% CI = 2.697-23.458, p < 0.001). **(E)** Through multivariate Cox regression analysis, the risk score was identified as an independent prognostic factor (HR = 6.353, 95% CI = 1.985–20.333, p < 0.001). **(F)** The Chi-square test demonstrated that patients in the high-risk group were significantly associated with a higher probability of death, higher grade, T stage, M stage, and worse cancer status. **(G)** KM curves for overall survival in the GSE62254 and GSE84437 cohorts stratified by the five-gene risk score. The high-risk group had significantly worse survival in both cohorts (GSE62254: HR = 1.51, P = 0.0116; GSE84437: HR = 1.35, P = 0.0365). **(H)** The five most significantly enriched KEGG pathways for high-risk patients were presented, prominently featuring a coordinated network of cell adhesion molecules for immune cell interaction, cytokine signaling for inflammation, ECM-receptor engagement for fibrotic stroma, focal adhesion for cellular mechanics, and neuroactive ligand-receptor activity. **(I)** The five most significantly enriched KEGG pathways for low-risk patients were presented, prominently featuring genetic information fidelity, core metabolism, and protein synthesis. MSRPGs, myeloid state-related prognostic genes; STAD, stomach adenocarcinoma; LASSO, least absolute shrinkage and selection operator; KEGG, Kyoto Encyclopedia of Genes and Genomes.

To transform the MSRPGs into a quantitative risk score model for individualized prognostic assessment and to evaluate its prognostic performance and public-cohort reproducibility, we constructed a prognostic prediction model based on the MSRPGs. First, we confirmed the differential expression of these MSRPGs between STAD tumor tissues and matched adjacent normal samples (**Figure 6A**). ORA revealed that the MSRPGs were significantly enriched in pathways related to extracellular matrix organization, epithelial-mesenchymal transition (EMT), and specific invasive cancer signatures, implicating their role in tumor-stroma interactions and aggressive progression (**Supplementary Figure 10A**). The hazard ratios (HR) of individual MSRPGs from univariate Cox regression analysis are presented in **Supplementary Figure 10B**. To prevent model overfitting and identify the most robust prognostic signature, we applied LASSO regression analysis (**Figure 6B**). This process refined the gene set to a parsimonious signature of five genes, namely SPARC, TFPI, BEX3, PLOD2, and CXCR4, whose coefficients were determined for the final model. The risk score was calculated as follows: risk score = 0.455 × SPARC + 0.422 × TFPI + 0.183 × BEX3 + 0.106 × PLOD2 + 0.161 × CXCR4. The five genes were therefore selected by penalized regression rather than manual prioritization. Subsequently, STAD patients were randomly divided into training and testing cohorts. The expression patterns of the 5-gene signature across all samples are visualized in a heatmap (**Supplementary Figure 10C**). Significant differences in the expression of this signature were confirmed between the predicted low- and high-risk groups in both cohorts (**Figure 6C**). A risk score was calculated for each patient based on the 5-gene signature. The distribution of risk scores against OS status is shown in the scatter plots (**Supplementary Figure 10D**). As expected, higher risk scores were strongly associated with significantly shorter OS, as validated by the KM survival analysis (**Supplementary Figure 10E**). ROC curves and AUC values suggested moderate prognostic discrimination of the model (**Supplementary Figure 10F**). In the public 355-patient validation cohort, univariate and multivariate Cox analyses identified the risk score as a significant prognostic factor (**Figure 6D**, HR = 7.953, 95% CI = 2.697-23.458, p < 0.001) and an independent prognostic factor (**Figure 6E**, HR = 6.353, 95% CI = 1.985–20.333, p < 0.001), respectively. Furthermore, patients in the high-risk group were associated with significantly worse clinical outcomes. **Figure 6F** demonstrates their higher tumor grade and advanced stage. To further validate the prognostic performance of the five-gene risk score in additional independent datasets, we applied the model to the GSE62254 and GSE84437 cohorts. Kaplan-Meier survival analysis showed that patients stratified into the high-risk group experienced significantly worse overall survival in both cohorts (GSE62254: HR = 1.51, P = 0.0116; GSE84437: HR = 1.35, P = 0.0365; **Figure 6G**), consistently supporting the robust predictive capacity of the signature. Results for other individual genes are shown in **Supplementary Figure 11A**.

The high-risk group was characterized by the enrichment of pathways central to cell communication and microenvironment interaction which collectively underpin enhanced immune cell recruitment, inflammatory signaling, and metastatic potential (**Figure 6H**). In contrast, the low-risk group signature was predominantly linked to core metabolic and genetic information processing pathways, reflecting a dependency on fundamental biosynthetic and proliferative machinery (**Figure 6I**). GO and Hallmark pathway enrichment results for the two groups are shown in **Supplementary Figure 10G**. The model effectively stratified patients into high- and low-risk groups, and the risk score was an independent prognostic factor. The high-risk signature was enriched for pathways involved in microenvironment interaction and metastasis.

### 3.10 Association between immune microenvironment characteristics and risk stratification in STAD

To systematically compare the immune microenvironment composition and functional state between the risk groups and evaluate the model’s potential for predicting immunotherapy response, we performed a comprehensive immune subtype analysis. Analysis of immune subtypes revealed that both low- and high-risk STAD patients were significantly associated with the C2 and C1 subtypes (p = 0.014, **Supplementary Figure 12A**). We subsequently quantified the proportions of 22 tumor-infiltrating immune cell types (**Supplementary Figure 12B**) and compared both immune cell infiltration and immune function scores between the low- and high-risk groups (**Supplementary Figures 12C, D**). Significant differences in these immune landscapes were observed between the risk groups, and these differences were further linked to distinct clinical outcomes (**Supplementary Figures 11E, F**). Collectively, the high- and low-risk groups displayed dichotomous immune landscapes. The high-risk group was characterized by features of immune exclusion and dysfunction, whereas the low-risk group showed higher MSI and distinct immune infiltration patterns. These results support an association between immune microenvironment composition and clinical risk stratification in STAD.

### 3.11 Protein-level clinical validation of the STAD-MSC classification using a three-protein classifier

A total of 70 patients were enrolled in the retrospective cohort, and their detailed information is shown in **Table 1**. Among the 70 patients, 11 had died, 16 had experienced progression, and 8 lacked OS/PFS follow-up data. Female patients accounted for 21.43% and male patients for 78.57%. 65.71% of patients were diagnosed with adenocarcinoma, 17.14% with signet ring cell carcinoma, and 11.43% with mucinous adenocarcinoma. In addition, 47.14% and 27.14% of patients were in T4 and T3 disease respectively, while only 34.29% of patients had no lymph node metastasis and 1.43% of patients had distant metastasis.

**Table 1:** Clinicopathological characteristics of the retrospective Ruijin Hospital STAD cohort (n = 70).

Variables	Number (%)	Mean ± SD; Median (range)
NNMT_T expression		167.17 ± 51.84; 171.00 (55.40-285.00)
AXL_T expression		119.99 ± 44.19; 113.00 (34.50-256.50)
COL1A1_T expression		53.83 ± 35.09; 43.95 (6.90-189.00)
NNMT_N expression		96.13 ± 34.42; 95.30 (32.80-185.40)
AXL_N expression		93.91 ± 44.88; 91.50 (35.25-299.50)
COL1A1_N expression		78.38 ± 24.23; 76.05 (24.00-137.40)
Age		69.30 ± 9.21; 70.00 (42.00-89.00)
OS days		1846.32 ± 569.63; 2089.50 (370.00-2550.00)
PFS days		1701.67 ± 693.54; 2065.00 (189.00-2550.00)
Tumor total score		
0	4 (5.71)	
1	10 (14.29)	
2	17 (24.29)	
3	14 (20.00)	
4	9 (12.86)	
5	11 (15.71)	
6	5 (7.14)	
NNMT_T score		
low	24 (34.29)	
middle	23 (32.86)	
high	23 (32.86)	
AXL_T score		
low	24 (34.29)	
middle	23 (32.86)	
high	23 (32.86)	
COL1A1_T score		
low	24 (34.29)	
middle	23 (32.86)	
high	23 (32.86)	
NNMT_N score		
low	65 (92.86)	
middle	5 (7.14)	
AXL_N score		
low	42 (60.00)	
middle	15 (21.43)	
high	13 (18.57)	
COL1A1_N score		
low	3 (4.29)	
middle	18 (25.71)	
high	49 (70.00)	
Age group		
	33 (47.14)	
	37 (52.86)	
Sex		
female	15 (21.43)	
male	55 (78.57)	
Stage		
IA	5 (7.14)	
IB	8 (11.43)	
IIA	10 (14.29)	
IIB	16 (22.86)	
IIIA	8 (11.43)	
IIIB	11 (15.71)	
IIIC	11 (15.71)	
IV	1 (1.43)	
TNM stage		
T1N0M0	5 (7.14)	
T1N1M0	3 (4.29)	
T2N0M0	5 (7.14)	
T2N1M0	3 (4.29)	
T2N2M0	2 (2.86)	
T3N0M0	7 (10.00)	
T3N1M0	7 (10.00)	
T3N2M0	1 (1.43)	
T3N3M0	4 (5.71)	
T4N0M0	7 (10.00)	
T4N1M0	7 (10.00)	
T4N2M0	7 (10.00)	
T4N2M1	1 (1.43)	
T4N3M0	11 (15.71)	
Stage group		
I	13 (18.57)	
II	26 (37.14)	
III	30 (42.86)	
IV	1 (1.43)	
T stage		
T1	8 (11.43)	
T2	10 (14.29)	
T3	19 (27.14)	
T4	33 (47.14)	
N stage		
N0	24 (34.29)	
N1	20 (28.57)	
N2	11 (15.71)	
N3	15 (21.43)	
M stage		
M0	69 (98.57)	
M1	1 (1.43)	
Histologic grade		
moderately differentiated	14 (20.00)	
poorly differentiated	55 (78.57)	
well differentiated	1 (1.43)	
Histologic type		
adenocarcinoma	46 (65.71)	
adenocarcinoma with neuroendocrine carcinoma	1 (1.43)	
mucinous adenocarcinoma	8 (11.43)	
papillary adenocarcinoma	2 (2.86)	
signet ring cell carcinoma	12 (17.14)	
tubular adenocarcinoma	1 (1.43)	
OS status		
Alive	51 (72.86)	
Dead	11 (15.71)	
unknown	8 (11.43)	
PFS status		
No_progression	46 (65.71)	
Progression	16 (22.86)	
unknown	8 (11.43)	
STAD-MSC subtype		
LI-STAD	31 (44.29)	
MI-STAD	14 (20.00)	
HI-STAD	25 (35.71)	

STAD-MSC, stomach adenocarcinoma molecular subtype classification; OS, overall survival; PFS, progression-free survival; T, tumor tissue; N, normal tissue.

The selection of AXL, COL1A1, and NNMT for immunohistochemical (IHC) validation was driven by their complementary representation of three key pathophysiological dimensions, namely immunoreceptor regulation, stromal architecture, and metabolic epigenetic modulation, which collectively are associated with the myeloid stromal interaction network underlying the STAD-MSC phenotype. Specifically, AXL, a critical receptor tyrosine kinase preferentially expressed on myeloid cells, particularly tumor associated macrophages (TAMs) (39), transduces GAS6 dependent signaling to orchestrate immunosuppressive M2 polarization, functioning as a molecular switch for microenvironmental immune evasion (40, 41). COL1A1, abundantly secreted by cancer associated fibroblasts (CAFs) (42), undergoes cross linking to augment matrix stiffness, which not only physically impedes T-cell infiltration and might influence a pro tumorigenic macrophage phenotypic shift through mechanotransduction pathways (43, 44). Concurrently, NNMT acts as a methyl sink by depleting the methyl donor S-adenosylmethionine (SAM) (45), thereby driving epigenetic reprogramming that transcriptionally upregulates a panel of inflammatory mediators, including IL-6, GM-CSF, and SAA; this cascade efficiently recruits and polarizes myeloid derived suppressor cells (MDSCs) (46), and even drives the transdifferentiation from macrophage to myofibroblast, directly amplifying collagen deposition and reinforcing the stromal barrier (47).Collectively, these three markers may participate in a network in which NNMT related metabolic remodeling, COL1A1 mediated stromal stiffening, and AXL associated immunosuppression could be interconnected.

**Figure 7:**
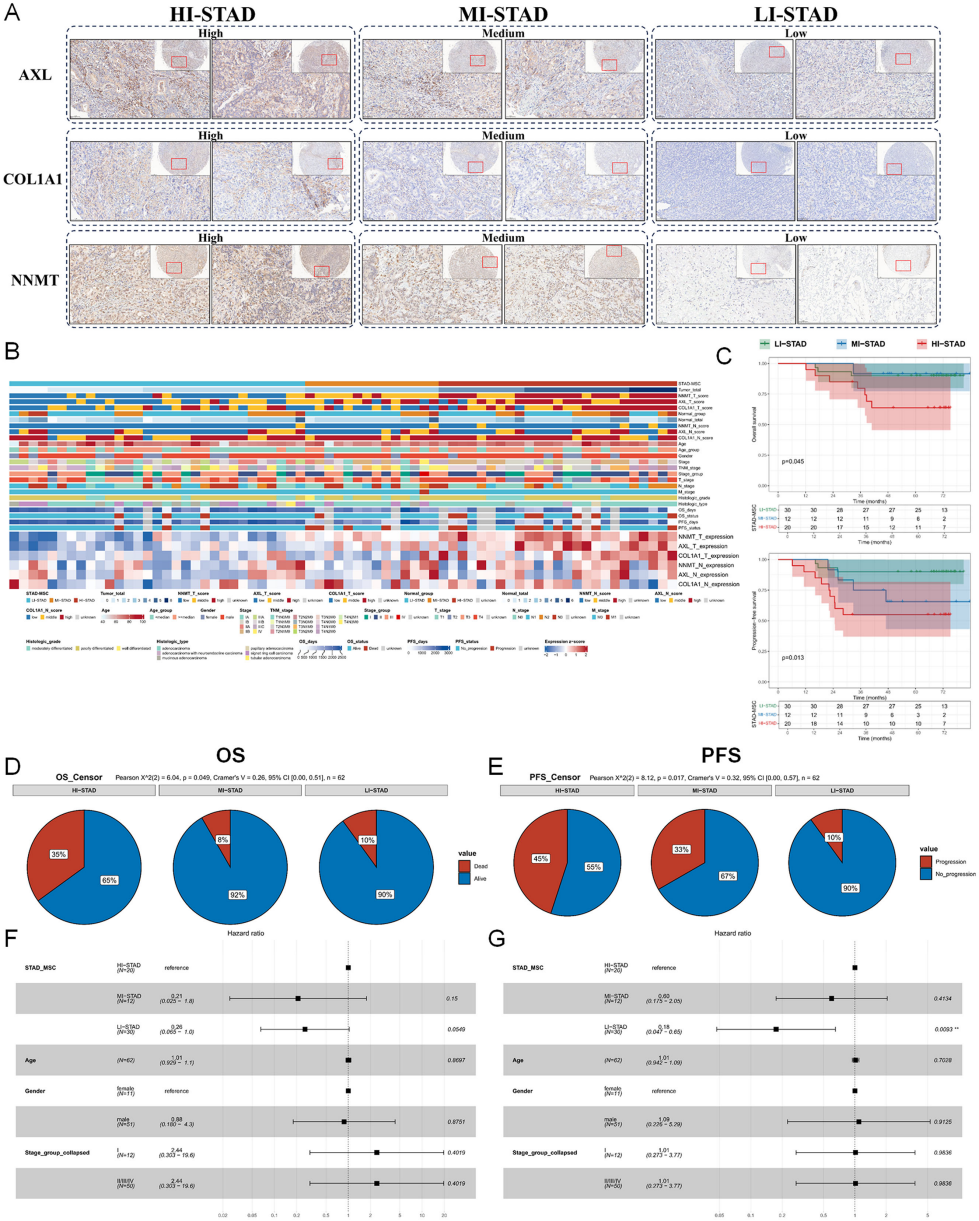
Protein-level clinical validation of the STAD-MSC classification using a three-protein classifier in a 70-patient retrospective cohort. **(A)** Representative IHC images showed the differential staining results of AXL, COL1A1, and NNMT in three STAD-MSC subtypes. Specifically, AXL, COL1A1, and NNMT showed high, medium, and low staining in HI-STAD, MI-STAD, and LI-STAD, respectively. **(B)** The heatmap illustrated the information of the 70 patients in our retrospective cohort. **(C)** The Kaplan-Meier curves showed patients in the HI-STAD cluster had the lowest OS (p = 0.045) and PFS (p = 0.013) in STAD patients. In the OS analysis, Cox proportional hazards regression was performed using HI-STAD as the reference group. LI-STAD showed a reduced risk of death [HR = 0.25, 95% CI = 0.07–0.98], whereas MI-STAD showed a reduced risk [HR = 0.20, 95% CI = 0.03–1.66]. In the PFS analysis, using HI-STAD as the reference group, LI-STAD showed a reduced risk of progression or death [HR = 0.17, 95% CI = 0.05–0.63], whereas MI-STAD showed a reduced risk [HR = 0.58, 95% CI = 0.18–1.90]. **(D)** The pie chart demonstrated that HI-STAD conferred higher probability of death (35%) among patients with available follow-up data, while MI-STAD and LI-STAD were 8% and 10% respectively. **(E)** The pie chart suggested that HI-STAD conferred higher probability of PFS event (45%) among patients with available follow-up data, while MI-STAD and LI-STAD were 33% and 10% respectively. **(F)** Multivariate proportional hazards Cox regression analysis showed that LI-STAD exhibited a borderline protective trend for OS (HR = 0.26, 95% CI = 0.065–1.0, *p* = 0.0549). **(G)** Multivariate proportional hazards Cox regression analysis identified LI-STAD as significantly associated with improved PFS (HR = 0.18, 95% CI = 0.047–0.65, *p* = 0.0093). KM, Kaplan-Meier; STAD, stomach adenocarcinoma; STAD-MSC, stomach adenocarcinoma myeloid-state classification; LI-STAD, low immune infiltration STAD; MI-STAD, moderate immune infiltration STAD; HI-STAD, high immune infiltration STAD; OS, overall survival; PFS, progression-free survival; CI, confidence interval; AUC, area under the curve.

Representative IHC staining results for NNMT, AXL, and COL1A1 are shown in **Figure 7A**. Based on the protein-level scoring and subtyping system, we classified the patients as LI-STAD (44.29%), MI-STAD (20.00%), and HI-STAD (35.71%), respectively. The KM survival plots demonstrated that patients in the HI-STAD group exhibited the lowest OS and PFS (**Figure 7C**, OS, *p* = 0.045; PFS, *p* = 0.013). Moreover, **Figure 7B** shows the distribution of STAD-MSC subtypes together with clinicopathological features, OS/PFS status, and protein expression scores. With regard to clinical outcomes, HI-STAD displayed the highest proportion of death (35%, **Figure 7D**) and PFS progression (45%, **Figure 7E**); the death rates in MI-STAD and LI-STAD were 8% and 10%, while the corresponding PFS progression rates were 33% and 10%, respectively. Survival-related analyses were based on the 62 patients with available follow-up data. More importantly, **Figure 7F****-G** showed that LI-STAD exhibited a borderline protective trend for OS (HR = 0.26, 95% CI = 0.065–1.0, *p* = 0.0549) and a significant protective association for PFS (HR = 0.18, 95% CI = 0.047–0.65, *p* = 0.0093). Subsequently, we assessed the proportional hazards assumption using residual plots and Schoenfeld tests. Given the limited number of events, additional discrimination or calibration analyses were considered exploratory. Taken together, the three-protein classifier provides preliminary evidence supporting the feasibility of translating the transcriptome-defined STAD-MSC classification to the protein level. However, given the modest cohort size and the limited number of observed outcome events (11 deaths and 16 PFS events among 62 patients with follow-up), these findings should be regarded as exploratory and require further validation in larger independent cohorts.

## 4 Discussion

Stomach adenocarcinoma frequently exhibits high levels of histological, transcriptomic, and genomic variations, leading to distinct clinical behaviors and therapeutic responses, which poses significant challenges in interpreting its biological characteristics and patients’ differential prognosis (48). Recent studies have shown that the STAD tumor microenvironment is heavily infiltrated by myeloid cells, particularly TAMs, which are pivotal in driving immunosuppression and contribute to this heterogeneity. Specifically, they secrete potent immunosuppressive cytokines, such as interleukin-10 (IL-10) and transforming growth factor-β (TGF-β), which directly inhibit the effector functions of cytotoxic CD8+ T cells (49, 50). Furthermore, TAM-derived chemokines, including CCL2 and CCL22, facilitate the recruitment and accumulation of additional regulatory immune cell populations, thereby amplifying the immunosuppressive network (51, 52). To address the unresolved relationship among myeloid cell states, TME features, and STAD prognosis, we conducted a comprehensive bioinformatic and clinical study.

### 4.1 From myeloid states to clinical subtypes and therapeutic implications of the STAD-MSC framework

By integrating scRNA-seq with Monocle 2 pseudotime analysis, we delineated a set of genes critically associated with myeloid cell states, termed the MSRGs. The Monocle2 trajectory was used as a feature-discovery strategy to identify myeloid state-associated genes; pseudotime ordering should not be interpreted as definitive evidence of lineage directionality or causal differentiation without experimental validation. We then derived the MSRPGs by intersecting these MSRGs with genes significantly associated with OS in STAD, yielding a final set of 32 genes. Subsequently, consensus clustering of these MSRPGs led to the establishment of the STAD-MSC. Within this framework, the HI-STAD subtype was identified as having the poorest clinical prognosis and most extensive immune cell infiltration. Following this, in silico pharmacogenomic screening nominated several hypothesis generating candidate compounds for the HI-STAD subtype, including ruxolitinib, 5-fluorouracil, dabrafenib, and motesanib. Moreover, leveraging the MSRPGs, we developed a risk-score-based prognostic model. We further evaluated this model in a public 355-patient validation cohort, which supported its reproducibility at the risk-score level. Importantly, the STAD-MSC classification was corroborated at the protein level in an independent 70-patient cohort using an IHC-based three-protein classifier (NNMT/AXL/COL1A1), supporting the feasibility of protein-level translation, pending larger independent validation.

Based on this classification, the three STAD-MSC subtypes (HI-STAD, MI-STAD, and LI-STAD) displayed markedly distinct immune infiltration patterns and functional states. Specifically, the HI-STAD subtype, while demonstrating the highest overall level of immune cell infiltration, exhibited a microenvironment dominated by pro-tumorigenic myeloid lineages and phenotypically exhausted lymphoid populations. Stratification by PCA score, where a low score correlated with HI-STAD assignment and poorer prognosis, revealed that the low-PCA group possessed significantly elevated scores for T-cell dysfunction and exclusion. This was concomitant with heightened expression of canonical immune checkpoint molecules, including CTLA-4, PD-L1 and PD-1.

Further quantitative analyses elucidated the suppressive nature of this infiltration. The high-risk (low-PCA) group presented significantly elevated scores for CAF abundance, T-cell dysfunction, T-cell exclusion, and the composite TIDE metric. The increased CAF score denotes a stroma-rich, fibrotic microenvironment conducive to creating physical barriers and fostering immunosuppressive signaling (53). The concurrently high dysfunction and exclusion scores provide quantitative evidence that tumor-infiltrating T cells are not only functionally impaired but also physically sequestered from malignant cells, consistent with an immune excluded phenotype (54). Although HI-STAD showed the highest overall immune infiltration, this infiltration was accompanied by increased CAF abundance, T-cell dysfunction, T-cell exclusion, and elevated immune checkpoint expression. Cell-cell communication analysis further suggested extensive myeloid-centered ligand-receptor interactions, including FN1-PLAUR, MMP12-PLAUR, CXCL8/CXCL2/CXCL3-CXCR2, IL1B-IL1R2, and APOE-LDLR. These interactions are consistent with a myeloid-rich suppressive niche involving extracellular matrix remodeling, inflammatory chemokine signaling, and receptor-mediated communication. However, because these analyses are based on association and ligand-receptor inference, they do not establish whether myeloid accumulation drives T-cell dysfunction or occurs secondarily after immune dysfunction. We therefore interpret HI-STAD as an immune-infiltrated but functionally suppressed phenotype and emphasize that causal directionality requires further experimental validation.

Exploratory pharmacological profiling suggested nominal trends toward differential sensitivity for compounds such as ruxolitinib and dabrafenib in the HI-STAD subtype. However, given the exploratory nature of these in silico findings, they should be interpreted as hypothesis-generating rather than conclusive. Functional validation in appropriate preclinical models is warranted to assess the therapeutic relevance of these candidate vulnerabilities.

### 4.2 Biological functions of MSRPGs in defining a state-associated risk axis

Based on their expression dynamics along the pseudotime trajectory, the MSRPGs were categorized into distinct states, reflecting their association with early, intermediate, or terminal myeloid cell states. The differential expression of these genes across the three STAD-MSC subtypes defined the unique molecular profiles of each.

All the MSRPGs of state 1 were risk factors and had the highest expression in HI-STAD. The majority were reported to play critical roles in driving immunosuppression, remodeling cellular metabolism, and facilitating angiogenesis (55–66). Among them, the elevated expression of canonical immunosuppressive markers (e.g., PD-L1/CD274) significantly affirmed the strongly inhibitory immune phenotype of HI-STAD (67). AXL is a member of the TAM receptor tyrosine kinase (RTKs) family (68). By binding to its primary ligand, the growth arrest-specific protein 6 (GAS6), AXL participates in various signal transduction cascades and plays a critical role in various biological processes including cell proliferation, survival, migration, efferocytosis, angiogenesis, platelet aggregation and fibrosis (69, 70). For example, in lung cancers, AXL interacts with epidermal growth factor receptor (EGFR) and human epidermal growth factor receptor 3 (ERBB3) to maintain the activation status of downstream signaling pathways, which confers intrinsic resistance to osimertinib in non-small cell lung cancer (NSCLC) cells (71). In colorectal cancers, AXL induces the expression of Twist family BHLH transcription factor 1 (TWIST1) and mediates resistance to polo-like kinase 1 (PLK1) inhibitor (72). Neuropilin-1 (NRP1) is recognized as a multifunctional co-receptor, forming complexes with other membrane receptors (73). It acts as a co-receptor for various growth factors, including vascular endothelial growth factor (VEGF) and transforming growth factor beta (TGF-β) contributing to intricate signaling pathways that influence biological processes such as cell migration, angiogenesis, and immune responses (74).

In state 3 (intermediate state), immune and stroma-related genes such as CXCR4 and THBS2 were highly expressed in HI-STAD. The CXCR4, controlled by its full agonist ligand CXCL12, plays pleiotropic functions in diverse physiological processes, such as hematopoiesis, immune cell development, and trafficking (75, 76). Many solid tumors upregulate CXCL12 and exploit the CXCL12-CXCR4 axis to orchestrate an immunosuppressive microenvironment by recruiting PMN-MDSCs and repelling T cells (77, 78). THBS2 promotes STAD progression through the Notch signaling pathway by regulating Notch3, HEY1, and HES1 proteins, and sustains cancer stem cell-like characteristics by via Notch3, including the expression of CD44, Nanog, OCT4, and SOX2 (79). In summary, the concerted biological functions and complex interactions of the MSRPGs across different states support their role as a state-associated risk axis. This axis provides a molecular framework to explain the functional differences among STAD-MSC subtypes, including TAM polarization, ECM/EMT, angiogenesis, and fibroblast-myeloid interaction.

Because the subtype-specific networks were constructed primarily from correlation analyses, they should be interpreted as association-based regulatory models. Co-expression between transcription factors, MSRPGs, immune signatures, and downstream pathways does not establish direct regulatory causality, and functional experiments are required to confirm these relationships.

The five-gene signature was objectively derived from the 32 MSRPGs via LASSO regression. Functionally, it captures a coordinated microenvironmental risk axis: SPARC reflects matrix remodeling, while PLOD2 mediates collagen maturation (80, 81). TFPI represents angiogenesis-related signaling, and CXCR4 mediates immune-cell trafficking and exclusion (82, 83). BEX3 may contribute to stress-adaptive survival (84). Thus, the combined model integrates stromal activation, ECM remodeling, inflammatory communication, and immune exclusion, which may explain its superior prognostic performance over isolated markers.

### 4.3 Comparison with existing gastric cancer classification systems

Several classification systems have been established for gastric cancer, notably the TCGA molecular subtypes and the ACRG molecular subtypes. The TCGA classification, derived from comprehensive multi-omics profiling, categorizes tumors into EBV-positive, MSI, CIN, and GS subtypes, providing valuable insights into genomic drivers and potentially targetable alterations (6). The ACRG classification, based on gene expression microarrays in Asian cohorts, stratifies tumors into MSI, MSS/EMT, MSS/TP53+, and MSS/TP53− subtypes, with demonstrated strength in prognostic stratification, particularly for identifying the high-risk MSS/EMT phenotype (85). Both systems primarily emphasize genomic and epithelial transcriptomic features, whereas the contributions of the tumor microenvironment particularly myeloid immune cells, are relatively less explored in these frameworks.

STAD-MSC was designed with a focus on myeloid state-associated tumor microenvironment programs. By anchoring classification in the functional states of myeloid cells along their differentiation trajectories, this framework links myeloid-derived genes to immune infiltration, T-cell dysfunction, and clinical prognosis. The system can be approximated by a three-protein immunohistochemistry-based classifier (NNMT, AXL, and COL1A1), which may facilitate microenvironmental assessment in routine pathology settings without requiring specialized sequencing platforms. Furthermore, STAD-MSC identifies the immune-infiltrated yet immunosuppressive HI-STAD phenotype, a feature that may not be readily captured by genomic-centric taxonomies and could offer additional information for patient stratification. At the same time, we acknowledge that further prospective validation in larger, independent cohorts is needed to define the clinical utility of this framework. Overall, STAD-MSC is best viewed as complementary to, rather than a replacement for, established classifications, providing a microenvironment-oriented perspective that addresses a dimension relatively underrepresented in current gastric cancer taxonomy.

Several limitations of this study warrant consideration. First, although our study incorporates both public-cohort validation and a protein-level retrospective cohort, all analyses remain retrospective; prospective studies and real-world cohorts treated with immune checkpoint inhibitors are still needed for further confirmation. Although the 5-gene risk model was developed and internally evaluated in the TCGA cohort and externally validated in the GSE62254 and GSE84437 cohorts and the NNMT/AXL/COL1A1 classifier was assessed in an independent protein-level clinical cohort, the transcriptomic STAD-MSC subtype classification itself was derived from TCGA-STAD. Because the subtype discovery involved sequential filtering from pseudotime-associated genes to survival-associated genes and consensus clustering, potential overfitting and circularity cannot be fully excluded. Independent transcriptomic cohorts with comparable data processing will be required to further validate the reproducibility and generalizability of STAD-MSC subtype assignment. The retrospective protein-level validation cohort was limited in size, with 62 patients available for follow-up and 11 OS and 16 PFS events. Therefore, the multivariable Cox regression should be interpreted as exploratory, and their prognostic modeling performance requires validation in larger independent cohorts. In addition, inferences drawn from integrated single-cell RNA sequencing data may be influenced by limited sample sizes and inherent technical batch effects. Furthermore, the correlative nature of our computational approach establishes associations but does not prove causality between the identified myeloid states and tumor progression. Similarly, although ATAC-seq and CNV analyses provide supportive multi-omics evidence, they do not establish direct regulatory causality; the subtype-specific regulatory networks should therefore be interpreted as association-based models that require further functional validation. Additionally, the protein-level cohort included 70 patients, of whom 62 had follow-up data; only 11 deaths and 16 PFS events were observed, limiting statistical power and the reliability of multivariable model-performance estimates. Finally, it is critical to emphasize that the potential therapeutic implications discussed herein, including the predicted sensitivity to compounds such as ruxolitinib, dabrafenib, 5-fluorouracil, and motesanib, are derived from computational inference rather than experimental evidence. These in silico predictions should be viewed as hypothesis-generating and must be rigorously validated in appropriate gastric cancer models before any therapeutic relevance can be claimed. Future studies should prioritize validating the STAD-MSC subtyping framework in independent, prospectively collected cohorts. Employing spatial transcriptomics and multiplex immunohistochemistry would be invaluable to visually confirm the predicted cellular geography and interactions within the tumor microenvironment. Ultimately, functional experiments are required to definitively establish the mechanistic roles of key regulators in mediating the immunosuppressive phenotype associated with the high-risk subtype.

## 5 Conclusion

In summary, we established and preliminarily evaluated STAD-MSC, a novel molecular taxonomy for stomach adenocarcinoma that stratifies patients into three prognostic groups (HI-STAD, MI-STAD, LI-STAD) based on myeloid cell transcriptional states. The 5-gene risk model was internally evaluated in the TCGA cohort and externally validated in the GSE62254 and GSE84437 cohorts, and the NNMT/AXL/COL1A1 classifier provided protein-level clinical stratification. The high-risk HI-STAD subtype is characterized by high expression of SPARC, THBS2, and CXCR4 in myeloid cells and is associated with poor outcomes. This work provides a framework for immune informed patient stratification and nominates exploratory therapeutic hypotheses that require functional validation in preclinical models.

## Data Availability

The publicly available datasets analyzed in this study can be found in the Gene Expression Omnibus (GSE183904, GSE62254, and GSE84437) and The Cancer Genome Atlas stomach adenocarcinoma project (TCGA-STAD). Additional public resources used in the analyses included the Cancer Cell Line Encyclopedia, Genomics of Drug Sensitivity in Cancer, Cistrome Data Browser, and Molecular Signatures Database. De-identified data from the retrospective Ruijin Hospital cohort are available from the corresponding author upon reasonable request, subject to institutional ethical and privacy requirements.

## Glossary

STAD: stomach adenocarcinoma
scRNA-seq: single-cell RNA sequencing
MSRPGs: myeloid state-related prognostic genes
STAD-MSC: stomach adenocarcinoma myeloid-state classification
UMAP: uniform manifold approximation and projection
MSRGs: myeloid state-related genes
OS: overall survival
DEGs: differentially expressed genes
GSVA: gene set variation analysis
BEAM: branched expression analysis modeling
TCGA: The Cancer Genome Atlas
KM: Kaplan-Meier
LI-STAD: low immune infiltration STAD
MI-STAD: moderate immune infiltration STAD
HI-STAD: high immune infiltration STAD
MDSCs: myeloid-derived suppressor cells
PCA: principal component analysis
CAF: cancer-associated fibroblast
TIDE: tumor immune dysfunction and exclusion
TF: transcription factor
ssGSEA: single-sample gene set enrichment analysis
LASSO: least absolute shrinkage and selection operator
KEGG: Kyoto Encyclopedia of Genes and Genomes
H-PCA: high PCA score group
L-PCA: low PCA score group
TMB: tumor mutation burden
MSI: microsatellite instability
L-TMB: low TMB group
H-TMB: high TMB group
CNV: copy number variation
DETFs: differentially expressed transcription factors
ATAC-seq: assay for transposase-accessible chromatin sequencing
ORA: over-representation analysis
ROC: receiver operating characteristic
ICB: immune checkpoint blockade
EMT: epithelial-mesenchymal transition
CCLE: cancer cell line encyclopedia
GEO: Gene Expression Omnibus
CIN: chromosomal instability
pDCs: plasmacytoid dendritic cells
cDCs: conventional dendritic cells
TAM: tumor-associated macrophages
TAMos: tumor-associated monocytes
ECM: extracellular matrix
DCs: dendritic cells
GDSC: Genomics of Drug Sensitivity in Cancer
MSigDB: the Molecular Signatures Database
PCs: principal components
IPS: immunophenoscore
AUC: area under the curve
GO: Gene Ontology
HR: Hazard Ratio
CI: confidence interval
