# Supplementary material for: Integrated single-cell and bulk RNA sequencing analyses identify a myeloid state-related gene signature for molecular subtyping in stomach adenocarcinoma
Source: Front Immunol. 2026 Jul 16;17:1887429. doi: 10.3389/fimmu.2026.1887429 (PMC13422448; doi:10.3389/fimmu.2026.1887429)
Supplement: Supplementary file 3 [file DataSheet1.docx]

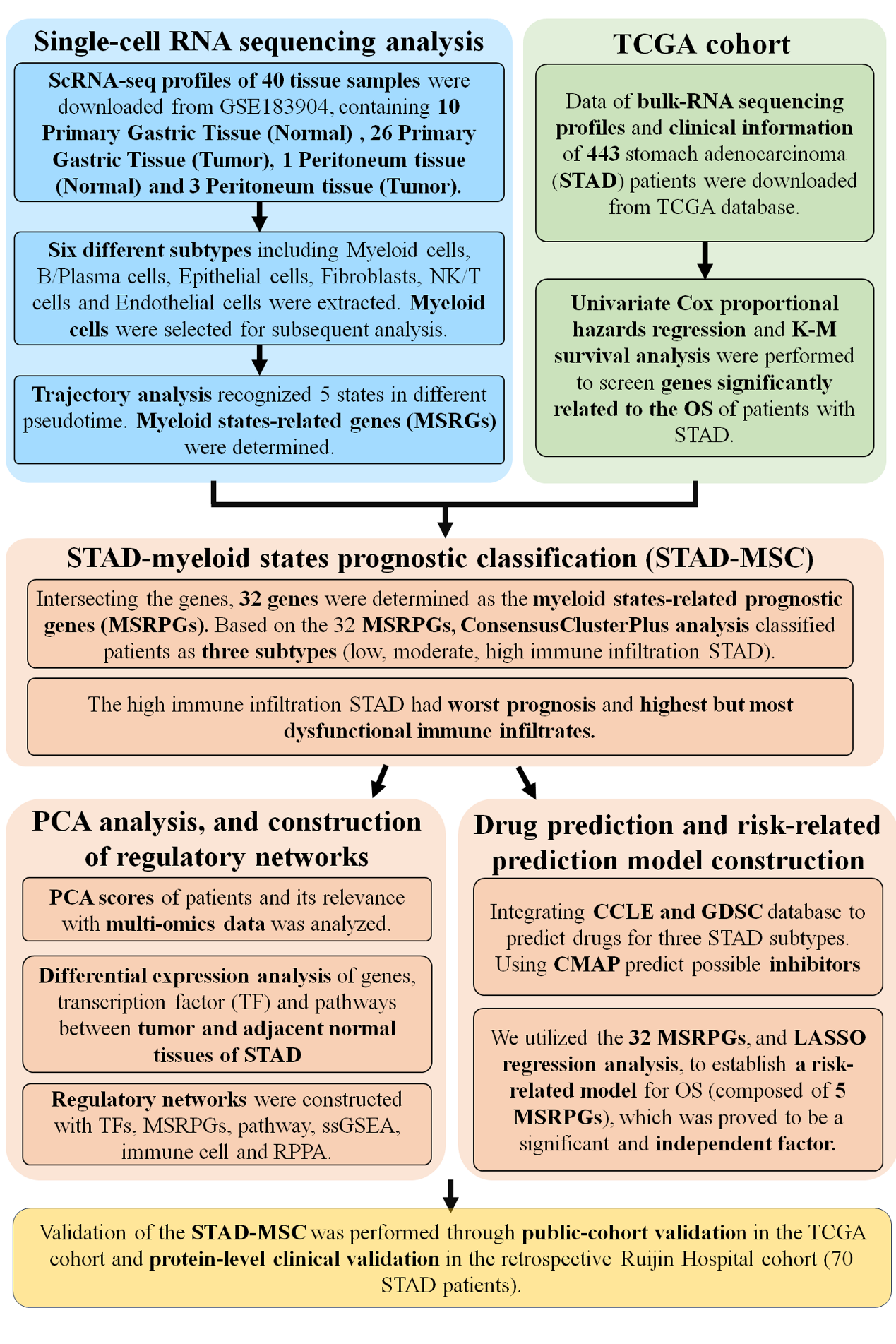


**Fig. S1** The detailed flowchart of our study.

The flowchart is primarily composed of 4 parts. In the blue part, we performed scRNA-seq analysis, specifically extracting the myeloid cells. Through monocle 2 trajectory analysis, we identified five myeloid states and defined MSRGs. In the green part, using TCGA cohort, we obtained the genes significant in KM survival analysis (KM_sig, p < 0.05) and univariate Cox proportional hazards regression analysis (UniCscrnaox_sig, p < 0.01). In the yellow part, we first intersected the previous three gene sets, and thus identified 32 MSRPGs. Utilizing the expression levels of MSRPGs, we successfully constructed the STAD-MSC with three distinct STAD subtypes. Secondly, we utilized PCA analysis, to validate the prognostic significance of our model. And we explored the relevance of STAD-MSC and multi-omics data. Afterwards, differential expression analysis of genes, TF and cancer hallmarks between tumor and adjacent normal tissues was conducted, based on which the regulatory networks were constructed, together the immune components and proteomics. Thirdly, we predicted the potential inhibitors for three STAD subtypes using CCLE and GDSC databases and we established a risk-related model, proving it as a significant and independent prognostic factor. Finally, validation in the TCGA public cohort confirmed the robustness of the risk model, and protein-level clinical validation in the retrospective Ruijin cohort (70 patients with stomach adenocarcinoma) substantiated the STAD-MSC classification.

STAD-MSC, stomach adenocarcinoma myeloid-state classification; ScRNA-seq, single-cell RNA sequencing; TCGA, The Cancer Genome Atlas; K-M, Kaplan-Meier; MSRPGs, myeloid state-related prognostic genes; STAD, stomach adenocarcinoma; PCA, principal component analysis; TF, transcription factor; CCLE, cancer cell line encyclopedia; GDSC, Genomics of Drug Sensitivity in Cancer;


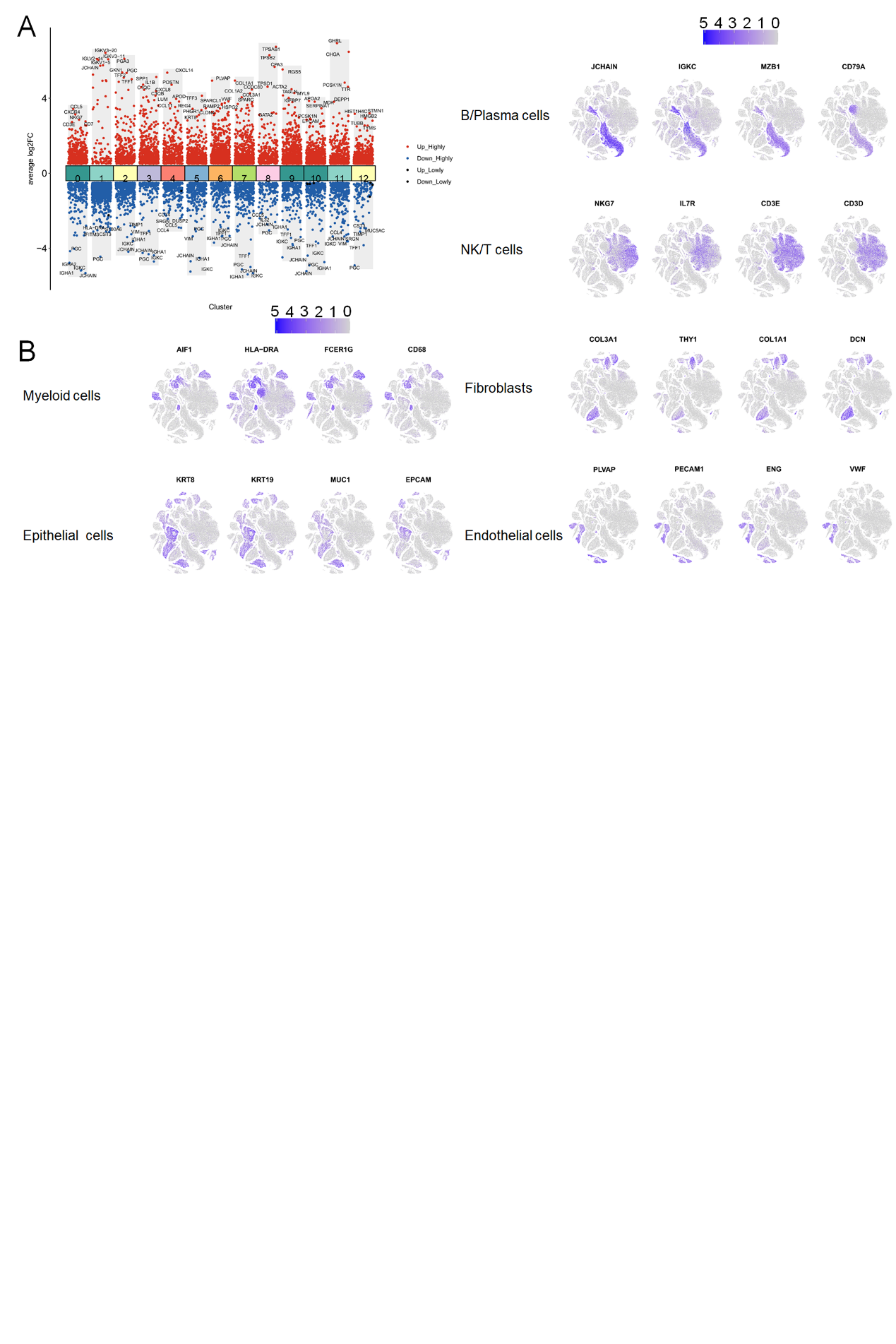


**Fig. S2** Cell markers of each cell type and gene differential expression pattern of the 6 cell clusters

**(A)** We first showed all DEGs in a scatter diagram (p < 0.05, |average log2FC| > 0.5), categorized into four different types, including “Down_Highly” (down-regulated DEGs with p < 0.01 and average log2FC < -0.5), “Down_Lowly” (down-regulated with 0.01 ≤ p < 0.05 and average log2FC < -0.5), “Up_Highly” (up-regulated DEGs with p < 0.01 and average log2FC > 0.5) and “Up_Lowly” (up-regulated DEGs with 0.01 ≤ p < 0.05 and average log2FC > 0.05). **(B)** The marker genes of the cell clusters were specifically demonstrated.

DEGs, differentially expressed genes;


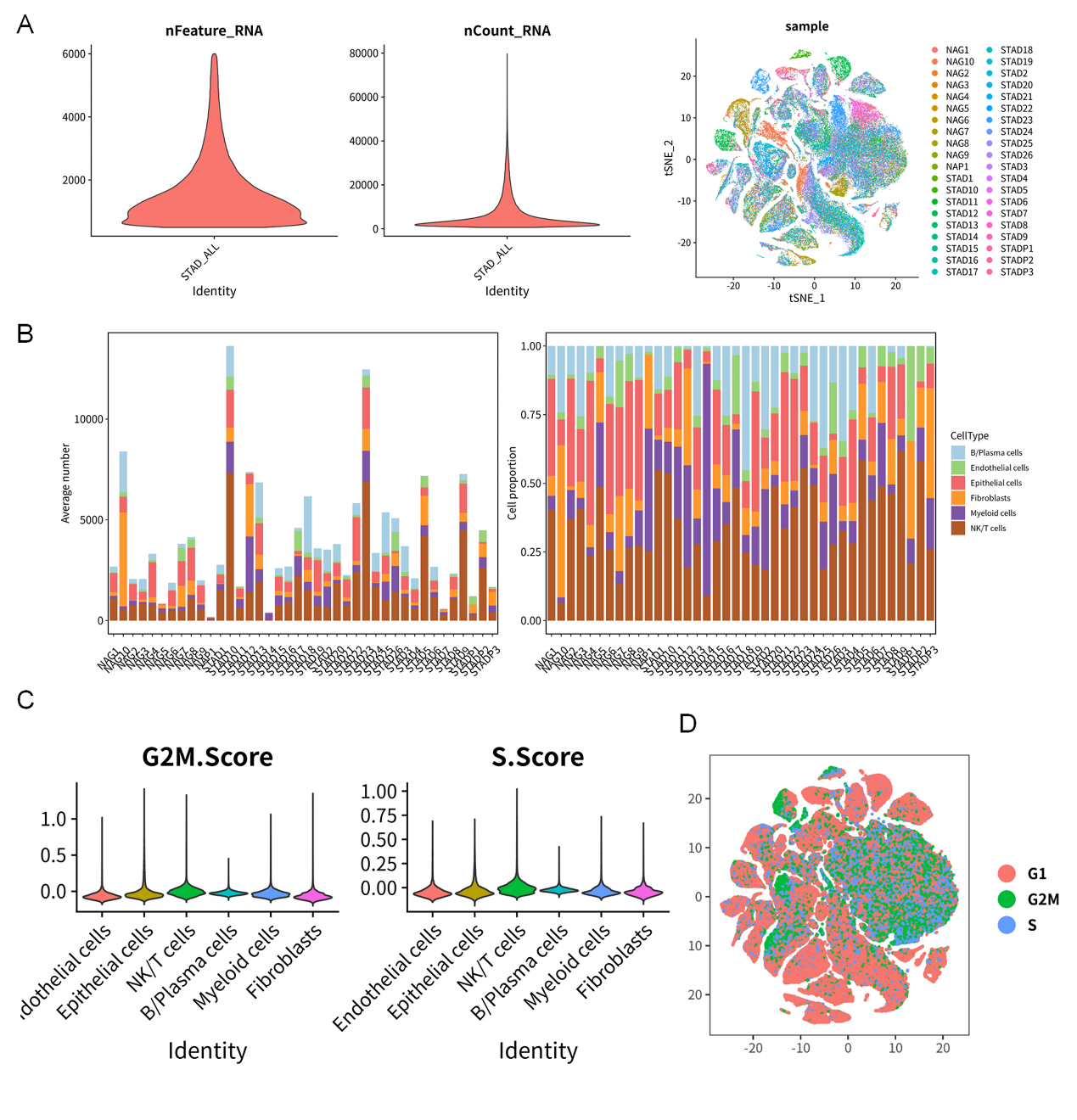


**Fig. S3** Graphical summary of cellular composition, proportions, and cell cycle analysis

**(A)**

**(B)** The bar plots visualized the cell numbers and cell proportions of the 40 sample sources. **(B)** The violin plot displayed the cell cycle scores of G2M and S in each cell type. **(D)** The UMAP visualization displays the spatial distribution of cells across the G1, S, and G2M phases of the cell cycle.

ScRNA-seq, single-cell RNA sequencing; UMAP, uniform manifold approximation and projection;

**Table S1** Table S1. Baseline information of TCGA-STAD patients with available clinical information (n = 414)..

| Sample ID | 0S time (days) | | | 0S status | | age | | gender | | race | | grade | | M | | N | T | | Pathological stage | |
| --- | --- | --- | --- | --- | --- | --- | --- | --- | --- | --- | --- | --- | --- | --- | --- | --- | --- | --- | --- | --- |
| TCGA-CG-5720-01A | | 30 | Dead | | >65 | | male | | not reported | | G3 | | M0 | | N0 | | | T2 | | stage I |
| TCGA-VQ-A8PX-01A | | 1964 | Alive | | <=65 | | male | | not reported | | G2 | | M0 | | N0 | | | T1 | | stage I |
| FTCGA-MX-A663-01A | | 300 | Dead | | >65 | | male | | white | | G3 | | M0 | | N0 | | | T3 | | stage II |
| TCGA-IN-A7NT-01A | | 323 | Alive | | >65 | | female | | white | | G3 | | M0 | | N1 | | | T3 | | stage II |
| TCGA-VQ-A94P-01A | | 81 | Dead | | <=65 | | male | | white | | GX | | MX | | NX | | | T4 | | not reported |
| TCGA-VQ-A91N-01A | | 570 | Dead | | <=65 | | female | | not reported | | G2 | | M0 | | N3 | | | T4 | | stage IV |
| TCGA-HU-8610-01A | | 23 | Alive | | >65 | | male | | asian | | G2 | | M0 | | N0 | | | T1 | | stage I |
| TCGA-BR-7196-01A | | 666 | Alive | | <=65 | | male | | white | | G3 | | M1 | | N3 | | | T3 | | stage IV |
| TCGA-IN-8462-01A | | 572 | Alive | | >65 | | male | | white | | G2 | | M0 | | N1 | | | T2 | | stage II |
| TCGA-D7-6525-01A | | 406 | Dead | | <=65 | | male | | white | | G3 | | M0 | | N2 | | | T2 | | stage III |
| TCGA-CG-5732-01A | | 2100 | Dead | | >65 | | male | | not reported | | G2 | | M0 | | N3 | | | T2 | | stage IV |
| TCGA-BR-4280-01A | | 201 | Dead | | >65 | | female | | white | | G2 | | M0 | | N1 | | | T2 | | stage III |
| TCGA-VQ-A91Y-01A | | 296 | Dead | | >65 | | male | | white | | G3 | | M0 | | N3 | | | T4 | | stage III |
| TCGA-BR-A44U-01A | | 422 | Dead | | >65 | | male | | white | | G3 | | M0 | | N3 | | | T3 | | stage III |
| TCGA-BR-8059-01A | | 439 | Dead | | >65 | | male | | white | | G3 | | M0 | | N1 | | | T4 | | stage III |
| TCGA-KB-A93G-01A | | 613 | Alive | | >65 | | male | | white | | G1 | | M0 | | N0 | | | T2 | | stage I |
| TCGA-HU-A4GN-01A | | 912 | Alive | | <=65 | | male | | asian | | G2 | | M0 | | N1 | | | T2 | | stage II |
| TCGA-VQ-A8DV-01A | | 403 | Dead | | <=65 | | male | | white | | G2 | | M0 | | N0 | | | T2 | | stage I |
| TCGA-BR-A4IZ-01A | | 273 | Dead | | <=65 | | female | | asian | | G3 | | M0 | | N2 | | | T4 | | stage III |
| TCGA-F1-A72C-01A | | 346 | Alive | | >65 | | male | | asian | | G2 | | M0 | | N0 | | | T3 | | stage II |
| TCGA-BR-A4IV-01A | | 869 | Dead | | <=65 | | male | | asian | | G3 | | M0 | | N2 | | | T4 | | stage III |
| TCGA-D7-5579-01A | | 636 | Alive | | >65 | | male | | white | | G3 | | M0 | | N2 | | | T3 | | stage III |
| TCGA-MX-A5UG-01A | | 113 | Dead | | >65 | | male | | asian | | G3 | | M0 | | N1 | | | T3 | | stage III |
| TCGA-BR-8683-01A | | 300 | Dead | | >65 | | male | | asian | | G3 | | M0 | | N2 | | | T4 | | stage III |
| TCGA-CG-4437-01A | | 245 | Alive | | >65 | | male | | not reported | | G2 | | M0 | | N1 | | | T2 | | stage II |
| TCGA-HU-A4HD-01A | | 1016 | Alive | | >65 | | male | | asian | | G2 | | M0 | | N2 | | | T3 | | stage III |
| TCGA-D7-A74B-01A | | 217 | Dead | | <=65 | | female | | white | | G3 | | M0 | | N3 | | | T3 | | stage III |
| TCGA-BR-4187-01A | | 141 | Dead | | <=65 | | male | | white | | G3 | | M0 | | NX | | | TX | | not reported |
| TCGA-R5-A7O7-01A | | 1389 | Alive | | <=65 | | male | | white | | G2 | | M1 | | N1 | | | T3 | | stage IV |
| TCGA-B7-5818-01A | | 356 | Alive | | <=65 | | male | | white | | G3 | | M0 | | N0 | | | T2 | | stage I |
| TCGA-FP-8631-01A | | 17 | Alive | | >65 | | male | | white | | G2 | | MX | | N2 | | | T3 | | stage III |
| TCGA-FP-8209-01A | | 1811 | Dead | | <=65 | | male | | white | | G3 | | M0 | | N0 | | | T2 | | stage I |
| TCGA-CG-5724-01A | | 366 | Dead | | <=65 | | male | | not reported | | G3 | | M1 | | N3 | | | T3 | | stage IV |
| TCGA-D7-A4YU-01A | | 500 | Alive | | >65 | | male | | white | | G3 | | M0 | | N3 | | | T3 | | stage III |
| TCGA-CG-4466-01A | | 577 | Alive | | >65 | | female | | not reported | | G2 | | M0 | | N0 | | | T2 | | stage I |
| TCGA-CD-A489-01A | | 344 | Dead | | <=65 | | male | | asian | | G3 | | M0 | | N0 | | | T3 | | stage II |
| TCGA-D7-6520-01A | | 573 | Alive | | <=65 | | male | | white | | G3 | | M0 | | N2 | | | T2 | | stage III |
| TCGA-CG-4477-01A | | 942 | Alive | | <=65 | | female | | not reported | | G3 | | M0 | | N0 | | | T2 | | stage I |
| TCGA-D7-A6EZ-01A | | 618 | Dead | | >65 | | male | | white | | G3 | | M0 | | N2 | | | T3 | | stage III |
| TCGA-CG-4475-01A | | 699 | Alive | | >65 | | male | | not reported | | G3 | | M0 | | N1 | | | T3 | | stage II |
| TCGA-BR-7717-01A | | 552 | Dead | | <=65 | | male | | white | | G2 | | M0 | | N1 | | | T4 | | stage IV |
| TCGA-HU-A4H5-01A | | 724 | Alive | | >65 | | male | | asian | | GX | | M0 | | N0 | | | T2 | | stage I |
| TCGA-HU-A4H0-01A | | 64 | Alive | | >65 | | male | | asian | | G3 | | M0 | | N3 | | | T4 | | stage III |
| TCGA-BR-8677-01A | | 813 | Alive | | >65 | | female | | white | | G3 | | M0 | | N3 | | | T3 | | stage III |
| TCGA-D7-A6F2-01A | | 476 | Alive | | <=65 | | male | | white | | G3 | | M0 | | N0 | | | T2 | | stage I |
| TCGA-VQ-AA68-01A | | 1328 | Alive | | <=65 | | female | | white | | G2 | | M0 | | N3 | | | T4 | | stage III |
| TCGA-RD-A8MV-01A | | 3720 | Alive | | <=65 | | male | | white | | G3 | | M0 | | N2 | | | T3 | | stage III |
| TCGA-BR-4267-01A | | 188 | Dead | | <=65 | | male | | white | | G2 | | M0 | | N0 | | | T2 | | stage I |
| TCGA-R5-A7ZF-01A | | 259 | Dead | | <=65 | | female | | black | | G2 | | M1 | | N1 | | | T4 | | stage IV |
| TCGA-CD-5803-01A | | 341 | Dead | | >65 | | female | | asian | | G3 | | M0 | | N0 | | | T3 | | stage II |
| TCGA-BR-A4J1-01A | | 22 | Dead | | <=65 | | male | | asian | | G3 | | M0 | | N1 | | | T4 | | stage III |
| TCGA-VQ-AA6B-01A | | 1002 | Alive | | <=65 | | male | | white | | G2 | | M0 | | N2 | | | T3 | | stage III |
| TCGA-RD-A8N6-01A | | 272 | Dead | | >65 | | female | | white | | G3 | | M0 | | N2 | | | T2 | | stage III |
| TCGA-CG-5734-01A | | 243 | Dead | | >65 | | male | | not reported | | G3 | | M0 | | N1 | | | T3 | | stage III |
| TCGA-D7-6527-01A | | 312 | Dead | | <=65 | | male | | white | | G2 | | M0 | | N1 | | | T2 | | stage II |
| TCGA-BR-6852-01A | | 1367 | Alive | | <=65 | | female | | white | | G3 | | M0 | | N0 | | | T3 | | stage II |
| TCGA-IN-A6RN-01A | | 594 | Alive | | >65 | | female | | white | | G2 | | M0 | | N2 | | | T1 | | stage II |
| TCGA-BR-A453-01A | | 185 | Dead | | <=65 | | male | | asian | | G3 | | M1 | | N3 | | | T4 | | stage IV |
| TCGA-CG-4301-01A | | 92 | Alive | | >65 | | female | | not reported | | G3 | | M1 | | N1 | | | T4 | | stage IV |
| TCGA-HU-8238-01A | | 46 | Alive | | <=65 | | male | | asian | | G2 | | M0 | | NX | | | T3 | | not reported |
| TCGA-IN-A6RL-01A | | 406 | Dead | | >65 | | male | | white | | G2 | | M0 | | N1 | | | T2 | | not reported |
| TCGA-FP-7916-01A | | 428 | Dead | | >65 | | male | | asian | | G3 | | MX | | N3 | | | T4 | | stage III |
| TCGA-D7-A74A-01A | | 607 | Alive | | <=65 | | female | | white | | G2 | | M0 | | N2 | | | T3 | | stage III |
| TCGA-HU-A4H8-01A | | 428 | Dead | | >65 | | male | | asian | | G2 | | M0 | | N1 | | | T1 | | stage I |
| TCGA-BR-7851-01A | | 574 | Dead | | >65 | | male | | white | | G3 | | M0 | | N0 | | | T4 | | stage II |
| TCGA-BR-8078-01A | | 900 | Alive | | >65 | | female | | white | | G3 | | M0 | | N0 | | | T4 | | stage II |
| TCGA-BR-A44T-01A | | 1038 | Alive | | <=65 | | female | | white | | G3 | | M0 | | N0 | | | T3 | | stage II |
| TCGA-IN-A6RJ-01A | | 379 | Alive | | <=65 | | male | | white | | G3 | | M0 | | N0 | | | T1 | | stage I |
| TCGA-EQ-8122-01A | | 243 | Dead | | >65 | | female | | white | | G3 | | MX | | N1 | | | T3 | | stage II |
| TCGA-D7-6526-01A | | 523 | Alive | | >65 | | female | | white | | G2 | | M0 | | N2 | | | T3 | | stage III |
| TCGA-HU-A4GY-01A | | 8 | Alive | | >65 | | female | | asian | | G3 | | M0 | | N1 | | | T4 | | stage III |
| TCGA-CG-4441-01A | | 426 | Dead | | >65 | | male | | not reported | | G2 | | M0 | | N2 | | | T2 | | stage III |
| TCGA-CD-A4MI-01A | | 358 | Dead | | <=65 | | male | | asian | | G1 | | M0 | | N1 | | | T3 | | stage III |
| TCGA-D7-6519-01A | | 625 | Alive | | <=65 | | female | | white | | G3 | | M0 | | N1 | | | T2 | | not reported |
| TCGA-CG-5722-01A | | 30 | Alive | | >65 | | female | | not reported | | G3 | | M1 | | N2 | | | T3 | | stage IV |
| TCGA-BR-8371-01A | | 359 | Dead | | <=65 | | male | | white | | G3 | | M0 | | N3 | | | T3 | | stage III |
| TCGA-CD-5799-01A | | 396 | Alive | | <=65 | | male | | asian | | G3 | | M0 | | N1 | | | T2 | | stage II |
| TCGA-HU-A4GF-01A | | 785 | Alive | | >65 | | male | | asian | | G2 | | M0 | | N0 | | | T3 | | stage II |
| TCGA-EQ-A4SO-01A | | 494 | Alive | | >65 | | male | | white | | G2 | | MX | | N1 | | | T4 | | stage III |
| TCGA-B7-A5TI-01A | | 595 | Alive | | <=65 | | male | | white | | G3 | | M0 | | N3 | | | T4 | | stage III |
| TCGA-D7-A6EY-01A | | 348 | Dead | | >65 | | female | | white | | G3 | | M0 | | N3 | | | T3 | | stage III |
| TCGA-RD-A8N9-01A | | 1083 | Alive | | <=65 | | female | | asian | | G3 | | M0 | | N1 | | | T2 | | stage II |
| TCGA-KB-A6F5-01A | | 169 | Alive | | >65 | | male | | not reported | | G3 | | M0 | | N2 | | | T3 | | stage III |
| TCGA-BR-6706-01A | | 549 | Dead | | <=65 | | male | | white | | G3 | | M0 | | N1 | | | T3 | | stage III |
| TCGA-VQ-A91U-01A | | 52 | Dead | | >65 | | male | | asian | | G2 | | M0 | | N1 | | | T3 | | stage III |
| TCGA-BR-A4IU-01A | | 838 | Alive | | <=65 | | female | | asian | | G3 | | M0 | | N1 | | | T4 | | stage III |
| TCGA-IN-A6RP-01A | | 196 | Alive | | <=65 | | male | | white | | G3 | | M0 | | N3 | | | T1 | | not reported |
| TCGA-RD-A8N4-01A | | 2171 | Alive | | <=65 | | female | | white | | G3 | | M0 | | N1 | | | T3 | | stage III |
| TCGA-CG-5733-01A | | 641 | Dead | | >65 | | female | | not reported | | G3 | | M0 | | N2 | | | T2 | | stage III |
| TCGA-D7-A6EX-01A | | 344 | Alive | | >65 | | female | | white | | G3 | | M0 | | N2 | | | T3 | | stage III |
| TCGA-CD-8528-01A | | 375 | Alive | | <=65 | | female | | asian | | G3 | | M0 | | N0 | | | T4 | | stage III |
| TCGA-RD-A8N0-01A | | 1236 | Alive | | <=65 | | female | | white | | G3 | | M0 | | N2 | | | T3 | | stage III |
| TCGA-VQ-A8P2-01A | | 1160 | Alive | | >65 | | male | | white | | G2 | | M0 | | N1 | | | T4 | | stage III |
| TCGA-VQ-A91S-01A | | 1000 | Alive | | <=65 | | male | | black | | GX | | M0 | | N1 | | | T4 | | stage III |
| TCGA-BR-4191-01A | | 558 | Dead | | >65 | | male | | white | | G2 | | M0 | | N1 | | | TX | | not reported |
| TCGA-FP-7735-01A | | 106 | Dead | | >65 | | male | | black | | G2 | | MX | | N0 | | | T2 | | stage I |
| TCGA-HU-A4H3-01A | | 882 | Alive | | <=65 | | female | | asian | | G3 | | M0 | | N3 | | | T4 | | stage III |
| TCGA-BR-7704-01A | | 1072 | Alive | | >65 | | female | | white | | G3 | | M0 | | N0 | | | T3 | | stage II |
| TCGA-CD-5798-01A | | 408 | Alive | | >65 | | male | | asian | | G2 | | M0 | | N0 | | | T3 | | stage II |
| TCGA-CG-5725-01A | | 457 | Dead | | >65 | | male | | not reported | | G3 | | M0 | | N0 | | | T2 | | stage I |
| TCGA-BR-8484-01A | | 766 | Dead | | <=65 | | male | | white | | G2 | | M0 | | N1 | | | T4 | | stage III |
| TCGA-BR-4188-01A | | 226 | Dead | | <=65 | | female | | white | | G3 | | M0 | | N1 | | | TX | | not reported |
| TCGA-BR-A4IY-01A | | 392 | Alive | | <=65 | | male | | asian | | G3 | | M0 | | N0 | | | T4 | | stage II |
| TCGA-BR-A4J4-01A | | 16 | Alive | | <=65 | | male | | asian | | G3 | | M0 | | N2 | | | T4 | | stage III |
| TCGA-VQ-AA6I-01A | | 491 | Dead | | >65 | | male | | white | | G3 | | M0 | | N3 | | | T3 | | stage III |
| TCGA-VQ-A91D-01A | | 356 | Dead | | >65 | | male | | white | | G2 | | M0 | | N2 | | | T4 | | stage III |
| TCGA-D7-8572-01A | | 511 | Alive | | <=65 | | male | | white | | G2 | | M0 | | N2 | | | T2 | | stage II |
| TCGA-B7-A5TN-01A | | 287 | Alive | | <=65 | | male | | white | | G2 | | M0 | | NX | | | T4 | | stage II |
| TCGA-D7-6518-01A | | 582 | Alive | | >65 | | male | | white | | G3 | | M0 | | N3 | | | T2 | | stage III |
| TCGA-CG-4305-01A | | 485 | Alive | | >65 | | male | | not reported | | G3 | | M0 | | N1 | | | T2 | | stage II |
| TCGA-RD-A7C1-01A | | 507 | Dead | | >65 | | male | | white | | G3 | | M0 | | N0 | | | T2 | | stage I |
| TCGA-VQ-A8PQ-01A | | 476 | Dead | | <=65 | | female | | white | | G2 | | M1 | | N1 | | | T4 | | stage IV |
| TCGA-BR-8368-01A | | 131 | Alive | | >65 | | female | | white | | G3 | | M0 | | N0 | | | T2 | | stage I |
| TCGA-CG-4436-01A | | 243 | Alive | | <=65 | | male | | not reported | | G2 | | M0 | | N0 | | | T2 | | stage I |
| TCGA-SW-A7EB-01A | | 176 | Alive | | <=65 | | male | | white | | G2 | | M0 | | N2 | | | T3 | | stage III |
| TCGA-D7-8576-01A | | 446 | Dead | | <=65 | | female | | white | | G3 | | M0 | | N3 | | | T3 | | stage III |
| TCGA-VQ-A94O-01A | | 640 | Dead | | >65 | | male | | black | | G2 | | M0 | | N3 | | | T4 | | stage III |
| TCGA-EQ-5647-01A | | 19 | Dead | | >65 | | female | | white | | G2 | | M1 | | N3 | | | T4 | | stage IV |
| TCGA-F1-6875-01A | | 2197 | Dead | | >65 | | male | | black | | G2 | | M0 | | N0 | | | T2 | | stage I |
| TCGA-VQ-A8PC-01A | | 1407 | Dead | | <=65 | | male | | white | | G3 | | M0 | | N1 | | | T3 | | stage III |
| TCGA-HF-7132-01A | | 2351 | Alive | | <=65 | | male | | not reported | | G2 | | M0 | | N1 | | | T2 | | stage II |
| TCGA-R5-A7ZE-01B | | 554 | Dead | | >65 | | female | | white | | G2 | | M0 | | N2 | | | T3 | | stage III |
| TCGA-BR-A4J7-01A | | 989 | Alive | | <=65 | | male | | asian | | G3 | | M0 | | NX | | | T4 | | stage II |
| TCGA-HU-A4G8-01A | | 690 | Alive | | >65 | | female | | asian | | G3 | | M0 | | N1 | | | T3 | | stage II |
| TCGA-VQ-A8PS-01A | | 406 | Dead | | >65 | | male | | white | | G2 | | M0 | | N1 | | | T3 | | stage III |
| TCGA-HU-A4H4-01A | | 725 | Alive | | <=65 | | female | | asian | | G3 | | M0 | | N2 | | | T2 | | stage II |
| TCGA-HU-A4GX-01A | | 616 | Alive | | >65 | | female | | asian | | G3 | | M0 | | N2 | | | T4 | | stage III |
| TCGA-VQ-A922-01A | | 275 | Dead | | >65 | | male | | not reported | | G2 | | M0 | | N1 | | | T4 | | stage IV |
| TCGA-VQ-A8P8-01A | | 942 | Alive | | >65 | | female | | black | | G2 | | M0 | | N0 | | | T4 | | stage II |
| TCGA-CG-4438-01A | | 1645 | Alive | | <=65 | | male | | not reported | | G3 | | M0 | | N2 | | | T4 | | stage IV |
| TCGA-HU-8602-01A | | 679 | Alive | | <=65 | | female | | asian | | G3 | | M0 | | N0 | | | T4 | | stage II |
| TCGA-BR-A4CQ-01A | | 1064 | Alive | | <=65 | | male | | white | | G3 | | M0 | | N1 | | | T4 | | stage III |
| TCGA-VQ-A8DU-01A | | 166 | Dead | | <=65 | | male | | white | | G2 | | M0 | | N2 | | | T3 | | stage III |
| TCGA-BR-8363-01A | | 8 | Dead | | >65 | | female | | white | | G3 | | M0 | | N0 | | | T2 | | stage I |
| TCGA-CD-A4MG-01A | | 200 | Dead | | >65 | | male | | asian | | G3 | | M0 | | N0 | | | T3 | | stage II |
| TCGA-CG-5717-01A | | 212 | Dead | | <=65 | | male | | not reported | | G3 | | M0 | | N1 | | | T2 | | stage II |
| TCGA-BR-4253-01A | | 124 | Dead | | >65 | | female | | white | | G3 | | M0 | | N1 | | | T3 | | stage III |
| TCGA-VQ-A8PF-01A | | 76 | Dead | | >65 | | male | | not reported | | G3 | | M0 | | N3 | | | T3 | | stage III |
| TCGA-BR-8384-01A | | 113 | Alive | | >65 | | male | | white | | G3 | | M0 | | N2 | | | T4 | | stage III |
| TCGA-SW-A7EA-01A | | 579 | Alive | | <=65 | | female | | white | | GX | | M0 | | N0 | | | T2 | | stage I |
| TCGA-MX-A5UJ-01A | | 600 | Alive | | >65 | | female | | white | | G3 | | M0 | | N2 | | | T3 | | stage III |
| TCGA-B7-A5TJ-01A | | 335 | Alive | | >65 | | male | | white | | G1 | | M0 | | NX | | | T4 | | stage II |
| TCGA-KB-A93J-01A | | 1124 | Alive | | >65 | | male | | white | | G3 | | M0 | | N1 | | | T2 | | stage II |
| TCGA-CD-8524-01A | | 388 | Alive | | <=65 | | female | | asian | | G3 | | M0 | | N0 | | | T3 | | stage II |
| TCGA-BR-8588-01A | | 389 | Alive | | <=65 | | female | | white | | G3 | | M0 | | N0 | | | T4 | | stage II |
| TCGA-VQ-A8E3-01A | | 661 | Dead | | >65 | | male | | white | | G3 | | M0 | | N0 | | | T3 | | stage II |
| TCGA-D7-8575-01A | | 554 | Dead | | >65 | | male | | white | | G3 | | M0 | | N2 | | | T3 | | stage III |
| TCGA-HU-A4G9-01A | | 736 | Alive | | >65 | | female | | asian | | G2 | | M0 | | N0 | | | T1 | | stage I |
| TCGA-BR-8361-01A | | 946 | Alive | | >65 | | female | | white | | G3 | | M0 | | N2 | | | T4 | | stage III |
| TCGA-HU-8245-01A | | 552 | Alive | | >65 | | male | | asian | | G3 | | M0 | | N0 | | | T3 | | stage II |
| TCGA-CD-8532-01A | | 354 | Dead | | <=65 | | male | | asian | | G2 | | M0 | | N0 | | | T3 | | stage II |
| TCGA-VQ-A928-01A | | 174 | Dead | | <=65 | | male | | white | | G2 | | M0 | | N3 | | | T3 | | stage IV |
| TCGA-D7-A6F0-01A | | 678 | Alive | | >65 | | female | | white | | G3 | | M0 | | N0 | | | T2 | | stage I |
| TCGA-CG-5719-01A | | 31 | Alive | | <=65 | | female | | not reported | | G1 | | M1 | | N0 | | | T4 | | stage IV |
| TCGA-BR-7901-01A | | 105 | Dead | | >65 | | male | | white | | G2 | | M0 | | N1 | | | T3 | | stage II |
| TCGA-FP-A4BF-01A | | 168 | Dead | | >65 | | male | | white | | G3 | | M0 | | N2 | | | T3 | | stage III |
| TCGA-BR-A452-01A | | 229 | Dead | | <=65 | | male | | asian | | G3 | | M0 | | N1 | | | T4 | | stage III |
| TCGA-CD-8535-01A | | 390 | Alive | | <=65 | | male | | asian | | G3 | | M0 | | N1 | | | T3 | | stage III |
| TCGA-VQ-A8DT-01A | | 1484 | Alive | | <=65 | | male | | not reported | | G3 | | M0 | | N3 | | | T3 | | stage III |
| TCGA-BR-6458-01A | | 588 | Dead | | <=65 | | female | | white | | G3 | | M0 | | N1 | | | T3 | | stage II |
| TCGA-HU-A4G2-01A | | 739 | Alive | | <=65 | | male | | asian | | G3 | | M0 | | N1 | | | T3 | | stage II |
| TCGA-VQ-A91V-01A | | 1297 | Alive | | <=65 | | male | | not reported | | G2 | | M0 | | N2 | | | T3 | | stage III |
| TCGA-HU-A4GP-01A | | 273 | Alive | | <=65 | | female | | asian | | G2 | | M0 | | N1 | | | T2 | | stage II |
| TCGA-CG-4443-01A | | 912 | Alive | | >65 | | male | | not reported | | G2 | | M0 | | N0 | | | T1 | | stage I |
| TCGA-BR-A4J6-01A | | 20 | Alive | | >65 | | female | | white | | G2 | | M0 | | N0 | | | T3 | | stage II |
| TCGA-BR-8080-01A | | 292 | Dead | | >65 | | female | | white | | G3 | | M0 | | N3 | | | T4 | | stage III |
| TCGA-BR-A4J9-01A | | 14 | Alive | | <=65 | | male | | white | | G3 | | M0 | | N0 | | | T3 | | stage II |
| TCGA-HJ-7597-01A | | 805 | Dead | | >65 | | female | | white | | G3 | | MX | | N0 | | | T2 | | stage I |
| TCGA-BR-8369-01A | | 427 | Alive | | >65 | | female | | white | | G3 | | M0 | | N3 | | | T3 | | stage III |
| TCGA-VQ-A8PY-01A | | 436 | Dead | | <=65 | | female | | white | | G2 | | M0 | | N2 | | | T3 | | stage III |
| TCGA-IN-A6RR-01A | | 205 | Dead | | >65 | | male | | white | | G3 | | M0 | | N1 | | | T3 | | not reported |
| TCGA-VQ-A8E7-01B | | 1138 | Alive | | <=65 | | male | | white | | G2 | | M1 | | N1 | | | T3 | | stage IV |
| TCGA-VQ-AA6F-01A | | 1646 | Alive | | <=65 | | male | | white | | G2 | | M0 | | N1 | | | T3 | | stage II |
| TCGA-BR-8370-01A | | 101 | Alive | | <=65 | | male | | white | | G3 | | M0 | | N3 | | | T3 | | stage III |
| TCGA-BR-7957-01A | | 276 | Dead | | <=65 | | female | | white | | GX | | M1 | | N3 | | | T3 | | stage IV |
| TCGA-RD-A8N5-01A | | 1747 | Dead | | >65 | | male | | white | | G2 | | M0 | | N1 | | | T3 | | stage III |
| TCGA-B7-5816-01A | | 812 | Alive | | <=65 | | female | | white | | G3 | | M0 | | N0 | | | T4 | | stage II |
| TCGA-BR-4257-01A | | 294 | Dead | | >65 | | female | | white | | G2 | | M0 | | NX | | | TX | | not reported |
| TCGA-VQ-AA69-01A | | 864 | Alive | | <=65 | | male | | white | | G2 | | M0 | | N2 | | | T3 | | stage III |
| TCGA-BR-8077-01A | | 21 | Alive | | <=65 | | female | | white | | G2 | | M0 | | N1 | | | T4 | | stage III |
| TCGA-VQ-A94T-01A | | 342 | Dead | | >65 | | male | | not reported | | G3 | | M0 | | N2 | | | T3 | | stage III |
| TCGA-BR-7722-01A | | 466 | Dead | | <=65 | | male | | white | | G3 | | M0 | | N1 | | | T3 | | stage II |
| TCGA-CD-5801-01A | | 401 | Dead | | >65 | | male | | asian | | G3 | | M0 | | N1 | | | T3 | | stage III |
| TCGA-CG-5721-01A | | 183 | Alive | | <=65 | | male | | not reported | | G3 | | M0 | | N1 | | | T4 | | stage IV |
| TCGA-MX-A666-01A | | 427 | Alive | | <=65 | | male | | white | | G2 | | MX | | NX | | | T2 | | stage II |
| TCGA-BR-8690-01A | | 325 | Alive | | <=65 | | female | | white | | G2 | | M0 | | N3 | | | T3 | | stage III |
| TCGA-BR-7723-01A | | 874 | Dead | | <=65 | | male | | white | | G3 | | M0 | | N3 | | | T3 | | stage III |
| TCGA-D7-A6ET-01A | | 804 | Alive | | >65 | | male | | white | | G2 | | M0 | | N3 | | | T3 | | stage III |
| TCGA-VQ-A91E-01A | | 664 | Alive | | >65 | | female | | white | | G3 | | M0 | | N0 | | | T4 | | stage III |
| TCGA-VQ-A91A-01A | | 1200 | Alive | | >65 | | male | | white | | G1 | | M0 | | N3 | | | T3 | | stage III |
| TCGA-R5-A7ZR-01A | | 185 | Dead | | >65 | | female | | black | | G2 | | M0 | | NX | | | T3 | | stage III |
| TCGA-VQ-A8PE-01A | | 675 | Dead | | >65 | | male | | white | | G3 | | M0 | | N3 | | | T3 | | stage III |
| TCGA-IN-A6RS-01A | | 383 | Alive | | >65 | | male | | white | | G2 | | M0 | | N0 | | | T1 | | stage I |
| TCGA-KB-A93H-01A | | 1145 | Alive | | >65 | | female | | asian | | G1 | | M0 | | N1 | | | T3 | | stage II |
| TCGA-D7-6528-01A | | 463 | Alive | | >65 | | female | | white | | G2 | | M0 | | N0 | | | T2 | | stage I |
| TCGA-VQ-AA6G-01A | | 792 | Dead | | >65 | | male | | white | | G2 | | M0 | | N0 | | | T3 | | stage II |
| TCGA-CG-5718-01A | | 1095 | Dead | | >65 | | female | | not reported | | G2 | | M0 | | N1 | | | T2 | | stage II |
| TCGA-IN-7806-01A | | 1106 | Alive | | <=65 | | male | | white | | G3 | | M0 | | N1 | | | T3 | | stage II |
| TCGA-BR-8485-01A | | 280 | Alive | | >65 | | female | | white | | G3 | | M0 | | N3 | | | T4 | | stage III |
| TCGA-BR-8680-01A | | 972 | Alive | | <=65 | | male | | asian | | G2 | | M1 | | N2 | | | T4 | | stage IV |
| TCGA-HU-A4HB-01A | | 477 | Dead | | >65 | | male | | asian | | G3 | | M0 | | N2 | | | T2 | | stage II |
| TCGA-VQ-A8PO-01A | | 282 | Dead | | >65 | | male | | white | | G3 | | M0 | | N0 | | | T4 | | stage II |
| TCGA-BR-8372-01A | | 951 | Alive | | <=65 | | male | | white | | G3 | | M0 | | N3 | | | T4 | | stage III |
| TCGA-BR-8487-01A | | 34 | Alive | | <=65 | | female | | white | | G3 | | M0 | | N0 | | | T3 | | stage II |
| TCGA-BR-A4PF-01A | | 35 | Alive | | >65 | | male | | white | | G3 | | M0 | | N2 | | | T4 | | stage III |
| TCGA-RD-A8NB-01A | | 513 | Dead | | >65 | | female | | white | | G3 | | M0 | | N1 | | | T3 | | stage III |
| TCGA-D7-8578-01A | | 643 | Alive | | >65 | | male | | white | | G2 | | M0 | | N0 | | | T2 | | stage I |
| TCGA-VQ-A8PD-01A | | 496 | Dead | | >65 | | male | | white | | G2 | | M0 | | N3 | | | T4 | | stage III |
| TCGA-VQ-A8PZ-01A | | 2233 | Alive | | <=65 | | female | | white | | G3 | | M0 | | N0 | | | T3 | | stage II |
| TCGA-VQ-A92D-01A | | 2032 | Alive | | >65 | | male | | white | | G2 | | M0 | | N0 | | | T2 | | stage I |
| TCGA-BR-8590-01A | | 284 | Dead | | <=65 | | male | | white | | G3 | | M0 | | N2 | | | T4 | | stage III |
| TCGA-VQ-A8DZ-01A | | 396 | Dead | | >65 | | male | | black | | G3 | | M1 | | N3 | | | T4 | | stage IV |
| TCGA-CD-5813-01A | | 377 | Dead | | <=65 | | male | | asian | | G3 | | M0 | | N0 | | | T3 | | stage II |
| TCGA-BR-8297-01A | | 225 | Alive | | <=65 | | male | | white | | GX | | M0 | | N3 | | | T4 | | stage III |
| TCGA-RD-A8N2-01A | | 3540 | Alive | | <=65 | | female | | white | | G3 | | M0 | | N0 | | | T2 | | stage I |
| TCGA-BR-8285-01A | | 17 | Dead | | <=65 | | female | | white | | G3 | | M0 | | N3 | | | T4 | | stage III |
| TCGA-BR-8373-01A | | 450 | Alive | | <=65 | | female | | white | | G2 | | M0 | | N1 | | | T4 | | stage III |
| TCGA-FP-7829-01A | | 594 | Alive | | >65 | | male | | white | | G2 | | M0 | | N1 | | | T3 | | stage II |
| TCGA-BR-4201-01A | | 940 | Dead | | >65 | | female | | white | | G1 | | M0 | | NX | | | T2 | | not reported |
| TCGA-VQ-A8P3-01A | | 1132 | Alive | | >65 | | male | | white | | G2 | | M0 | | N1 | | | T4 | | stage III |
| TCGA-RD-A7BS-01A | | 336 | Dead | | <=65 | | male | | white | | G3 | | M0 | | N1 | | | T3 | | stage III |
| TCGA-BR-6802-01A | | 940 | Alive | | <=65 | | male | | white | | G3 | | M0 | | N2 | | | T3 | | stage III |
| TCGA-BR-6801-01A | | 1223 | Alive | | >65 | | male | | white | | G2 | | M0 | | N0 | | | T3 | | stage II |
| TCGA-D7-A6EV-01A | | 342 | Alive | | >65 | | female | | white | | G3 | | M0 | | N2 | | | T2 | | stage II |
| TCGA-CG-4440-01A | | 122 | Dead | | >65 | | female | | not reported | | G3 | | M1 | | N3 | | | T3 | | stage IV |
| TCGA-IN-A7NR-01A | | 198 | Alive | | <=65 | | female | | white | | G3 | | M1 | | N3 | | | T3 | | stage IV |
| TCGA-D7-A4YV-01A | | 180 | Dead | | >65 | | female | | white | | G3 | | M0 | | N1 | | | T3 | | stage II |
| TCGA-VQ-A8PP-01A | | 712 | Dead | | >65 | | male | | not reported | | G2 | | M0 | | N1 | | | T4 | | stage IV |
| TCGA-BR-A4QI-01A | | 652 | Dead | | >65 | | female | | white | | G3 | | M0 | | N0 | | | T3 | | stage II |
| TCGA-VQ-A8P5-01A | | 235 | Dead | | >65 | | male | | white | | G3 | | M0 | | N0 | | | T3 | | stage II |
| TCGA-ZA-A8F6-01A | | 525 | Alive | | >65 | | male | | white | | G2 | | MX | | N0 | | | T2 | | stage I |
| TCGA-BR-8382-01A | | 762 | Dead | | >65 | | female | | white | | G3 | | M0 | | N3 | | | T4 | | stage III |
| TCGA-BR-6457-01A | | 416 | Alive | | >65 | | male | | white | | G3 | | M0 | | N0 | | | T3 | | stage II |
| TCGA-IN-A6RI-01A | | 559 | Alive | | <=65 | | male | | white | | G2 | | M0 | | N0 | | | T1 | | stage I |
| TCGA-BR-8060-01A | | 348 | Dead | | >65 | | female | | white | | G3 | | M0 | | N2 | | | T2 | | stage II |
| TCGA-CD-A48A-01A | | 378 | Alive | | <=65 | | male | | asian | | G2 | | M0 | | N0 | | | T3 | | stage II |
| TCGA-CD-8525-01A | | 383 | Alive | | >65 | | female | | asian | | G3 | | M0 | | N1 | | | T3 | | stage III |
| TCGA-BR-7958-01A | | 899 | Alive | | <=65 | | male | | white | | G3 | | M0 | | N0 | | | T4 | | stage III |
| TCGA-RD-A7BT-01A | | 262 | Dead | | >65 | | male | | black | | G3 | | M0 | | N3 | | | T3 | | stage IV |
| TCGA-BR-6709-01A | | 370 | Dead | | <=65 | | female | | white | | G3 | | M0 | | N3 | | | T3 | | stage III |
| TCGA-BR-6803-01A | | 949 | Alive | | <=65 | | female | | white | | G3 | | M0 | | N0 | | | T3 | | stage II |
| TCGA-BR-7716-01A | | 1210 | Alive | | <=65 | | female | | white | | G2 | | M0 | | N1 | | | T3 | | stage II |
| TCGA-CG-4460-01A | | 669 | Dead | | >65 | | female | | not reported | | G2 | | M1 | | N1 | | | T4 | | stage IV |
| TCGA-VQ-A94R-01A | | 1294 | Dead | | <=65 | | male | | white | | G2 | | M0 | | N2 | | | T4 | | not reported |
| TCGA-CG-4472-01A | | 365 | Alive | | <=65 | | male | | not reported | | G3 | | M0 | | N1 | | | T4 | | stage IV |
| TCGA-D7-6521-01A | | 564 | Alive | | <=65 | | male | | white | | G3 | | M0 | | N2 | | | T2 | | not reported |
| TCGA-BR-8589-01A | | 825 | Alive | | <=65 | | male | | white | | G3 | | M0 | | N1 | | | T4 | | stage III |
| TCGA-BR-8483-01A | | 164 | Alive | | <=65 | | male | | white | | G2 | | M0 | | N2 | | | T3 | | stage III |
| TCGA-VQ-A91W-01A | | 1851 | Alive | | <=65 | | male | | white | | G2 | | M0 | | N1 | | | T3 | | stage III |
| TCGA-BR-4255-01A | | 124 | Dead | | >65 | | female | | white | | G3 | | M0 | | N1 | | | T3 | | stage III |
| TCGA-VQ-A8PK-01A | | 543 | Dead | | <=65 | | male | | white | | G2 | | M0 | | N3 | | | T3 | | stage III |
| TCGA-FP-8211-01A | | 413 | Alive | | <=65 | | male | | white | | G2 | | MX | | N1 | | | T3 | | stage II |
| TCGA-D7-8570-01A | | 752 | Alive | | <=65 | | male | | white | | G3 | | M0 | | N3 | | | T3 | | stage III |
| TCGA-HF-A5NB-01A | | 928 | Alive | | >65 | | female | | not reported | | G3 | | M0 | | N3 | | | T4 | | stage III |
| TCGA-BR-8296-01A | | 474 | Dead | | <=65 | | female | | white | | G3 | | M0 | | N2 | | | T4 | | stage III |
| TCGA-BR-8364-01A | | 675 | Alive | | <=65 | | female | | white | | G3 | | M0 | | N2 | | | T4 | | stage III |
| TCGA-CD-8531-01A | | 383 | Alive | | >65 | | female | | asian | | G3 | | M0 | | N1 | | | T3 | | stage III |
| TCGA-BR-6453-01A | | 485 | Alive | | <=65 | | male | | white | | G3 | | M0 | | N1 | | | T2 | | stage II |
| TCGA-VQ-AA6A-01A | | 1184 | Alive | | <=65 | | male | | white | | G2 | | M0 | | N3 | | | T4 | | stage III |
| TCGA-D7-6815-01A | | 486 | Alive | | >65 | | female | | white | | G2 | | M0 | | N2 | | | T2 | | stage II |
| TCGA-VQ-A8PM-01A | | 57 | Dead | | <=65 | | male | | white | | G3 | | M0 | | N3 | | | T4 | | stage IV |
| TCGA-F1-A448-01A | | 647 | Alive | | >65 | | male | | white | | G3 | | M0 | | N3 | | | T3 | | stage III |
| TCGA-VQ-A924-01A | | 1686 | Dead | | >65 | | male | | not reported | | G2 | | M0 | | N0 | | | T3 | | stage II |
| TCGA-D7-8574-01A | | 523 | Alive | | >65 | | male | | white | | G3 | | M0 | | N3 | | | T2 | | stage III |
| TCGA-BR-A4J5-01A | | 862 | Alive | | <=65 | | male | | asian | | G3 | | M0 | | N1 | | | T4 | | stage III |
| TCGA-CG-4449-01A | | 580 | Alive | | >65 | | male | | not reported | | G2 | | M0 | | N1 | | | T2 | | stage II |
| TCGA-D7-A4YT-01A | | 434 | Alive | | <=65 | | male | | white | | G2 | | M0 | | N3 | | | T2 | | stage III |
| TCGA-VQ-A91X-01A | | 289 | Dead | | >65 | | male | | white | | G3 | | M0 | | N2 | | | T3 | | stage III |
| TCGA-RD-A7BW-01A | | 156 | Dead | | >65 | | female | | white | | G3 | | M0 | | N0 | | | T2 | | stage I |
| TCGA-HU-A4H2-01A | | 394 | Alive | | <=65 | | female | | asian | | G2 | | M0 | | N3 | | | T3 | | stage III |
| TCGA-CD-8529-01A | | 374 | Alive | | <=65 | | male | | asian | | G2 | | M1 | | N0 | | | T4 | | stage IV |
| TCGA-HU-A4G6-01A | | 738 | Alive | | >65 | | male | | asian | | G3 | | M0 | | N0 | | | T1 | | stage I |
| TCGA-BR-7707-01A | | 1090 | Alive | | >65 | | female | | white | | G3 | | M0 | | N0 | | | T2 | | stage I |
| TCGA-CD-A486-01A | | 192 | Dead | | >65 | | male | | asian | | G2 | | M0 | | N0 | | | T3 | | stage II |
| TCGA-CD-A4MH-01A | | 371 | Alive | | >65 | | female | | asian | | G2 | | M0 | | N0 | | | T3 | | stage II |
| TCGA-KB-A6F7-01A | | 1935 | Alive | | <=65 | | female | | not reported | | G3 | | M0 | | N1 | | | T1 | | stage I |
| TCGA-FP-7998-01A | | 678 | Alive | | >65 | | male | | white | | G3 | | MX | | N3 | | | T4 | | stage III |
| TCGA-BR-6563-01A | | 1190 | Alive | | <=65 | | male | | white | | G3 | | M0 | | N1 | | | T3 | | stage II |
| TCGA-D7-A4YY-01A | | 419 | Alive | | <=65 | | male | | white | | G3 | | M0 | | N3 | | | T3 | | stage III |
| TCGA-BR-8687-01A | | 250 | Dead | | >65 | | female | | white | | G3 | | M0 | | N2 | | | T4 | | stage III |
| TCGA-BR-6705-01A | | 779 | Dead | | >65 | | female | | white | | G3 | | M0 | | N3 | | | T3 | | stage III |
| TCGA-BR-8360-01A | | 188 | Alive | | >65 | | male | | white | | G2 | | M0 | | N0 | | | T3 | | stage II |
| TCGA-D7-5578-01A | | 385 | Alive | | >65 | | male | | white | | G3 | | M0 | | N2 | | | T3 | | stage III |
| TCGA-BR-7703-01A | | 1100 | Alive | | >65 | | male | | white | | G2 | | M0 | | N0 | | | T1 | | stage I |
| TCGA-VQ-A91Q-01A | | 633 | Dead | | <=65 | | male | | not reported | | G3 | | M0 | | N3 | | | T3 | | stage IV |
| TCGA-VQ-A925-01A | | 138 | Dead | | >65 | | male | | white | | G2 | | M0 | | N2 | | | T3 | | stage III |
| TCGA-HU-A4GQ-01A | | 3 | Dead | | >65 | | male | | asian | | G2 | | M0 | | N2 | | | T4 | | stage III |
| TCGA-CD-8530-01A | | 377 | Alive | | <=65 | | male | | asian | | G3 | | M0 | | N0 | | | T3 | | stage II |
| TCGA-HU-A4GT-01A | | 198 | Alive | | >65 | | female | | asian | | G3 | | M0 | | N0 | | | T3 | | stage II |
| TCGA-HU-A4GD-01A | | 692 | Alive | | <=65 | | male | | asian | | G3 | | M0 | | N1 | | | T3 | | stage II |
| TCGA-CG-5727-01A | | 2405 | Alive | | >65 | | male | | not reported | | G3 | | M0 | | N0 | | | T3 | | stage II |
| TCGA-BR-6455-01A | | 422 | Dead | | <=65 | | male | | white | | G3 | | M0 | | N1 | | | T3 | | stage II |
| TCGA-FP-A9TM-01A | | 189 | Alive | | >65 | | male | | white | | G2 | | M0 | | N1 | | | T1 | | not reported |
| TCGA-VQ-A8PH-01A | | 389 | Dead | | <=65 | | male | | white | | G2 | | M0 | | N3 | | | T3 | | stage III |
| TCGA-CG-4306-01A | | 1 | Dead | | >65 | | male | | not reported | | G3 | | M1 | | N2 | | | T3 | | stage IV |
| TCGA-CD-8534-01A | | 367 | Alive | | <=65 | | male | | asian | | G3 | | M0 | | N0 | | | T3 | | stage II |
| TCGA-VQ-A91Z-01A | | 1690 | Alive | | >65 | | female | | white | | G2 | | M0 | | N1 | | | T3 | | stage III |
| TCGA-BR-6564-01A | | 794 | Dead | | <=65 | | female | | white | | G3 | | M0 | | N2 | | | T3 | | stage III |
| TCGA-BR-7715-01A | | 1023 | Alive | | <=65 | | male | | white | | G2 | | M0 | | N0 | | | T3 | | stage II |
| TCGA-D7-A4YX-01A | | 1108 | Alive | | <=65 | | male | | white | | G3 | | M0 | | N1 | | | T3 | | stage II |
| TCGA-BR-8058-01A | | 1133 | Alive | | <=65 | | female | | white | | G3 | | M0 | | N2 | | | T4 | | stage III |
| TCGA-HF-7133-01A | | 1918 | Alive | | <=65 | | female | | not reported | | G2 | | M0 | | N3 | | | T2 | | stage IV |
| TCGA-CD-8526-01A | | 381 | Alive | | >65 | | female | | asian | | G3 | | M0 | | N1 | | | T3 | | stage III |
| TCGA-BR-A4QM-01A | | 156 | Alive | | <=65 | | male | | white | | G3 | | M0 | | N2 | | | T3 | | stage III |
| TCGA-VQ-A8DL-01A | | 28 | Dead | | >65 | | female | | white | | G2 | | M0 | | N0 | | | T3 | | stage II |
| TCGA-CG-4455-01A | | 366 | Alive | | >65 | | male | | not reported | | G3 | | M0 | | N1 | | | T2 | | stage II |
| TCGA-D7-A748-01A | | 132 | Dead | | <=65 | | female | | white | | G3 | | M1 | | N3 | | | T4 | | stage IV |
| TCGA-CG-4300-01A | | 609 | Dead | | >65 | | male | | not reported | | G2 | | M0 | | N2 | | | T3 | | stage III |
| TCGA-HU-A4GU-01A | | 200 | Alive | | >65 | | male | | asian | | G3 | | M0 | | N1 | | | T3 | | stage II |
| TCGA-HU-A4H6-01A | | 644 | Alive | | >65 | | female | | asian | | G2 | | M0 | | N2 | | | T3 | | stage III |
| TCGA-R5-A804-01A | | 140 | Dead | | <=65 | | male | | black | | G3 | | M1 | | N2 | | | T3 | | stage IV |
| TCGA-D7-6524-01A | | 543 | Alive | | <=65 | | male | | white | | G3 | | M0 | | N1 | | | T2 | | stage II |
| TCGA-CD-8533-01A | | 468 | Alive | | <=65 | | male | | asian | | G3 | | M0 | | N0 | | | T3 | | stage II |
| TCGA-CG-4465-01A | | 274 | Dead | | >65 | | female | | not reported | | G3 | | M1 | | N3 | | | T4 | | stage IV |
| TCGA-FP-8099-01A | | 519 | Alive | | >65 | | male | | white | | G2 | | MX | | N0 | | | T3 | | stage II |
| TCGA-VQ-A91K-01A | | 1862 | Alive | | >65 | | male | | white | | G2 | | M0 | | N1 | | | T3 | | stage III |
| TCGA-BR-8592-01A | | 191 | Dead | | <=65 | | female | | white | | G3 | | M0 | | N1 | | | T4 | | stage III |
| TCGA-D7-6818-01A | | 376 | Dead | | <=65 | | male | | white | | G3 | | M0 | | N3 | | | T2 | | stage III |
| TCGA-D7-8573-01A | | 593 | Alive | | <=65 | | male | | white | | G3 | | M0 | | N0 | | | T3 | | stage II |
| TCGA-CG-5726-01A | | 881 | Dead | | >65 | | male | | not reported | | G2 | | M0 | | N0 | | | T1 | | stage I |
| TCGA-VQ-A8PU-01A | | 832 | Dead | | >65 | | female | | white | | G3 | | M0 | | N1 | | | T4 | | stage III |
| TCGA-BR-8284-01A | | 245 | Dead | | >65 | | female | | white | | G3 | | M0 | | N3 | | | T4 | | stage III |
| TCGA-D7-6817-01A | | 389 | Alive | | <=65 | | male | | white | | G3 | | M0 | | N3 | | | T2 | | stage III |
| TCGA-BR-4184-01A | | 212 | Dead | | >65 | | male | | white | | G2 | | MX | | N1 | | | T3 | | stage III |
| TCGA-BR-4256-01A | | 284 | Dead | | >65 | | male | | white | | G2 | | M0 | | N1 | | | TX | | not reported |
| TCGA-IN-AB1V-01A | | 479 | Alive | | <=65 | | male | | white | | G2 | | M0 | | N0 | | | T1 | | stage I |
| TCGA-FP-A4BE-01A | | 337 | Alive | | <=65 | | male | | black | | G3 | | M0 | | N0 | | | T3 | | stage II |
| TCGA-BR-8676-01A | | 229 | Alive | | <=65 | | male | | white | | G3 | | M0 | | N3 | | | T3 | | stage III |
| TCGA-BR-8295-01A | | 67 | Dead | | <=65 | | female | | white | | G3 | | M0 | | N0 | | | T4 | | stage II |
| TCGA-IN-7808-01A | | 105 | Dead | | <=65 | | male | | white | | G3 | | M1 | | N3 | | | T3 | | not reported |
| TCGA-CD-A4MJ-01A | | 384 | Alive | | <=65 | | male | | asian | | G2 | | M0 | | N0 | | | T2 | | stage I |
| TCGA-BR-7197-01A | | 280 | Alive | | >65 | | male | | white | | GX | | M0 | | N0 | | | T3 | | stage II |
| TCGA-VQ-A8PJ-01A | | 82 | Dead | | <=65 | | male | | not reported | | GX | | M1 | | N2 | | | T4 | | stage IV |
| TCGA-D7-6522-01A | | 566 | Alive | | <=65 | | male | | white | | G3 | | M0 | | N0 | | | T2 | | stage I |
| TCGA-VQ-A8PT-01A | | 900 | Alive | | <=65 | | male | | white | | G3 | | M0 | | N0 | | | T4 | | stage III |
| TCGA-CG-4469-01A | | 215 | Dead | | >65 | | male | | not reported | | G3 | | M0 | | N3 | | | T3 | | stage IV |
| TCGA-BR-6707-01A | | 605 | Dead | | >65 | | male | | white | | G3 | | M0 | | N0 | | | T3 | | stage II |
| TCGA-RD-A8N1-01A | | 3519 | Alive | | >65 | | male | | white | | G3 | | M0 | | N2 | | | T3 | | stage III |
| TCGA-HU-8608-01A | | 641 | Alive | | >65 | | male | | asian | | G3 | | M0 | | N2 | | | T4 | | stage III |
| TCGA-CD-8527-01A | | 218 | Dead | | >65 | | female | | asian | | G2 | | M0 | | N1 | | | T2 | | stage II |
| TCGA-R5-A7ZI-01A | | 2267 | Alive | | <=65 | | female | | white | | G3 | | M1 | | N1 | | | T4 | | stage IV |
| TCGA-3M-AB46-01A | | 1765 | Alive | | >65 | | male | | white | | G2 | | MX | | N0 | | | T2 | | stage I |
| TCGA-CG-5730-01A | | 122 | Alive | | >65 | | female | | not reported | | G2 | | M1 | | N1 | | | T2 | | stage IV |
| TCGA-HU-8249-01A | | 881 | Alive | | >65 | | male | | asian | | G2 | | M0 | | N2 | | | T3 | | stage III |
| TCGA-R5-A805-01A | | 281 | Dead | | >65 | | male | | white | | G3 | | M0 | | N2 | | | T3 | | stage III |
| TCGA-BR-8365-01A | | 533 | Dead | | >65 | | female | | white | | G3 | | M0 | | N0 | | | T3 | | stage II |
| TCGA-BR-A4J2-01A | | 431 | Alive | | >65 | | male | | asian | | G3 | | M0 | | N0 | | | T4 | | stage II |
| TCGA-HU-8244-01A | | 742 | Alive | | >65 | | female | | asian | | G1 | | M0 | | N0 | | | T1 | | stage I |
| TCGA-F1-6874-01A | | 440 | Alive | | >65 | | male | | white | | G3 | | M0 | | N0 | | | T2 | | stage I |
| TCGA-BR-8682-01A | | 991 | Alive | | <=65 | | male | | asian | | G3 | | M0 | | N0 | | | T4 | | stage II |
| TCGA-B7-A5TK-01A | | 288 | Alive | | <=65 | | male | | white | | G3 | | M0 | | NX | | | T4 | | stage III |
| TCGA-BR-8289-01A | | 81 | Dead | | <=65 | | male | | white | | G3 | | M1 | | N3 | | | T4 | | stage IV |
| TCGA-BR-4279-01A | | 291 | Dead | | <=65 | | male | | white | | G3 | | M0 | | N1 | | | T2 | | stage II |
| TCGA-D7-8579-01A | | 636 | Alive | | >65 | | female | | white | | G2 | | M0 | | N2 | | | T2 | | stage II |
| TCGA-BR-A4J8-01A | | 411 | Alive | | >65 | | female | | white | | G3 | | M0 | | N3 | | | T3 | | stage III |
| TCGA-VQ-A8E2-01A | | 1319 | Alive | | <=65 | | male | | asian | | G2 | | M0 | | N2 | | | T3 | | stage III |
| TCGA-CG-5728-01A | | 579 | Alive | | >65 | | female | | not reported | | G3 | | M0 | | N0 | | | T2 | | stage I |
| TCGA-BR-6566-01A | | 997 | Alive | | <=65 | | female | | white | | G3 | | M0 | | N0 | | | T3 | | stage II |
| TCGA-BR-8686-01A | | 635 | Dead | | >65 | | male | | asian | | G3 | | M0 | | N1 | | | T4 | | stage III |
| TCGA-BR-8291-01A | | 607 | Dead | | <=65 | | male | | white | | GX | | M0 | | N1 | | | T3 | | stage II |
| TCGA-IN-A7NU-01A | | 356 | Alive | | >65 | | male | | white | | G3 | | M0 | | N3 | | | T3 | | stage III |
| TCGA-VQ-A927-01A | | 200 | Dead | | >65 | | male | | white | | G1 | | M0 | | N3 | | | T3 | | stage III |
| TCGA-CD-8536-01A | | 378 | Alive | | >65 | | male | | asian | | G3 | | M0 | | N0 | | | T3 | | stage II |
| TCGA-BR-8367-01A | | 801 | Dead | | <=65 | | male | | white | | G3 | | M0 | | N3 | | | T3 | | stage III |
| TCGA-VQ-A94U-01A | | 819 | Alive | | >65 | | male | | white | | G3 | | M0 | | N0 | | | T4 | | stage II |
| TCGA-D7-5577-01A | | 782 | Dead | | <=65 | | female | | white | | G2 | | M0 | | N3 | | | T2 | | stage III |
| TCGA-CD-5802-01A | | 406 | Alive | | <=65 | | male | | asian | | G2 | | M0 | | N0 | | | T3 | | stage II |
| TCGA-BR-8286-01A | | 895 | Alive | | <=65 | | male | | white | | G3 | | M0 | | N0 | | | T3 | | stage II |
| TCGA-VQ-AA6D-01A | | 521 | Alive | | <=65 | | female | | not reported | | G2 | | M0 | | N1 | | | T4 | | stage III |
| TCGA-VQ-AA6K-01A | | 378 | Dead | | <=65 | | male | | white | | G3 | | M0 | | N3 | | | T4 | | stage III |
| TCGA-HF-7134-01A | | 1588 | Alive | | <=65 | | male | | not reported | | G2 | | M0 | | N0 | | | T1 | | stage I |
| TCGA-BR-A4PD-01A | | 628 | Alive | | >65 | | female | | white | | G3 | | M0 | | N0 | | | T4 | | stage II |
| TCGA-BR-8081-01A | | 981 | Alive | | >65 | | female | | white | | G2 | | M0 | | N0 | | | T4 | | stage II |
| TCGA-BR-6565-01A | | 279 | Dead | | >65 | | male | | white | | G2 | | M0 | | N0 | | | T4 | | stage II |
| TCGA-FP-8210-01A | | 153 | Dead | | <=65 | | male | | asian | | G3 | | M0 | | N1 | | | T3 | | stage III |
| TCGA-BR-6710-01A | | 273 | Alive | | <=65 | | male | | white | | G2 | | M0 | | N0 | | | T2 | | stage I |
| TCGA-BR-7959-01A | | 1010 | Alive | | <=65 | | male | | white | | G3 | | M0 | | N1 | | | T4 | | stage III |
| TCGA-CD-A48C-01A | | 353 | Dead | | >65 | | female | | asian | | G3 | | M0 | | N1 | | | T3 | | stage II |
| TCGA-D7-A747-01A | | 255 | Dead | | <=65 | | male | | white | | G3 | | M0 | | N1 | | | T3 | | stage II |
| TCGA-IP-7968-01A | | 77 | Alive | | >65 | | male | | white | | G2 | | MX | | N2 | | | T3 | | stage III |
| TCGA-D7-A4Z0-01A | | 449 | Alive | | <=65 | | female | | white | | G3 | | M0 | | N2 | | | T2 | | stage II |
| TCGA-BR-6452-01A | | 1055 | Alive | | >65 | | female | | white | | G3 | | M0 | | N0 | | | T3 | | stage II |
| TCGA-BR-6456-01A | | 526 | Dead | | >65 | | female | | white | | G2 | | M0 | | N1 | | | T3 | | stage II |
| TCGA-HU-A4G3-01A | | 170 | Alive | | <=65 | | male | | asian | | G2 | | M0 | | N2 | | | T2 | | stage II |
| TCGA-HU-8604-01A | | 694 | Alive | | >65 | | female | | asian | | G2 | | M0 | | N0 | | | T3 | | stage II |
| TCGA-CD-A487-01A | | 374 | Alive | | <=65 | | male | | asian | | G3 | | M0 | | N1 | | | T3 | | stage II |
| TCGA-VQ-A8PB-01A | | 1043 | Dead | | <=65 | | female | | white | | G2 | | M0 | | N0 | | | T3 | | stage II |
| TCGA-D7-6822-01A | | 375 | Alive | | >65 | | male | | white | | G2 | | M0 | | N0 | | | T2 | | stage I |
| TCGA-HU-A4GH-01A | | 358 | Alive | | >65 | | male | | asian | | G2 | | M0 | | N0 | | | T1 | | stage I |
| TCGA-RD-A8MW-01A | | 1153 | Dead | | >65 | | male | | white | | G3 | | M0 | | N2 | | | T3 | | stage III |
| TCGA-HU-A4GC-01A | | 99 | Alive | | >65 | | male | | asian | | G3 | | M0 | | N2 | | | T4 | | stage III |
| TCGA-BR-A4QL-01A | | 491 | Dead | | >65 | | female | | white | | G2 | | M0 | | N3 | | | T3 | | stage III |
| TCGA-VQ-AA64-01A | | 560 | Dead | | >65 | | male | | white | | G2 | | M0 | | N2 | | | T3 | | stage III |
| TCGA-BR-A4PE-01A | | 621 | Alive | | >65 | | female | | white | | G2 | | M0 | | N0 | | | T2 | | stage I |
| TCGA-BR-8678-01A | | 754 | Alive | | >65 | | male | | white | | G2 | | M0 | | N0 | | | T2 | | stage I |
| TCGA-CG-4444-01A | | 1431 | Alive | | >65 | | male | | not reported | | G3 | | M0 | | N2 | | | T2 | | stage III |
| TCGA-VQ-AA6J-01A | | 838 | Alive | | >65 | | male | | black | | G3 | | M0 | | N2 | | | T4 | | stage III |
| TCGA-CG-5723-01A | | 2496 | Alive | | >65 | | male | | not reported | | G3 | | M0 | | N1 | | | T2 | | stage II |
| TCGA-CD-5800-01A | | 400 | Alive | | <=65 | | female | | asian | | G1 | | M0 | | N0 | | | T3 | | stage II |
| TCGA-BR-A4CS-01A | | 45 | Dead | | >65 | | male | | white | | G3 | | M0 | | N3 | | | T4 | | stage III |
| TCGA-BR-8366-01A | | 29 | Alive | | >65 | | female | | white | | G3 | | M0 | | N0 | | | T3 | | stage II |
| TCGA-FP-A8CX-01A | | 7 | Alive | | <=65 | | male | | white | | G3 | | MX | | N3 | | | T4 | | stage III |
| TCGA-BR-8591-01A | | 856 | Alive | | >65 | | male | | white | | G3 | | M0 | | N3 | | | T4 | | stage III |
| TCGA-D7-6820-01A | | 344 | Alive | | <=65 | | male | | white | | G2 | | M0 | | N1 | | | T2 | | stage II |
| TCGA-IN-8663-01A | | 103 | Dead | | >65 | | male | | white | | G3 | | M0 | | N2 | | | T2 | | stage II |
| TCGA-BR-8381-01A | | 224 | Alive | | <=65 | | male | | white | | G3 | | M0 | | N1 | | | T3 | | stage II |
| TCGA-HU-A4GJ-01A | | 650 | Alive | | <=65 | | female | | asian | | G3 | | M0 | | N3 | | | T4 | | stage III |
| TCGA-ZQ-A9CR-01A | | 24 | Dead | | >65 | | female | | white | | G3 | | MX | | N3 | | | T4 | | stage III |
| TCGA-IN-AB1X-01A | | 411 | Alive | | >65 | | female | | white | | G3 | | M0 | | N0 | | | T3 | | stage II |
| TCGA-VQ-A8E0-01A | | 562 | Dead | | >65 | | male | | black | | G3 | | M0 | | N2 | | | T3 | | stage III |
| TCGA-HU-8243-01A | | 180 | Dead | | >65 | | male | | asian | | G2 | | M0 | | N3 | | | T4 | | stage III |
| TCGA-BR-8380-01A | | 21 | Dead | | <=65 | | male | | white | | G3 | | M0 | | N3 | | | T4 | | stage III |
| TCGA-BR-4183-01A | | 201 | Dead | | <=65 | | female | | white | | G3 | | M0 | | N1 | | | T3 | | stage III |
| TCGA-BR-8362-01A | 398 | | | Dead | | <=65 | | male | | white | | G3 | | M0 | | N3 | T4 | | stage III | |

**Table S2** The 32 myeloid state-related prognostic genes (MSRPGs).

| Gene | HR | 95%CI lower | 95%CI upper | Cox p value | KM p value | Myeloid state |
| --- | --- | --- | --- | --- | --- | --- |
| PLTP | 1.235 | 1.062 | 1.436 | 0.006089 | 1.63E-05 | State 1 |
| THBS1 | 1.187 | 1.055 | 1.336 | 0.004347 | 0.003867 | State 2 |
| DAB2 | 1.385 | 1.131 | 1.697 | 0.00164 | 0.000492 | State 1 |
| VCAN | 1.284 | 1.112 | 1.482 | 0.000639 | 9.43E-05 | State 2 |
| INHBA | 1.205 | 1.052 | 1.380 | 0.007188 | 0.00065 | State 2 |
| AKR1B1 | 1.390 | 1.150 | 1.679 | 0.000651 | 9.19E-05 | State 1 |
| SERPINF1 | 1.184 | 1.043 | 1.344 | 0.008864 | 0.00885 | State 1 |
| PLIN2 | 1.258 | 1.059 | 1.494 | 0.00889 | 0.001218 | State 1 |
| COL1A1 | 1.153 | 1.037 | 1.283 | 0.008783 | 0.006088 | State 3 |
| SPARC | 1.283 | 1.107 | 1.487 | 0.000953 | 0.001048 | State 3 |
| COL1A2 | 1.176 | 1.043 | 1.326 | 0.007977 | 0.00797 | State 3 |
| OLFML2B | 1.237 | 1.083 | 1.414 | 0.001745 | 0.000114 | State 1 |
| AXL | 1.279 | 1.084 | 1.509 | 0.00348 | 0.006335 | State 1 |
| COL3A1 | 1.170 | 1.042 | 1.313 | 0.007941 | 0.000677 | State 3 |
| SDC2 | 1.305 | 1.084 | 1.570 | 0.004831 | 0.002522 | State 2 |
| NRP1 | 1.566 | 1.248 | 1.966 | 0.000109 | 2.99E-06 | State 1 |
| ACKR3 | 1.267 | 1.061 | 1.513 | 0.008836 | 0.00046 | State 1 |
| IGFBP7 | 1.248 | 1.062 | 1.467 | 0.007226 | 0.001105 | State 3 |
| TMEM45A | 1.363 | 1.118 | 1.660 | 0.002146 | 0.000599 | State 3 |
| CD109 | 1.262 | 1.075 | 1.482 | 0.00451 | 8.78E-05 | State 1 |
| LAMP5 | 1.285 | 1.092 | 1.513 | 0.002591 | 0.008824 | State 4 |
| BGN | 1.197 | 1.059 | 1.353 | 0.004016 | 0.001088 | State 3 |
| BEX3 | 1.203 | 1.059 | 1.367 | 0.00455 | 0.000827 | State 1 |
| CXCR4 | 1.212 | 1.062 | 1.382 | 0.004372 | 0.000954 | State 3 |
| DEPP1 | 1.205 | 1.046 | 1.387 | 0.009692 | 0.002166 | State 3 |
| TFPI | 1.389 | 1.153 | 1.673 | 0.000538 | 0.00056 | State 2 |
| THBS2 | 1.146 | 1.035 | 1.269 | 0.008945 | 0.001362 | State 3 |
| NT5E | 1.312 | 1.130 | 1.522 | 0.000357 | 0.000101 | State 5 |
| LUM | 1.194 | 1.049 | 1.358 | 0.007193 | 0.007075 | State 3 |
| PLOD2 | 1.372 | 1.120 | 1.681 | 0.002301 | 0.000181 | State 3 |
| EFEMP1 | 1.168 | 1.045 | 1.306 | 0.006096 | 0.000134 | State 4 |
| NNMT | 1.228 | 1.072 | 1.408 | 0.00316 | 7.33E-05 | State 4 |

Functional theme:

state 1: Lipid handling, efferocytosis, receptor signaling, adaptive survival

state 2: Inflammatory monocyte-like program, matrix interaction, cytokine/vascular signaling

state 3: ECM remodeling, collagen organization, EMT/stromal interaction, CXCR4-linked trafficking

state 4: Regulatory/metabolic adaptation and immune-modulatory marker expression

state 5: Adenosine-related immune regulation


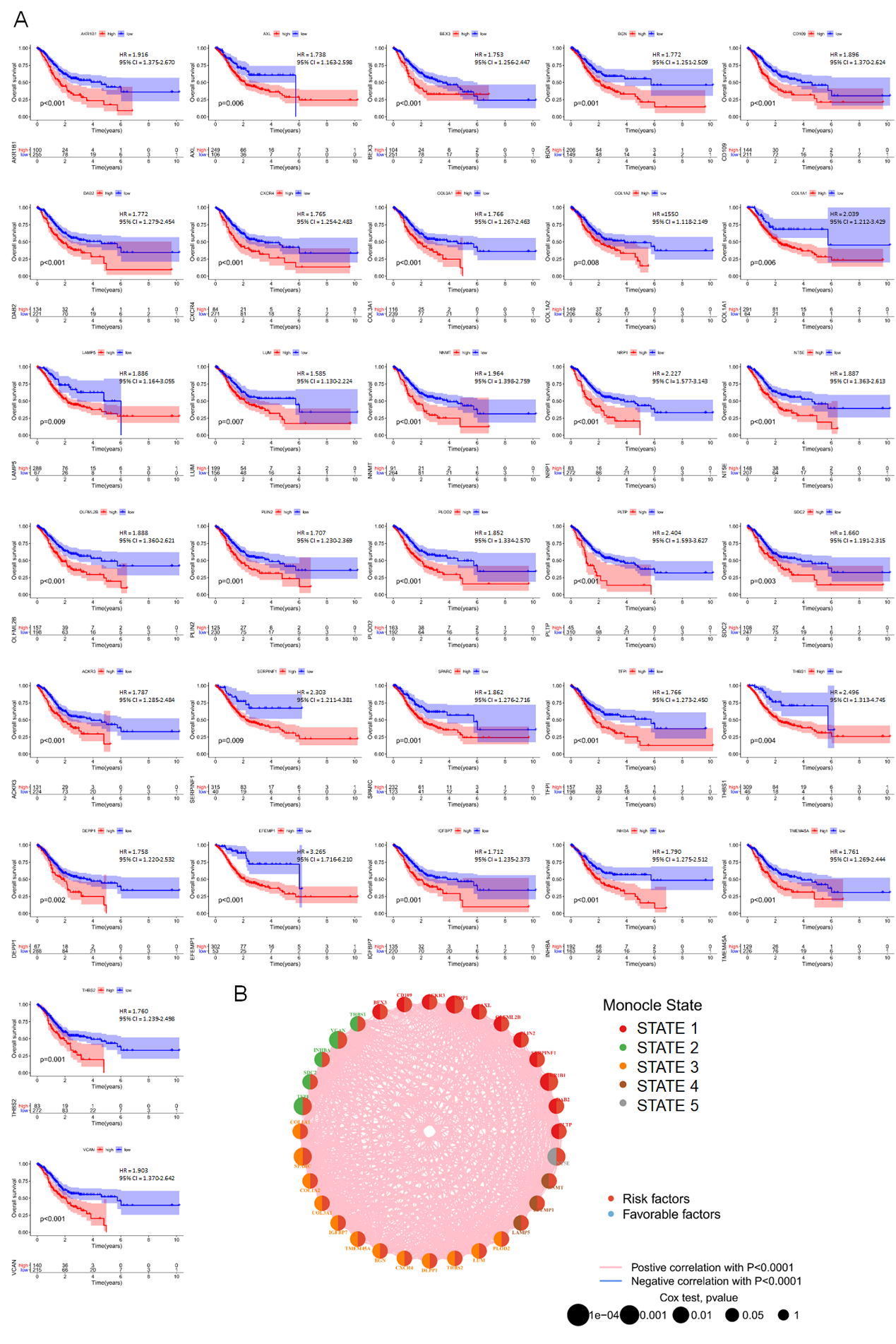


**Fig. S4** KM survival analysis and the co-expression pattern for MSRPGs

**(A)** The prognostic information of the MSRPGs were demonstrated. **(B)** We also built the co-expression network to visualize the relationship between the MSRPGs. Specifically, MSRPGs were all risk factors.

KM, Kaplan-Meier; MSRPGs, myeloid state-related prognostic genes;

**Table S3** Evaluation of Consensus Clustering Metrics Across Different Values of k

| k | PAC | mean cluster consensus | min cluster size |
| --- | --- | --- | --- |
| 2 | 0.014 | 0.993 | 200 |
| 3 | 0.175 | 0.911 | 98 |
| 4 | 0.349 | 0.822 | 64 |
| 5 | 0.318 | 0.786 | 55 |
| 6 | 0.268 | 0.774 | 38 |
| 7 | 0.225 | 0.797 | 28 |
| 8 | 0.224 | 0.741 | 15 |
| 9 | 0.194 | 0.735 | 19 |

PAC, Proportion of Ambiguous Clustering


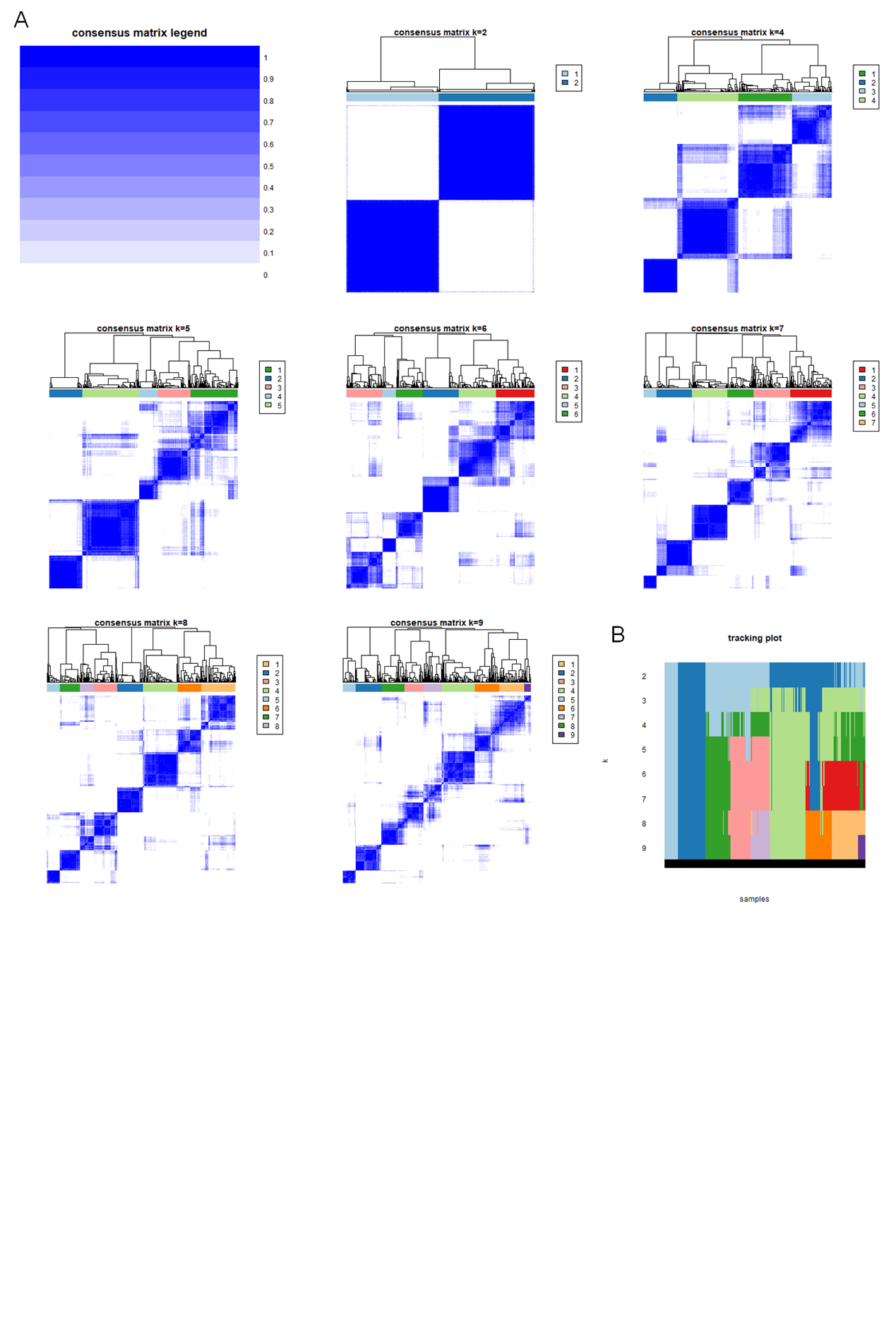


**Fig. S5** Consensus clustering of TCGA-STAD samples

**(A)** Consensus matrices for k = 2 and k = 4–9 (the selected k=3 matrix is shown in Figure S6), with darker blue indicating higher pairwise consensus. **(B)** Tracking plot displaying sample assignment dynamics across different k values.


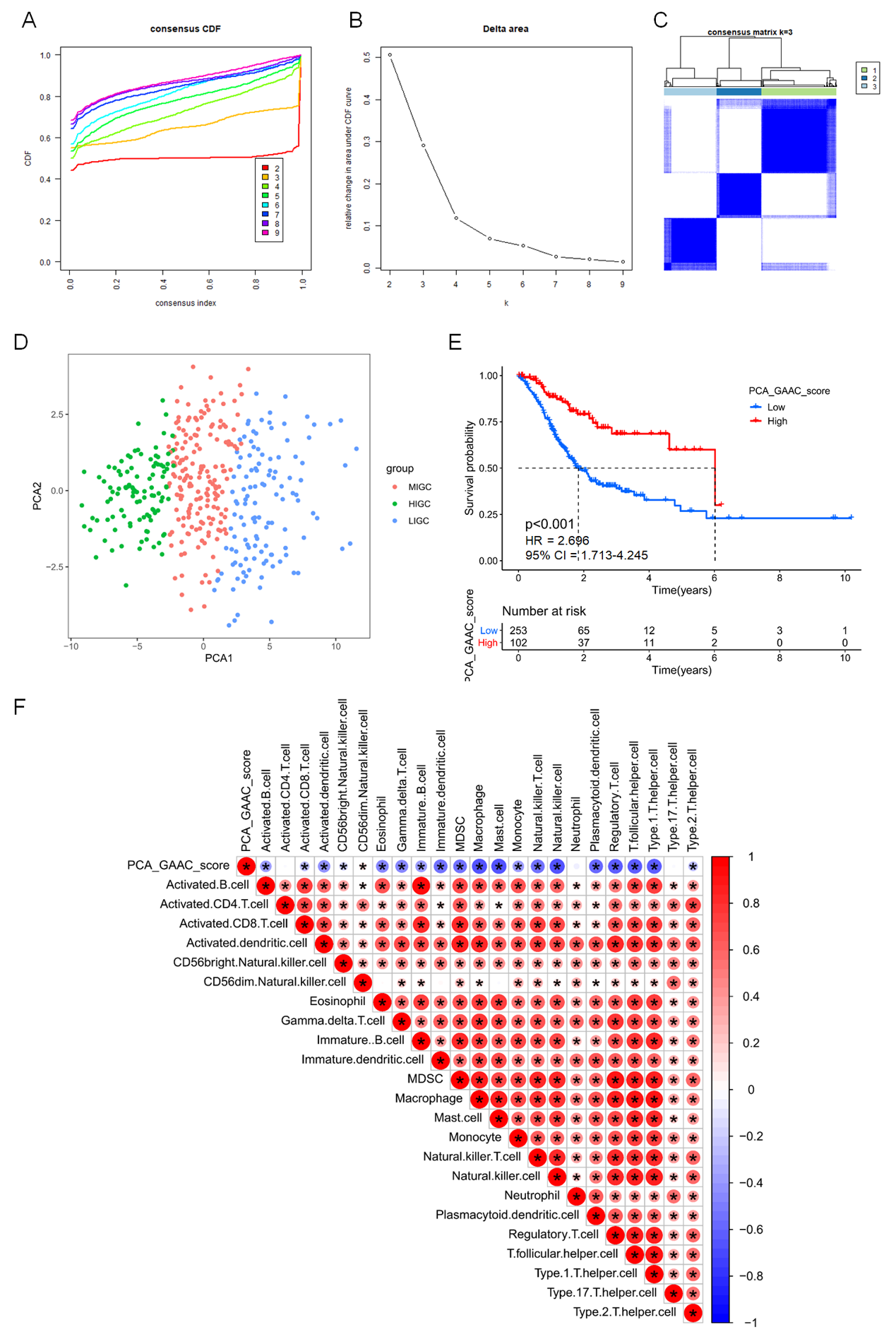


**Fig. S6** Consensus clustering and PCA analyses for patient stratification and prognostic assessment

**(A)** The CDF curve showed the dynamic change of CDF with increasing consensus index as k varied from 2 to 9. **(B)** The delta area curve displayed the relative change in area under CDF curve, varying k from 2 to 9. **(C)** The consensus matrix demonstrated the results of consensus clustering when k = 3. **(D)** The result of PCA analysis was illustrated, indicating the clear spatial separation among the HI-STAD, MI-STAD, and LI-STAD groups. **(E)** Low PCA scores of patients signified significantly shorter OS (p < 0.001). **(F)** It indicated that PCA scores were negatively correlated to the infiltration of most immune cells.

STAD-MSC, stomach adenocarcinoma myeloid-state classification; STAD, stomach adenocarcinoma; PCA, principal component analysis; LI-STAD, low immune infiltration STAD; MI-STAD, moderate immune infiltration STAD; HI-STAD, high immune infiltration STAD; OS, overall survival;


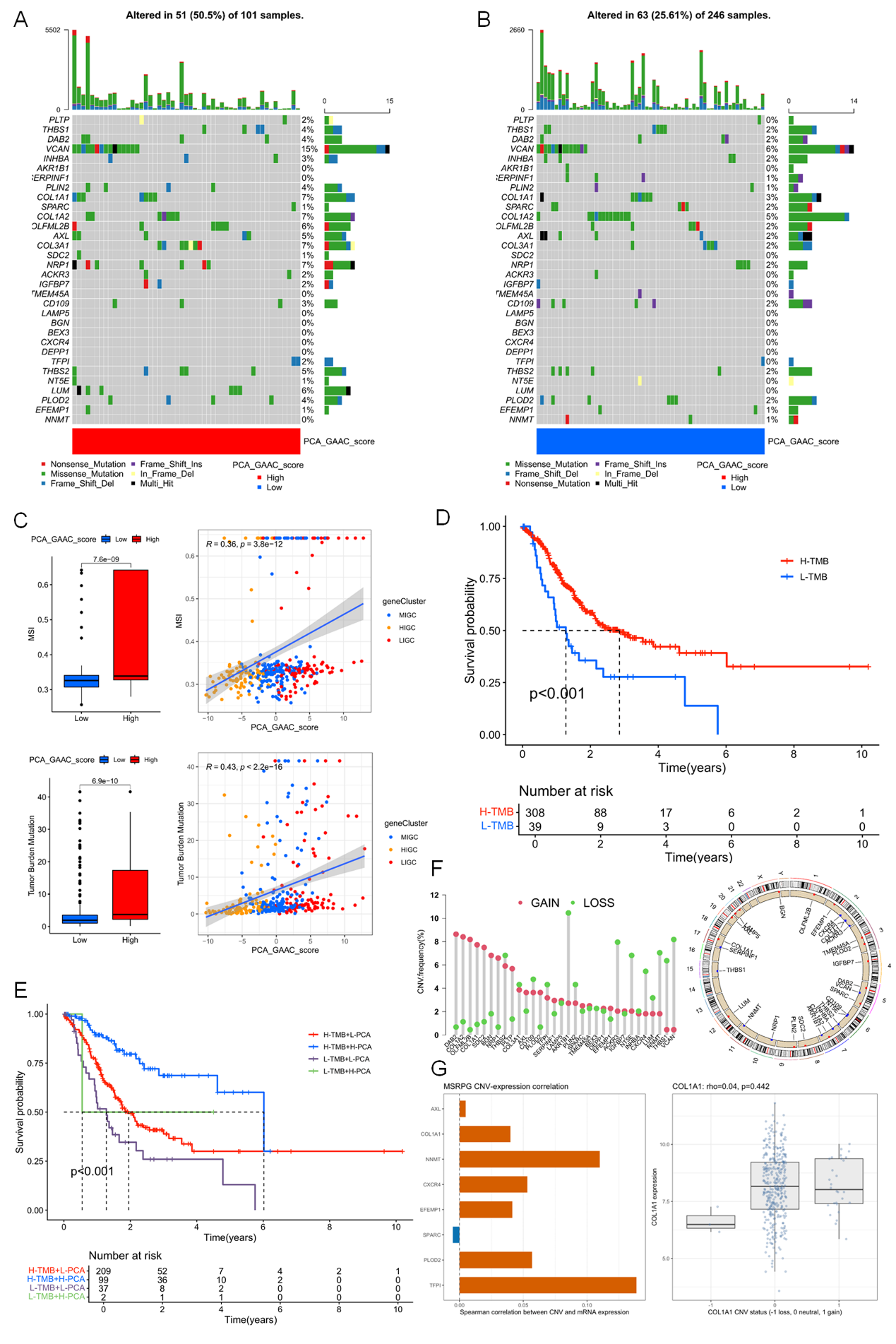


**Fig. S7** Genomic mutation landscape, tumor mutational burden, and survival analysis in high- and low-PCA patient subgroups

**(A)** The waterfall plot showed the 6 mutation types for the top 20 mutated genes in H-PCA, where genomic mutation was found in 51 (50.5%) of 101 patients. **(B)** The waterfall plot depicted the 6 mutation types for the top 20 mutated genes in L-PCA, where genomic mutation was identified in 63 (25.61%) of 246 patients. **(C)** The boxplots displayed significant differences (p < 0.01) for both TMB and MSI (p < 0.01) between H-PCA and L-PCA. Significant linear relationship was found between TMB (p < 0.01) or MSI (p < 0.01) with PCA score. **(D)** Patients with high TMB had significantly better OS (p < 0.001). Compared with H-TMB subgroup, L-TMB subgroup showed an increased risk of death [HR = 2.101, 95% CI = 1.374-3.215], **(E)** Kaplan-Meier survival curve unveiled significant prognostic differences (p < 0.001) among the four subgroups. Specifically, H-TMB and H-PCA subgroup exhibited the best prognosis. Cox proportional hazards analysis was performed using H-TMB and H-PCA subgroup as the reference group. Compared with H-TMB and H-PCA subgroup, L-TMB and H-PCA subgroup showed an increased risk of death [HR = 2.230, 95% CI = 0.299-16.643], L-TMB and L-PCA subgroup showed the highest risk of death [HR = 4.473, 95% CI = 2.502-7.999], H-TMB and L-PCA subgroup showed an intermediate risk [HR = 2.651, 95% CI = 1.653-4.252]. **(F)** The bar plot illustrated the CNV frequency of the 32 MSRPGs, which indicated the net gains or losses of part or whole chromosomal regions, while the circular plot visualized the chromosomal position of the 32 MSRPGs, along with their CNV (gains in red, losses in green, and both gains and losses in black). (G) Spearman correlations between CNV and mRNA expression for key MSRPGs were generally weak to modest. COL1A1 expression across CNV statuses (-1, 0, 1) confirmed a weak and non-significant association (rho=0.04, p=0.442). Collectively, these results indicate that CNVs alone cannot fully account for MSRPG upregulation, suggesting contributions from transcriptional, epigenetic, and microenvironmental regulators.

H-PCA, high PCA score group; L-PCA, low PCA score group; TMB, tumor mutation burden; MSI, microsatellite instability; L-TMB, low TMB group; H-TMB, high TMB group; CNV, copy number variation; MSRPGs, myeloid state-related prognostic genes;
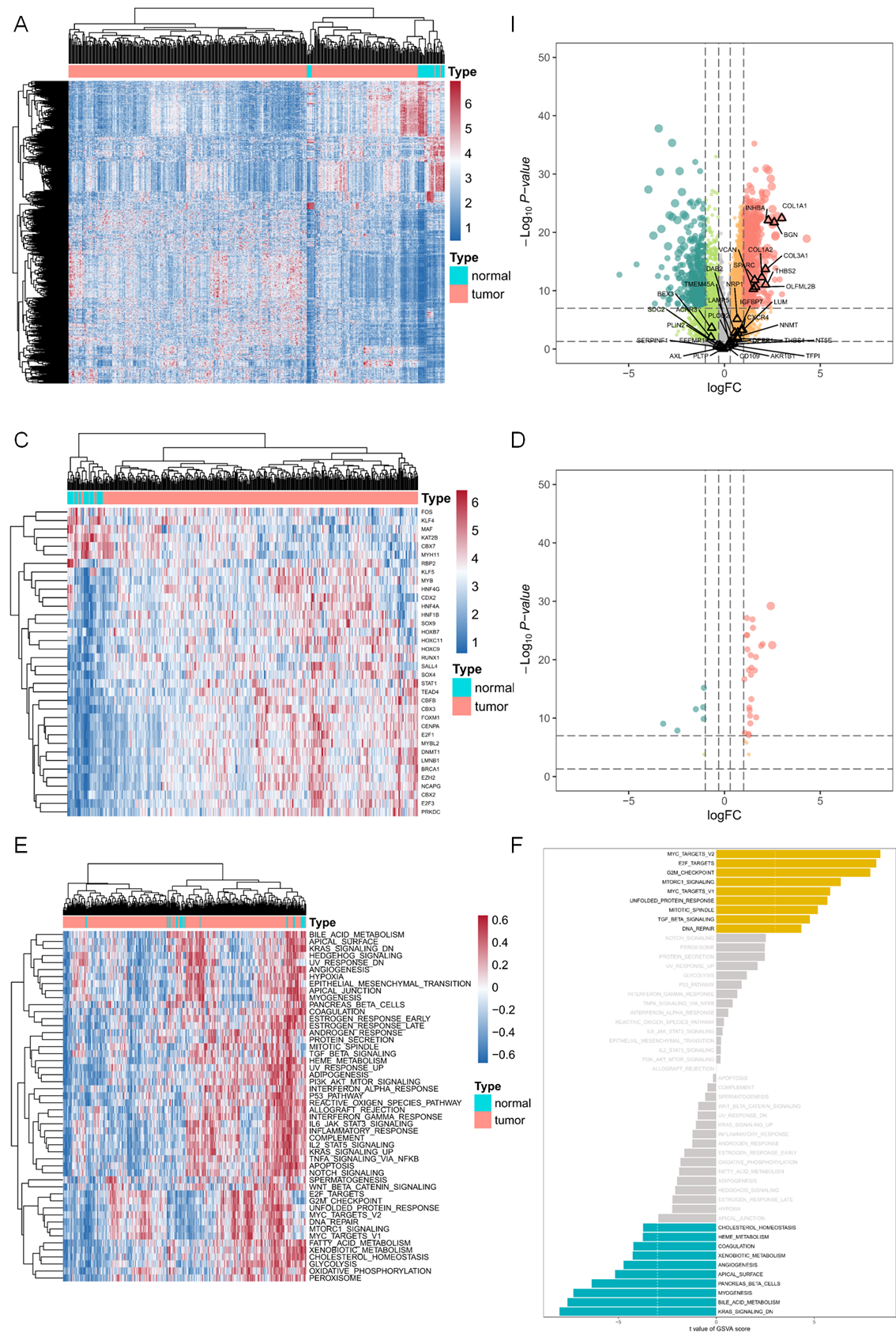


**Fig. S8** Transcriptomic and pathway analyses comparing tumor and adjacent normal tissues

**(A)** The heatmap showed DEGs between tumor and adjacent normal tissues. **(B)** The volcano plot demonstrated all the DEGs, and specifically pointed out all the MSRPGs. **(C)** The heatmap displaying DETFs between tumor and adjacent normal tissues. **(D)** Volcano plot depicted DETFs between tumor and adjacent normal tissues. **(E)** The heatmap revealed the differentially activated 50 cancer hallmarks, which was measured by GSVA between tumor and adjacent normal tissues. **(F)** The GSVA scores were quantified, with activated and inhibited cancer hallmarks presented at the top and bottom respectively. MYC targets v2, E2F targets, G2M checkpoint and Kras signaling dn, bile acid metabolism, myogenesis were respectively the most activated and inhibited cancer hallmarks in tumor tissues.

STAD, stomach adenocarcinoma; DEGs, differentially expressed genes; DETFs, differentially expressed transcription factor; GSVA, gene set variation analysis;
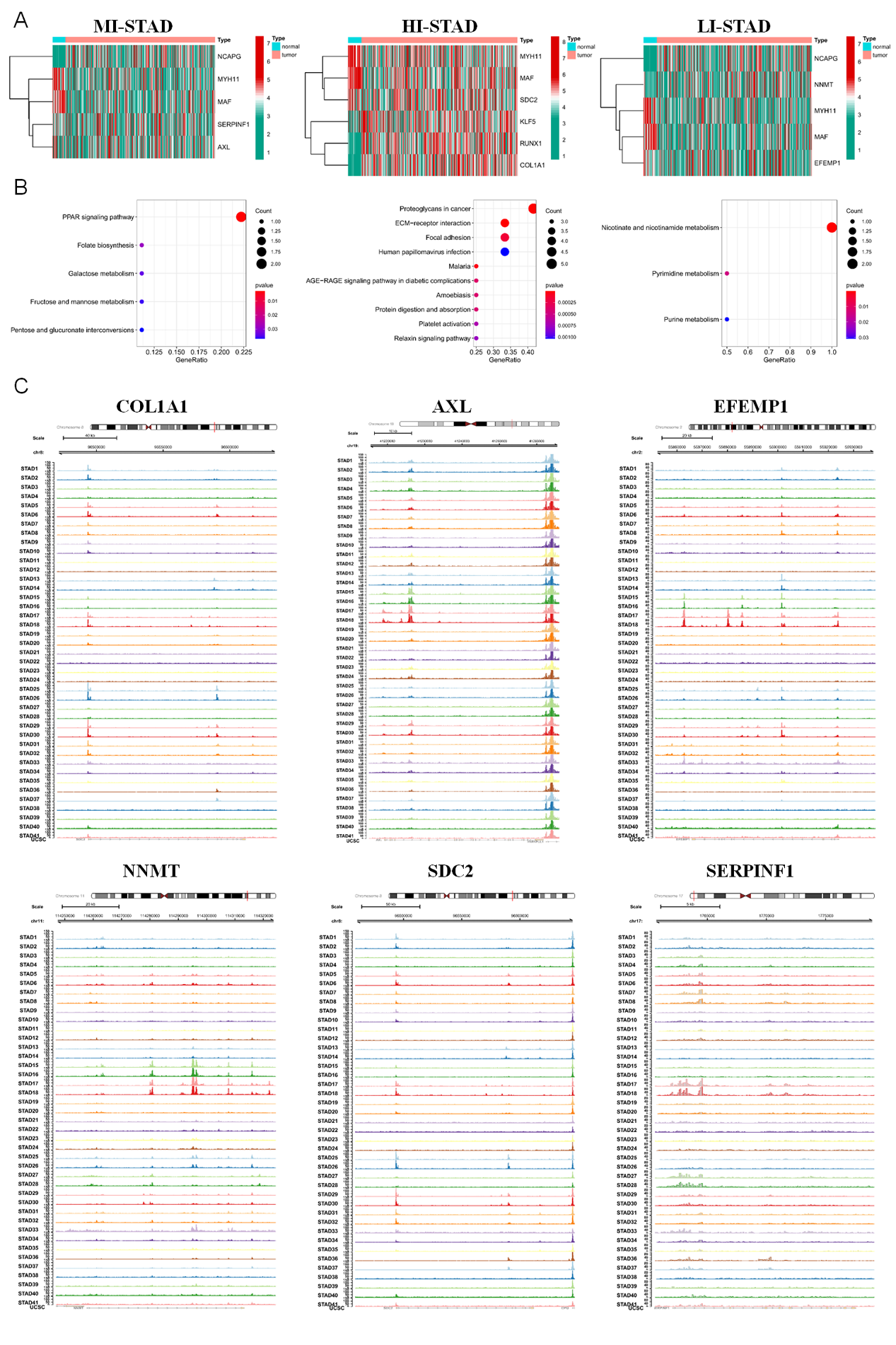


**Fig. S9** Comparative analysis of regulatory networks and pathway enrichment across STAD molecular subtypes

**(A)** The heatmaps illustrated and compared the expression levels of DETFs and MSRPGs in the three regulatory networks for the three STAD subtypes. **(B)** The KEGG bubble plots displayed the most significant pathways for the three STAD subtypes. **(C)** ATAC-seq tracks provided descriptive evidence of accessible chromatin regions near the six key regulatory MSRPGs (COL1A1, AXL, EFEMP1, NNMT, SDC2, SERPINF1).

ATAC-seq, assay for transposase-accessible chromatin sequencing; STAD, stomach adenocarcinoma; STAD-MSC, stomach adenocarcinoma myeloid-state classification; KEGG, Kyoto encyclopedia of genes and genomes; DETFs, differentially expressed transcription factor;
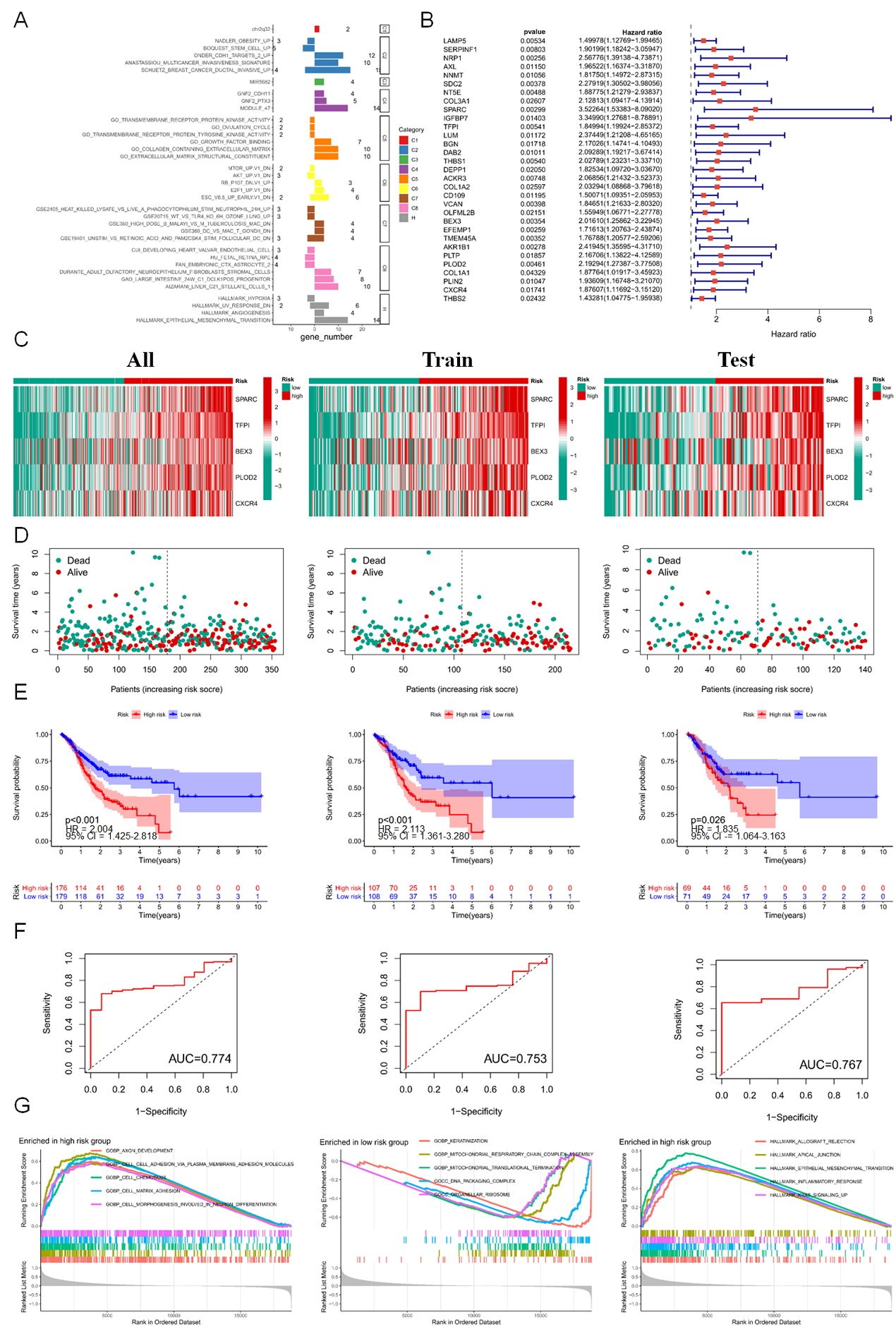


**Fig. S10** Functional enrichment, prognostic modeling, and biological characterization of MSRPGs in stomach adenocarcinoma

**(A)** Bar plots showing ORA of the 32 MSRPGs were mostly enriched in extracellular matrix organization, epithelial-mesenchymal transition, and specific invasive cancer signatures. **(B)** The forest plot depicted the results of the univariate proportional hazard Cox regression analysis of the 32 MSRPGs, including the p values, HR, and 95% CI. **(C)** Three heatmaps visualized the differential expression of the 5 MSRPGs between high- and low-risk subgroups in all, train, and test sets. **(D)** Three scatter plots intuitively demonstrated that increasing risk scores were possibly associated with higher probability of death in all, train, and test sets. **(E)** Kaplan-Meier analysis revealed significant survival differences between the high- and low-risk subgroups in the all (p < 0.001) and the training set (p < 0.001). The difference also remained statistically significant in the test set (p = 0.026), with the high-risk group showing worse prognosis. **(F)** The ROC curve indicated that the AUC of the MSRPGs-related model (0.774, all set; 0.753, train set, 0.767, test set) had moderate discriminatory performance. **(G)** GOBP functional enrichment analysis suggested heightened activity in axon formation and outgrowth, intercellular adhesion, chemotactic cell migration, cell-extracellular matrix adhesion, and neuronal morphogenesis within the high-risk group. GOBP functional enrichment analysis unveiled that epithelial keratinization, mitochondrial respiratory chain complex assembly, mitochondrial translation termination, DNA packaging complex formation, and organellar ribosome function within the low-risk group. Hallmark functional enrichment analysis revealed that immune-mediated transplant rejection, apical junction complex organization, epithelial-to-mesenchymal transition, inflammatory response, and KRAS oncogene signaling upregulation were highly upregulated in the high-risk group.

MSRPGs, myeloid state-related prognostic genes; ORA, over-representation analysis; ROC, receiver operating characteristic; AUC, area under the curve;


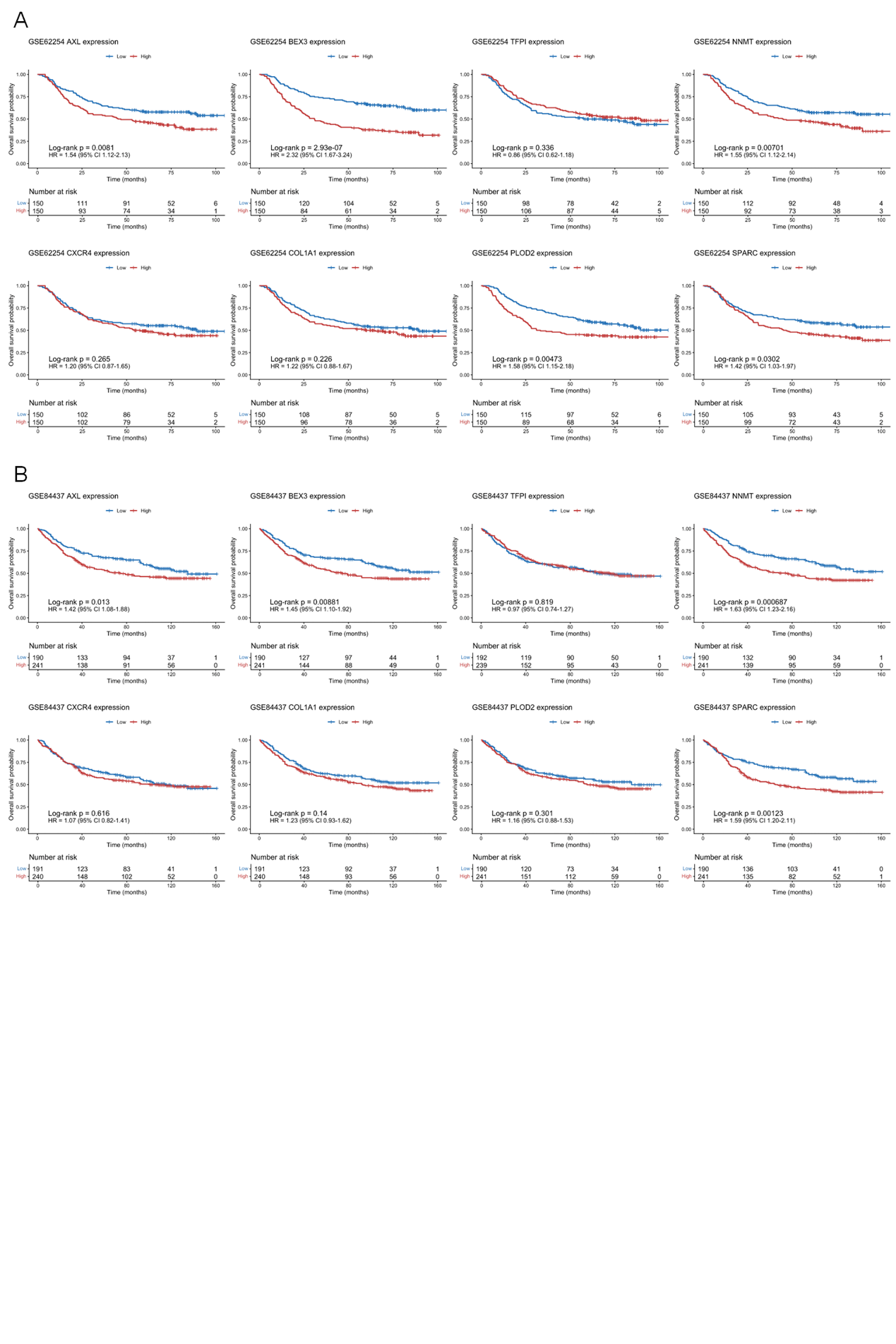


**Fig S11** Prognostic evaluation of individual genes in the validation cohorts.

**(A)** KM survival curves for each of the remaining genes in the GSE62254 and GSE84437 datasets. Patients were dichotomised by the median expression level of each gene, and overall survival was compared between the high‑ and low‑expression groups. HR and 95% confidence intervals were estimated using univariate Cox regression, and log‑rank P values are shown.

KM, Kaplan-Meier; CI, confidence intervals;


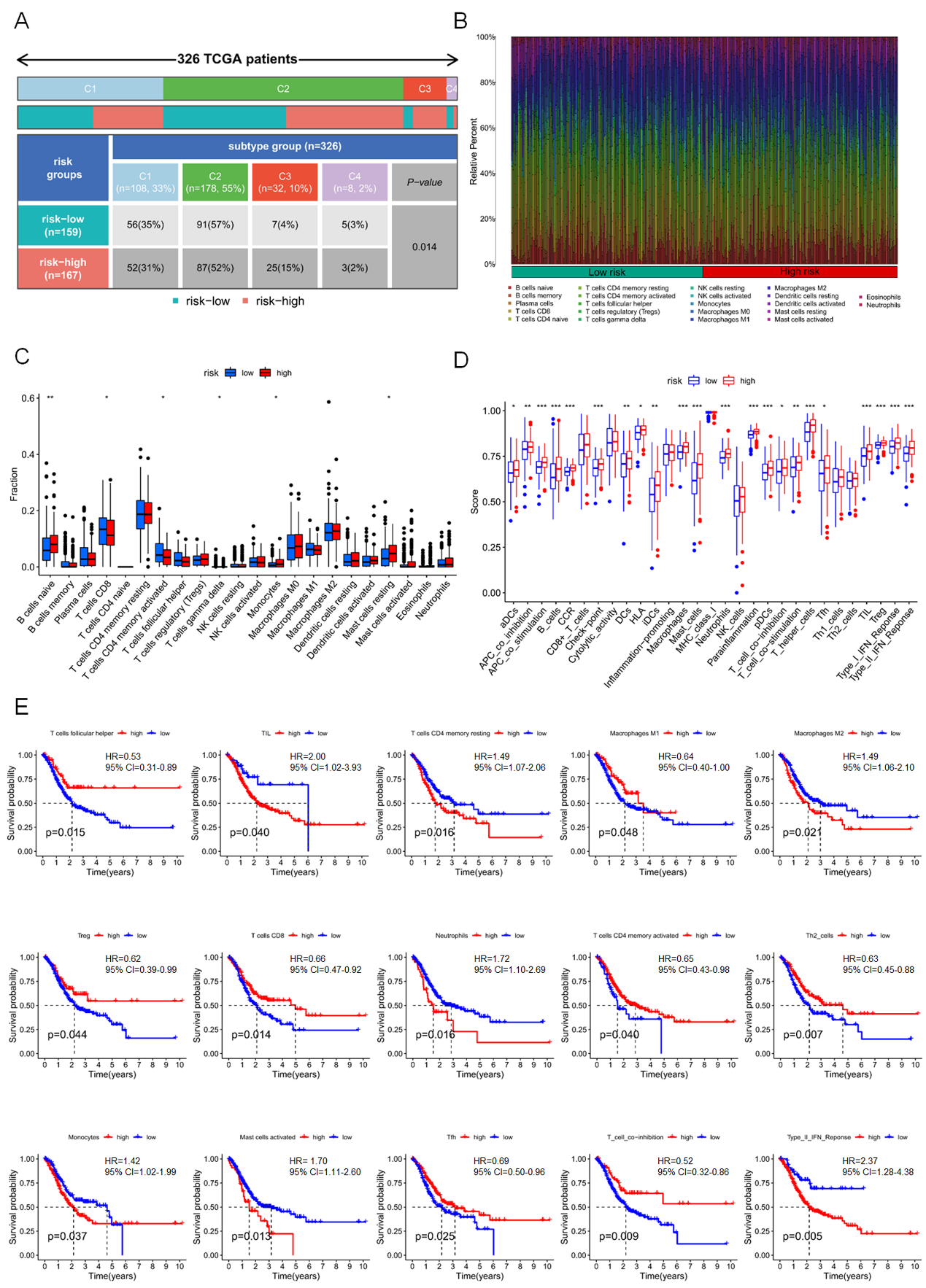


**Fig. S12** Immunological relevance of the MSRPGs-based risk prediction model by immune subtype analysis and differential analysis for immune components

**(A)** The cross table visualized the distribution of immune subtypes (C1-C4) between high- and low-risk groups, with a statistically significant difference detected by the Chi-square test (p = 0.014). **(B)** The boxplots showed the results of CIBERSORT analysis, which calculated all the immune components of high- and low-risk patients. **(C)** The boxplots showed that the fractions of T cells CD8 (p < 0.05), T cells CD4 memory activated (p < 0.05), and T cells gamma delta (p < 0.05) were higher in low-risk group, while the fractions of B cells naive (p < 0.01), monocytes (p < 0.05), and mast cells resting (p < 0.05) were higher in high-risk group. **(D)** The boxplots showed that the scores of aDCs (p < 0.05), APC co inhibition (p < 0.01), APC co stimulation (p < 0.001), B cells (p < 0.001), CCR (p < 0.001), checkpoint (p < 0.001), DCs (p < 0.01), HLA (p < 0.05), iDCs (p < 0.01), macrophages (p < 0.001), mast cells (p < 0.01), neutrophils (p < 0.001), parainflammation (p < 0.001), pDCs (p < 0.001), T cell co-inhibition (p < 0.05), T cell co-stimulation (p < 0.01), T helper cells (p < 0.001), Tfh (p < 0.05), TIL (p < 0.001), Treg (p < 0.001), Type I IFN response (p < 0.001), Type II IFN response (p < 0.001) were all higher in the high-risk group. **(E)** KM survival plots further unveiled significant differences of OS between high- and low-fraction of immune cells. Higher fraction of T cells follicular helper (p = 0.015), T cells CD4 memory activated (p = 0.040) and macrophage M1 (p = 0.048) were associated with better prognosis, while higher fraction of T cells CD4 memory resting (p = 0.016) and macrophage M2 (p = 0.021) were associated with worse prognosis. **(F)** KM survival plots also revealed significant differences of OS between high and low enrichment score of immune functions. Higher score of Treg (p = 0.044), T cells CD8 (p <= 0.014), Th2 cells (p = 0.007), T cell co-inhibition (p = 0.009) and Tfh (p = 0.025) were linked to higher survival probability, while higher score of neutrophils (p = 0.016), TIL (p = 0.040), Monocytes (p = 0.037), mast cells activated (p = 0.013) and Type II IFN (p = 0.005).

MSRPGs, myeloid state-related prognostic genes; KM, Kaplan-Meier;
